# Enteric Neural Crest Differentiation in Ganglioneuromas Implicates Hedgehog Signaling in Peripheral Neuroblastic Tumor Pathogenesis

**DOI:** 10.1371/journal.pone.0007491

**Published:** 2009-10-16

**Authors:** Timothy R. Gershon, Arash Shirazi, Li-Xuan Qin, William L. Gerald, Anna M. Kenney, Nai-Kong Cheung

**Affiliations:** 1 Department of Pediatrics, Memorial Sloan Kettering Cancer Center, New York, New York, United States of America; 2 Department of Epidemiology and Biostatistics, Memorial Sloan Kettering Cancer Center, New York, New York, United States of America; 3 Department of Pathology, Memorial Sloan Kettering Cancer Center, New York, New York, United States of America; 4 Department of Cancer Biology and Genetics, Memorial Sloan Kettering Cancer Center, New York, New York, United States of America; Institute of Cancer Research, United Kingdom

## Abstract

Peripheral neuroblastic tumors (PNTs) share a common origin in the sympathetic nervous system, but manifest variable differentiation and growth potential. Malignant neuroblastoma (NB) and benign ganglioneuroma (GN) stand at opposite ends of the clinical spectrum. We hypothesize that a common PNT progenitor is driven to variable differentiation by specific developmental signaling pathways. To elucidate developmental pathways that direct PNTs along the differentiation spectrum, we compared the expression of genes related to neural crest development in GN and NB. In GNs, we found relatively low expression of sympathetic markers including adrenergic biosynthesis enzymes, indicating divergence from sympathetic fate. In contrast, GNs expressed relatively high levels of enteric neuropeptides and key constituents of the Hedgehog (HH) signaling pathway, including Dhh, Gli1 and Gli3. Predicted HH targets were also differentially expressed in GN, consistent with transcriptional response to HH signaling. These findings indicate that HH signaling is specifically active in GN. Together with the known role of HH activity in enteric neural development, these findings further suggested a role for HH activity in directing PNTs away from the sympathetic lineage toward a benign GN phenotype resembling enteric ganglia. We tested the potential for HH signaling to advance differentiation in PNTs by transducing NB cell lines with Gli1 and determining phenotypic and transcriptional response. Gli1 inhibited proliferation of NB cells, and induced a pattern of gene expression that resembled the differential pattern of gene expression of GN, compared to NB (p<0.00001). Moreover, the transcriptional response of SY5Y cells to Gli1 transduction closely resembled the transcriptional response to the differentiation agent retinoic acid (p<0.00001). Notably, Gli1 did not induce N-MYC expression in neuroblastoma cells, but strongly induced RET, a known mediator of RA effect. The decrease in NB cell proliferation induced by Gli1, and the similarity in the patterns of gene expression induced by Gli1 and by RA, corroborated by closely matched gene sets in GN tumors, all support a model in which HH signaling suppresses PNT growth by promoting differentiation along alternative neural crest pathways.

## Introduction

Peripheral neuroblastic tumors (PNTs) comprise a spectrum of neural crest (NC) derived neoplasms that occur along the sympathetic chain, ranging in state of differentiation and malignancy. Ganglioneuromas (GNs) are benign tumors at the most differentiated end of the spectrum, composed of large neuronal cells, surrounded by satellite cells resembling glia. Neuroblastomas (NBs) span the rest of the spectrum, varying in malignancy and displaying variable degrees of neural and glial differentiation [1]. Clinically, the position of a PNT along the spectrum is not invariably fixed, as metastatic, poorly differentiated tumors can undergo spontaneous regression. When these tumors regress, they may necrose or transform into GNs [2]. PNTs thus demonstrate a dynamic inverse correlation between differentiation and malignancy, suggesting that inducing differentiation may be a potent therapeutic strategy [3].

Developmental signaling pathways may regulate differentiation in PNTs. To identify candidate developmental signals, we analyzed differential expression of markers and determinants of neural crest differentiation in GN and NB. We have previously demonstrated that transcription factors advancing NC development are expressed at higher levels in GN than NB [4]. Using transcriptome-wide microarray analysis of GN and NB, we have found that GNs, characterized by their advanced glial and neuronal differentiation, diverge from the sympathetic phenotype of their sites of origin. These highly differentiated, stroma-rich tumors express genes typical of enteric neural and glial fate and function, including key elements on the Sonic Hedgehog (SHH) pathway. We propose that Hedgehog pathway activity re-directs PNTs from a sympathetic nervous system (SNS) differentiation trajectory to an alternative neural crest trajectory, that of the enteric nervous system (ENS).

SHH is a potent regulator of development throughout the nervous system, and a potential oncogene. SHH is a mitogen for diverse CNS progenitors, including cerebellar granule cell precursors [5], and cells of the subventricular zone of the cerebrum [6]. In the cerebellum, mutations that enhance HH signaling are oncogenic, driving the formation of human medulloblastomas, both sporadic and syndromic [7], and murine medulloblastomas in transgenic Ptc+/− and SmoA1 mice [8], [9]. During peripheral neuro-ectodermal development, SHH is critical for specific differentiation of NC cells of the cranium and the gut [10], [11].

As NC cells emerge from the dorsal neural tube, they are outside the domain of SHH emanating from the ventral floor plate [12]. Cranial NC cells, however, encounter SHH as they migrate. HH signaling induces cranial NC cells to diverge from typical neuroectodermal fate, giving rise to tissues characteristically of mesodermal origin, including cranio-facial cartilage and bone, and the cardiac outflow tracts [10]. NC cells from the vagal region migrate into the embryonic gut and encounter SHH produced by endodermal cells. For cranial and enteric NC cells, SHH acts as mitogen and morphogen, regulating both proliferation and differentiation [11], [13].

While HH signaling is essential for NC development, and is oncogenic in CNS progenitors, a role for HH signaling in PNTs has not previously been defined. Our finding of increased HH activity in GN suggested that the HH pathway might promote PNT differentiation. To test the potential of HH signaling to effect PNT behavior, we transduced NB cells with *Gli1*, a central effector of the transcriptional response to HH signaling. We then analyzed *Gli1*-transduced cells for effects on cell growth and gene expression and compared the set of genes induced in NB cells by *Gli1* to the sets of genes induced by retinoic acid (RA) or differentially expressed in GN compared to NB. Our findings demonstrate HH signaling can re-direct developmental potential in PNTs, away from typical SNS phenotype, promoting alternative differentiation.

## Results

### Differential gene expression

Analysis of gene expression by microarray of 79 PNTs, including 11 GNs and 68 NBs, strongly differentiated the two sets of tumors. For each gene in the array, we compared expression values for GNs and NBs by modified t-test [14]; 2500 probe sets representing 2107 genes were differentially expressed in GN and NB with a p-value of <0.0001 (Table S1). Among these differentially expressed genes, several patterns emerged, demonstrating that GNs differ from NBs not only in extent of differentiation but also in specification within the range of potential NC fates.

### Catecholamine biosynthesis enzymes and sympathetic neuropeptides are expressed more strongly by NB than GN

Consistent with SNS phenotype, NBs expressed comparatively high levels of catecholamine biosynthesis enzymes tyrosine hydroxylase (TH), dopa-decarboxylase (DDC) and dopamine beta-hydroxylase (DBH; Table 1). These proteins are strongly expressed by sympathetic neurons. Chromogranin A and B (CHGA, CHGB) and neuropeptide Y (NP-Y) are similarly expressed throughout the sympathetic chain [15] and, in our analysis, were specifically expressed in NBs (Table 1). The relatively high expression of sympathetic markers in NB was confirmed by qRT-PCR. GNs, in contrast, expressed these sympathetic markers poorly (Table 1).

**Table 1:** Measured abundance of mRNA for select genes identified by microarray analysis as differentially expressed in GN (italics) or NB (bold).

		t-Test	Fold change	Fold change
		microarray	qRT-PCR	qRT-PCR
Probe set	Gene	p-value	NB/GN	GN/NB
*40808_s_at*	*CHGA*	*4.4E-11*		
*33426_at*	*CHGB*	*6.3E-11*	*7.4*	
*32943_at*	*DBH*	*6.6E-10*	*3.2*	
*32300_s_at*	*TH*	*2.3E-05*	*38.2*	
*38604_at*	*NP-Y*	*0.0009*	*10.6*	
*39668_at*	*TFAP2B*	*2.5E-08*	*198.1*	
**36299_at**	**CGRP**	**0.02**		**7.1**
**33567_at**	**VIP**	**0.04**		**12.5**
**40185_at**	**GFAP**	**0.01**		**7.5**
**1198_at**	**EDNRB**	**2.0E-24**		**9.2**
**33474_at**	**GLI1**	**0.004**		**28.7**
**40358_at**	**GLI3**	**3.0E-07**		**3.0**
**485_at**	**DHH**	**5.1E-10**		**10.8**
**1736_at**	**IGFBP6**	**2.1E-24**		**9.2**
**32154_at**	**TFAP2A**	**3.4E-13**		

**Figure 1:**
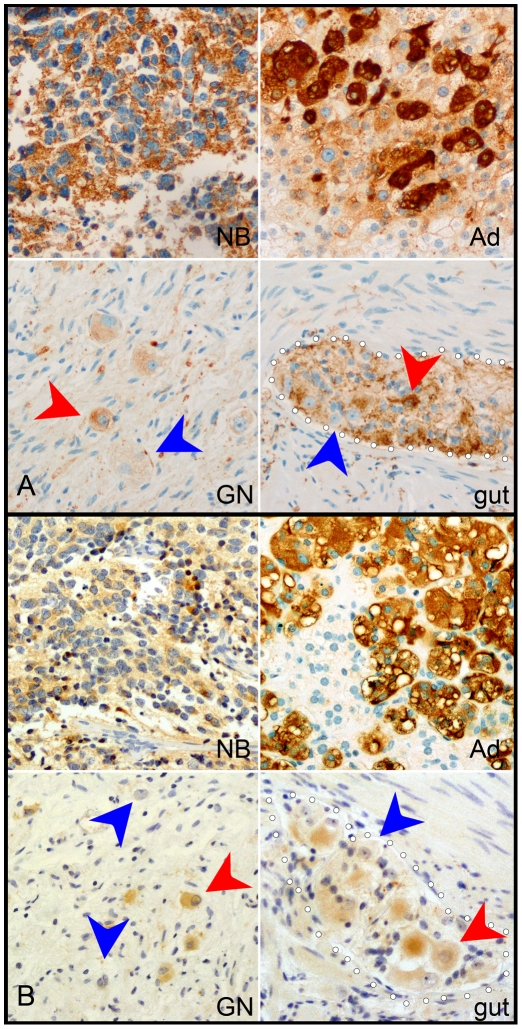
Adrenergic markers CHGA (A) and DBH (B) were expressed strongly in NB and weakly in GN. For each of these genes, immunocytochemistry produced intense labeling of NB, resembling the staining pattern of adrenal medulla (Ad). In contrast, in both GN and enteric ganglia, labeling was less intense, and was limited to a subset of neurons (red arrowheads), leaving other neurons unlabelled (blue arrowheads). Dotted white lines highlight the margins of the enteric ganglia.

We confirmed the differential expression of sympathetic markers in NB by immunocytochemistry for TH, DBH, CHGA and CHGB. All of these markers demonstrated similar expression patterns. Typical images of CHGA and DBH staining are shown in Figure 1. In NB and adrenal medulla, large clusters of cells strongly express TH and CHGA proteins in a cytoplasmic distribution. In both GN and enteric ganglia, expression was weaker and was limited to a subset of neurons, with many neurons demonstrating no expression. Immunostaining for CHGB appeared almost identical to CHGA, while TH strongly resembled DBH (data not shown).

### Differential expression of AP-2 transcription factors distinguish GN from the sympathetic lineage

Transcription factors *AP2-alpha* and *AP2-beta* are homologous genes expressed in succession during SNS development and differentially expressed in GN and NB. Pre-migratory NC cells express AP2-alpha and this protein is essential for all NC development to proceed [16]. In contrast, post-migratory sympathoblasts express AP2-beta, which is required for adrenergic differentiation [17]. Microarray analysis confirmed, as we have previously demonstrated, *AP2-alpha* is expressed at higher levels in GN compared to NB [4]. In contrast, NBs expressed markedly higher levels of *AP2-beta* (Table 2). The down-regulation of *AP2-alpha* and complementary up-regulation of *AP2-beta* demonstrates SNS commitment specific to NB and not found in GN.

**Table 2:** Genes regulated by the HH pathway in other systems were differentially expressed in GN relative to NB, consistent with observed HH activity in GN.

		t-Test
		microarray
Probe set	Gene	p-value
*1736_at*	*IGFBP6*	*2.1E-24*
*36650_at*	*cyclin D2*	*9.6E-29*
*37623_at*	*NR4A2*	*8.3E-23*
**2047_s_at**	**plakoglobin**	**4.4E-06**
**35084_at**	**AMH**	**3.0E-05**

Messages listed in italics were predicted to be up-regulated by HH activity *and* were more abundant in GN. Messages listed in bold were predicted to be down-regulated by HH activity *and* were less abundant in GN.

### Proteins specific to the enteric nervous system are enriched in GN

While GNs expressed low levels of sympathetic markers, genes abundant in the ENS were strongly expressed in GNs. To discern ENS from SNS phenotype we analyzed expression of calcitonin gene related peptide (CGRP), vasoactive intestinal peptide (VIP), glial acidic fibrillary protein (GFAP), and endothelin B receptor (EDNRB). *CGRP* and *VIP* are highly expressed in both enteric neurons and dorsal root ganglion cells [18], but only sporadically detectable in sympathetic neurons [19], [20] *GFAP* is widely expressed by enteric glia but uncommon in Schwann cells and limited to the non-myelinating subset [21], [22]. *EDNRB* is expressed during development in both sympathetic and enteric progenitors as well as melanoblasts but functionally required only for enteric and melanocytic differentiation [23], [24]. Microarray analysis and qRT-PCR demonstrated that *CGRP, VIP, GFAP* and *EDNRB* were all expressed at markedly higher levels in GN relative to NB (Table 1).

**Figure 2:**
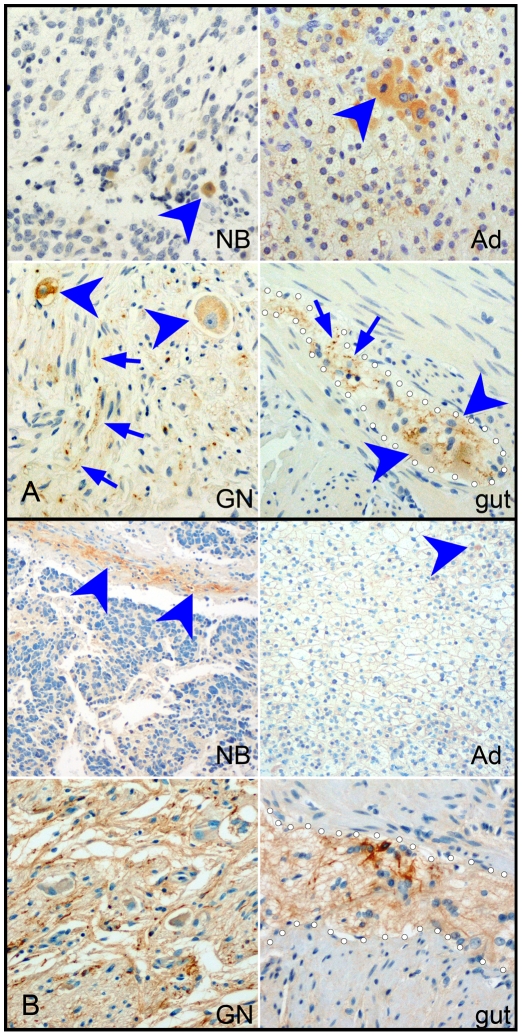
Enteric NC markers CGRP (A), and GFAP (B) were expressed more strongly in GN than NB. Antibodies to CGRP labeled rare cells (blue arrowheads) in NB and adrenal medulla (Ad), while labeling neurons (blue arrowheads) and processes (blue arrows) throughout both GNs and enteric ganglia (gut). Antibodies to GFAP labeled stromal cords in NB and rare adrenal medullary cells (blue arrowheads). In contrast, labeling was intense and widespread in GN and in ganglia of the gut (outlines by dotted white lines).

To demonstrate differential expression of ENS markers at the cellular level, we used immunocytochemistry to detect GFAP and CGRP in tumors and control tissues (Fig. 2). In NB, as in adrenal medulla, rare scattered cells expressed CGRP. In contrast, in GN, strong CGRP expression was detected in cell bodies of almost all ganglion cells and in neuritic processes. A similar pattern appeared in the ENS, with frequent cytoplasmic labeling of neurons, and intense labeling of axons (Fig. 2A). GFAP immunostaining in NB was restricted to narrow chords of stroma. GFAP labeling was also seen in rare cells of the adrenal medulla. GN and ENS, however, demonstrated extensive GFAP labeling with a similar fibrillary appearance (Fig. 2B). We have previously demonstrated robust expression of myelin proteins by glial cells in GN [4]; the combination of myelin proteins and GFAP distinguishes the glia of GN from non-myelinating Schwann cells, suggesting instead an enteric glial phenotype. Taken together, the differential expression of multiple markers in GN confirms divergence from the sympathetic differentiation trajectory, toward an ENS phenotype, an alternative NC fate.

### Hedgehog pathway ligand and transcription factors are expressed at high levels in GNs, with predicted effects on known hedgehog target genes

Three homologous transcription factors are activated by the HH pathway: *GLI1, GLI2 and GLI3*. Transcriptional activation of *GLI1* is a sensitive reporter of HH pathway activity[25]. Microarray and qRT-PCR demonstrated differential expression of *GLI1* and *GLI3* in GN (Table 1). Of the 3 HH ligands, SHH and IHH were not expressed at significantly different levels in GN or NB (data not shown). DHH, however, was specifically expressed in GN (Table 1).

To determine the impact of differential expression of HH pathway genes in GN we examined the expression of genes known to be regulated by HH signaling. Microarray analysis demonstrated up-regulation of established HH targets, including *IGFBP6* [26], *cyclin D2* [27] and *NR4a1* [28] (Table 2). To validate these observations, we used qRT-PCR to confirm up-regulation of *IGFBP6* in a second independent set of GN (Table 1). Genes known to be down-regulated by HH pathway activity, including *anti-mullerian hormone* (*AMH*) [28] and *plakoglobin* [29] were specifically down-regulated in the GNs (Table 2). The differential expression of HH targets in GN demonstrates the active influence of the HH pathway.

### *Gli1* alters the proliferation rate of NB cells

**Figure 3:**
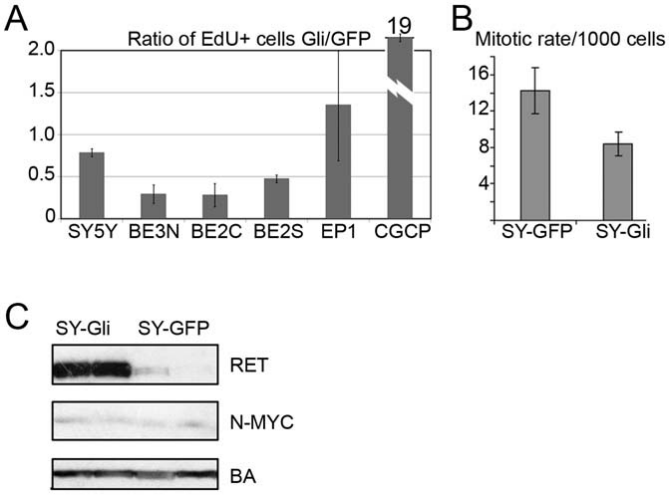
Gli1 slowed proliferation of NB cell lines while driving CGNPs to proliferate. A) Transduction of *Gli1* decreased proliferation of NB cell lines SY5Y, BE2(N), BE2(C) and BE2(S). Proliferation in SH-EP1 was relatively unaffected (P>0.05) while proliferation of CGCPs was increased 19-fold. Error bars represent standard deviation normalized mean. B) Decreased proliferation of SY-Gli cells relative to SY-GFP was confirmed by measuring the mitotic rate of both lines, expressed as PH3+ cells per 1000. Error bars represent standard deviation. C) Western blot of two independent SY-Gli and SY-GFP cultures demonstrated the N-MYC expression was not affected by *Gli1* transduction, while RET was strongly induced by Gli1.

To test whether HH pathway activity might direct PNT behavior, we transduced NB cell lines with *Gli1* together with GFP or with GFP alone, and then sorted transduced cells to >95% purity by FACS and analyzed proliferation rate and gene expression. We verified *Gli1* activity by transducing a GLI-activated luciferase reporter plasmid (data not shown). Transduction of *Gli1* profoundly affected the proliferation of NB cells growing in culture (Fig. 3a,b).

We studied effects of *Gli1* over-expression on five NB cell lines with different characteristic patterns of growth: the rapidly proliferating lines SH-SY5Y, BE2(S), BE2(N), BE2(C), and slow-growing cell line SH-EP1. *Gli1* reduced the proliferation rate of each of the rapidly growing lines, while minimally affecting the proliferation rate of SH-EP1 (Fig. 3a). In contrast, in explanted murine cerebellar granule cell progenitors (CGCPs) transduction with Gli1 increased proliferation rate 19-fold (Fig. 3a). To confirm that Gli1 reduced in proliferation of NB cells, we prepared and sorted to purity an independent set of SH-SY5Y cells transduced with Gli1 and GFP (SY-Gli) or with GFP only (SY-GFP) and quantified mitoses by PH3 immunostaining. SY-Gli1 cells demonstrated 50% decreased mitotic rate, compared to SY-GFP (Fig. 3b). Immunostaining for cleaved caspase-3, in contrast, demonstrated no increase in apoptosis in the two populations (data not shown).

### Gli1 induces NB cells to express genes directing NC development

We determined the transcriptional response of NB cells to *Gli1* by comparing gene expression of SY-Gli1 and SY-GFP by microarray hybridization. Diverse genes involved in NC development were differentially expressed. We found Gli1 induced differential expression of 193 out of 13,089 genes (228 out of 22,215 probe sets) on the U133 2.0 with a p-value of <0.0001 (Table S2). Of these 193 genes, we selected 12 developmentally relevant genes for confirmatory study by qRT-PCR, using independently prepared sets of transduced SY5Y cells raised in triplicate. qRT-PCR confirmed differential expression of 11 of 12 genes with a fold change of 2 or greater (Table 3). Up-regulation of only one of these 12 genes, SOX2, could not be confirmed. We also measured the expression of these 12 genes by qRT-PCR in SH-EP1 cells transduced with *Gli1* or GFP. 6 of the 12 genes tested were up-regulated by *Gli1* in SH-EP1 cells with a fold change of 2 or greater (Table 3), demonstrating both common elements in the transcriptional response to *Gli1*, and as well as elements that vary with the cellular context.

**Table 3:** Measured abundance of mRNA for select genes identified by microarray analysis as regulated by *Gli1* in SY5Y cells.

	SY5Y GLI/GFP	SY5Y GLI/GFP	SH-EP1 GLI/GFP
	p-value	fold change	fold change
	microarray	qRT-PCR	qRT-PCR
**MATN2**	**2.8E-12**	**28.9**	**625.4**
**KAL1**	**2.9E-11**	**176.3**	**10.9**
**PMP22**	**4.7E-09**	**2.0**	**2.0**
**IGFBP5**	**4.1E-08**	**3.1**	**1.6**
**NTRK2**	**1.3E-07**	**3.3**	**2.0**
**FDZ7**	**1.5E-07**	**2.0**	**1.3**
**EGFR**	**3.7E-07**	**3.0**	**1.4**
**WNT4a**	**4.5E-07**	**5.4**	**0.6**
**SOX2**	**3.2E-05**	**0.16**	**3.5**
EDNRA	2.2E-08	2.7	1.1
RET	1.5E-07	4.4	1.5
VMAT2	8.2E-05	81.2	2.1

Genes up-regulated in GN are listed in bold.

Among the 193 genes regulated by *Gli1* in SY5Y cells were crucial determinants of cranial and enteric NC development (Table 4). Cranial NC genes up-regulated by *Gli1* included *KAL1* [30], [31], *MSX2* [32] and *EDNRA* [33], [34]. Genes critical to ENS development that were induced by *Gli1* included *RET* [35] and *GFRA2* [36]. Gli1-regulated genes could also grouped by the different cell types they specified (Table 4): *KAL1, RET* and *GFRA2* regulate neural fates, while *MSX2* and *EDNRA* direct the development of bony NC derivatives. Gli1 induced glial differentiation marker *PMP22* and neuronal marker *VMAT2*, demonstrating the potential of Gli1 to advance both neural and glial differentiation (Table 4). The observed changes in proliferation and gene expression induced by Gli1 demonstrate the potential for HH signaling to alter the behavior and differentiation trajectory of NB cells.

**Table 4:** Genetic determinants of diverse NC fates are induced by *Gli1* in SY5Y cells.

Cranial NC genes	ENS genes	Neural NC fate	Bony NC fate	Glial NC fate
KAL1	RET	RET	NELL1	PMP22
EDNRA	GFRA2	GFRA2	EDNRA	
MSX2		VMAT2	MSX2	

### Gli1 advances differentiation of SY5Y cells

To test further whether Gli1 advanced the differentiation of NB cells in culture, we compared the transcriptional responses of SH-SY5Y cells to either Gli1 transduction or treatment with the differentiation agent RA. RNA from SY-GFP cells treated for 48 hours with either 10 uM all-trans retinoic acid (ATRA) or vehicle was analyzed by microarray and the resulting datasets were compared to determine the sets of genes differentially expressed in response to RA. Comparison of three replicates of each condition, with p<0.0001 defined a set of 326 genes differentially expressed in SY-Gli compared to SY-GFP, and a set of 2123 differentially expressed in SY-GFP treated with ATRA or vehicle. The overlap of these two sets was striking: 147 genes were regulated by both ATRA and Gli1 in the same direction (p<0.00001 by hypergeometric test). The close correspondence between transcriptional response to Gli1 and RA supports the interpretation that Gli1 drives NB cells toward differentiation.

The differential expression of HH pathway genes and targets in GN, the divergence of GN from SNS fate, and the induction of cranial and enteric NC genes in NB cells by Gli1, suggested that HH signaling might drive PNTs toward the GN phenotype. In order to test the hypothesis that HH activity could induce PNTs to become GNs, we determined whether the transcriptional response of NB cells to Gli1 resembled the specific gene expression pattern of GN. We compared the set of genes regulated by Gli1 in SY5Y cells to the set of genes differentially expressed in GN, compared to NB. We found highly significant overlap between the two sets. Of the 2108 genes found on the U95A array to be differentially expressed in GN with a p-value<0.0001, 1811 are represented on the U133 2.0 array and could be used for comparison. In our initial comparison of SY-Gli and SY-GFP, 193 of the 13,089 genes on the U133 2.0 array were differentially expressed with p<0.0001. Of these 193 genes, 57 genes were differentially expressed in GN compared to NB, a correlation very unlikely to occur by chance (p<0.00001 by hypergeometric test). Thus the effect of Gli1 transduction was to slow the proliferation of SH-SY5Y cells, and induce a transcriptional response that resembled both the response to RA and the specific gene expression pattern of GN.

### Absence of effect on N-Myc and up-regulation of RET by Gli1

The decreased proliferation and advanced differentiation of NB cells in response to Gli1 contrasted sharply with increased proliferation of Gli1-transduced CGCPs. These alternative responses to HH activity might be mediated by crucial, cell-specific differences in the underlying transcriptional response to Gli1. N-Myc is up-regulated by HH pathway activity in CGCPs and functions as an essential effector of Shh driven proliferation [37]. N-MYC is also an established oncogene in neuroblastoma. We have previously found that the RET proto-oncogene is up-regulated by RA in NB cell lines and plays an essential role in RA-induced differentiation [38]. We investigated the potential induction of N-MYC by Gli1 in PNTs by comparing N-MYC mRNA and protein levels in SY-Gli and SY-GFP cells. N-MYC mRNA expression measured by microarray did not correlate with Gli1 transduction (data not shown). Western blot (Fig. 3c) confirmed that N-MYC protein levels were not substantially altered by Gli1. In contrast, RET protein was strongly induced in SY-Gli cells, consistent with the promotion of differentiation.

## Discussion

### Differentiation in GN: SNS vs ENS phenotype

Transcriptome analysis of PNTs revealed divergent differentiation trajectories of GN and NB. Among the many differentially expressed genes, we were able to discern a clear shift in markers for NC subsets: NBs expressed sympathetic markers while GNs expressed a pattern of differentiation markers most consistent with the ENS. Like developing ENS cells, GNs demonstrated activity of the HH pathway, marked by expression of both GLI1 and GLI3 and modulation of known HH target expression in predictable directions.

The ENS phenotype of neurons and glia in GN represents divergence from the characteristic pattern of SNS development in PNTs. GNs typically occur at sites along the sympathetic chain, and their progenitor cells have thus migrated as sympathoblasts. At some point after migration, however, under the influence of HH, these progenitors differentiate as ENS neurons and glia. The phenomenon of GNs arising from sites of metastatic NB, moreover, indicates this divergence may occur after malignant transformation has taken place. While the characterization of GNs as resembling ENS is novel, it has long been known that children with GN may present with gastrointestinal complaints. Enteric dysmotility in these patients has been attributed to secretion by the tumor of enteric neuropeptides [39]. The present findings place this enteric presentation within the context of NC development.

### HH pathway as morphogenic and tumor suppressive

During development, HH is both a mitogen and a morphogen, regulating both proliferation and differentiation. The diverse potential effects of HH signaling allow for a range of plausible roles in GN pathogenesis. Although HH-mediated Gli1 activation can drive proliferation and tumorigenesis of cerebellar neural progenitors, and we found that Gli1 transduction increased the proliferation of CGCPs, in NB cells, Gli1 inhibited proliferation and induced expression of RA-responsive genes and genes up-regulated in GN. Our observation that *Gli1* decreased proliferation of NB cells is not consistent with an oncogenic effect.

We suggest that HH acts in GN as a morphogen, driving not proliferation, but specific fate choice, promoting PNT differentiation rather than growth. In the developing zebrafish retina, SHH signaling promotes the differentiation of neuroblasts, and inhibition of SHH supports progenitor proliferation [40]. In hippocampal neural stem cells, moreover, GLI1 can limit proliferation by inducing apoptosis [41]. Accordingly, GNs may demonstrate the highest differentiation specifically because they have achieved the highest levels of HH activity, depleting tumor stem cells through apoptosis or differentiation. While we did not observe prominent induction of apoptosis by SY-Gli cells, we cannot rule out an apoptotic effect on a sub-population of cells with stem-like properties. A tumor suppressive role for HH signaling would account for the benign, differentiated phenotype of GNs and for the decrease in proliferation of NB cells on transduction with *Gli1*. This hypothesis may be tested *in vivo*, and the role of HH signaling in NC development and PNT pathogenesis may be further elucidated, through xenograft studies and animal primary PNTs models, using targeted activation of Smo to drive HH activity [42] and targeted activation of MYCN to induce PNTs [43].

### DHH and Schwann cell mediated differentiation in GN

The mechanism of HH pathway activation in GN is not clear. The HH pathway may be activated through a cell autonomous mechanism, as seen in medulloblastomas in which mutant SMO is constitutively active [9]. We did not detect differential expression of SMO in GN, but the possibility of activating mutations in GN remains to be evaluated. Alternatively, the HH signaling may be activated by ligand binding.

HH signaling in GN could be mediated by DHH, which, like GLI1 and GLI3 is differentially expressed in these benign tumors. Schwann cells in peripheral nerves secrete DHH [44], and GNs are largely composed of cells that resemble Schwann cells. Several lines of evidence have demonstrated that Schwann cells secrete factors that promote differentiation of NB cells [45], [46]. DHH may contribute to this inductive interaction, and glial cells within GN may, unlike typical Scwhann cells [47], respond to DHH in an autocrine manner, to enforce and advance differentiation.

## Materials and Methods

### Human Tissue Samples

This study was conducted according to the principles expressed in the Declaration of Helsinki. The study was approved by the Memorial Sloan-Kettering Cancer Center Institutional Review Board (IRB) and Human Tumor Utilization Committee (HTUC). All patients provided written informed consent for the collection of samples and subsequent analysis.

### Cell culture techniques

Neuroblastoma cell lines were kindly provided by R. Ross and B. Spengler (Fordham University, Fordham, NY). HEK293E cells (Invitrogen) were used for retroviral packaging. All cell lines were maintained in Opti-MEM with Glutamax (cat#51985; Invitrogen) supplemented with 10% FCS and penicillin/streptomycin at 37°C in a humidified environment with 5% CO_2_. For RA treatment, SY-GFP cells were plated in triplicate sets in 10 cm plates and allowed to adhere for 24 hours, after which 1/1000^th^ volume of 10 mM retinoic acid dissolved in 100% EtOH, or vehicle alone was added to the medium. After 48 hours in ATRA, cells were processed for microarray.

Murine *Gli1* and GFP were transduced into NB cells using a bi-cistronic retroviral plasmid, pLZIR-GLI (gift of Robert Wechsler-Reya, PhD, Duke University). Control cells were transduced with GFP, using pWZL-GFP. Retroviral stocks were prepared by co-transfecting HEK293E cells with Gag-Pol and VSVG plasmids along with the either pLZIR-GLI or pWZL-GFP, using Fugene 6.0 (cat#11814443001; Roche). Packaging cells were transferred to 32 C 24 hours after transfection, and viral stocks were harvested at 48 and 36 hours. For transduction, NB cells were exposed to retroviral stock at 32 C for 4–6 hours, recovered for 3–4 days at 37 C and sorted for GFP expression by FACS. GFP+ populations were then recovered and expanded through 2–3 passages before use in experiments.

Proliferating cells were labeled by EdU and Alexa647 using the Invitrogen Click-iT EdU Alexa Fluor 647 Flow Cytometry Assay kit (cat# A10202), then analyzed by FACS. For PH3 and caspase staining, 50,000 cells per well were plated in 96 well plates and maintained in culture for 2 days, then fixed in 4% formaldehyde, immunostained for PH3 and counterstained with DAPI. We performed automated quantification of PH3+ and DAPI+ cells with 8-fold replicates using an INCell 1000 automated microscope (GE/Amersham Biosciences).

### Microarray analysis

Microarray hybridization of tumor derived samples was carried out on Affymetrix U95A chips, as previously described [48]. RNA was prepared from SY-Gli and SY-GFP cell lines using Trizol Reagent (Invitrogen cat# 15596026), processed and analyzed on Affymetrix U133 2.0 chips, according to manufacturer's protocol. All microarray expression data were processed with Robust Multichip Analysis (RMA; R software; the R Foundation for Statistical Computing) [49]. Differential expression analysis was performed to identify genes between sample groups applying an Empirical Bayes t test to each gene [14]. All microarray data are described in accordance with MIAME guidelines. Microarray data are deposited in the caArray Array Data Management System, Experiment Identifier gersh-00292.

To derive gene lists for comparison, a p-value cutoff of 0.0001 was used to select differentially expressed genes. The number of tests (i.e. probesets) with p<0.0001 expected by chance equals 0.0001× total number of probesets. For the U95 platform, which is used for the tumor sample study, the total of number of probesets is 12,533 and hence 1 probeset is expected to have a p-value<0.0001 just by chance. For the U133A platform, which is used for the cell line study, the total of number of probesets is 22,215 and hence 2 probesets are expected to have a p-value<0.0001 just by chance. The actual sets of genes demonstrating differential expression with p<0.0001 were compiled, and a hypergeometric test was then used to calculate the probability of overlapping sets.

### Quantitative real-time reverse transcriptase PCR (qRT-PCR)

We confirmed gene expression patterns noted by microarray by qRT-PCR using a 2-step method. Total RNA for template was purified from tumor and cell line samples independent from those used for microarray; 12–18 tumor samples and 12 cell line samples were used in each qRT-PCR assay. First strand cDNA was synthesized using the Superscript III kit (Invitrogen). We performed qRT-PCR reactions on the BioRad iCycler and IQ SYBR Green Master Mix (170–8882; BioRad). All reactions were performed in duplicate. All primer pairs were designed to span at least 1 intron-exon boundaries, except for VIP, which has no introns (Table S3). Each primer pair was validated by RT-PCR and the products were cloned and sequenced to confirm specificity. Cloned PCR products were then used as copy number standards for qRT-PCR. Each primer pair yielded measured Ct values in control reactions that varied linearly with the log of copy number over at least 6 orders of magnitude. We normalized all values to the measured expression of beta actin (BA). Fold change was computed by comparing the median copy number for each group.

### Immunocytochemistry and Western blots

All immunocytochemistry was performed on paraffin embedded material from pathology archives of The Memorial Sloan-Kettering Cancer Center (MSKCC). Antigen retrieval and staining techniques were as previously described [4]. Cultured cells were not subjected to antigen retrieval. For Western blots, SY-Gli and SY-GFP cells were prepared in triplicate wells, and lysed in situ, then subjected to SDS-PAGE, and developed using chemiluminescence. Sources of primary antibodies and concentrations used are as listed ( Table S3).

## Supporting Information

Table S1 Genes differentially expressed in GN compared to NB. Mean values are computed from log2 transformed expression values. The difference in log2 transformed means is therefore equal to log2 (fold change).
(4.10 MB DOC)

Table S2 Genes differentially expressed in SY5Y cells tranduced with Gli1 and GFP or GFP only. Mean values are computed from log2 transformed expression values. The difference in log2 transformed means is therefore equal to log2 (fold change).
(0.33 MB DOC)

Table S3 Primers and antibodies used
(0.03 MB DOC)

## References

[1] Park JR, Eggert A, Caron H (2008). Neuroblastoma: biology, prognosis, and treatment.. Pediatr Clin North Am.

[2] Griffin ME, Bolande RP (1969). Familial neuroblastoma with regression and maturation to ganglioneurofibroma.. Pediatrics.

[3] Matthay KK, Villablanca JG, Seeger RC, Stram DO, Harris RE (1999). Treatment of high-risk neuroblastoma with intensive chemotherapy, radiotherapy, autologous bone marrow transplantation, and 13-cis-retinoic acid. Children's Cancer Group.. N Engl J Med.

[4] Gershon TR, Oppenheimer O, Chin SS, Gerald WL (2005). Temporally regulated neural crest transcription factors distinguish neuroectodermal tumors of varying malignancy and differentiation.. Neoplasia.

[5] Kenney AM, Rowitch DH (2000). Sonic hedgehog promotes G(1) cyclin expression and sustained cell cycle progression in mammalian neuronal precursors.. Mol Cell Biol.

[6] Palma V, Lim DA, Dahmane N, Sanchez P, Brionne TC (2005). Sonic hedgehog controls stem cell behavior in the postnatal and adult brain.. Development.

[7] Raffel C, Jenkins RB, Frederick L, Hebrink D, Alderete B (1997). Sporadic medulloblastomas contain PTCH mutations.. Cancer Res.

[8] Goodrich LV, Milenkovic L, Higgins KM, Scott MP (1997). Altered neural cell fates and medulloblastoma in mouse patched mutants.. Science.

[9] Hallahan AR, Pritchard JI, Hansen S, Benson M, Stoeck J (2004). The SmoA1 mouse model reveals that notch signaling is critical for the growth and survival of sonic hedgehog-induced medulloblastomas.. Cancer Res.

[10] Jeong J, Mao J, Tenzen T, Kottmann AH, McMahon AP (2004). Hedgehog signaling in the neural crest cells regulates the patterning and growth of facial primordia.. Genes Dev.

[11] Fu M, Lui VC, Sham MH, Pachnis V, Tam PK (2004). Sonic hedgehog regulates the proliferation, differentiation, and migration of enteric neural crest cells in gut.. J Cell Biol.

[12] Chamberlain CE, Jeong J, Guo C, Allen BL, McMahon AP (2008). Notochord-derived Shh concentrates in close association with the apically positioned basal body in neural target cells and forms a dynamic gradient during neural patterning.. Development.

[13] Sukegawa A, Narita T, Kameda T, Saitoh K, Nohno T (2000). The concentric structure of the developing gut is regulated by Sonic hedgehog derived from endodermal epithelium.. Development.

[14] Smyth GK (2004). Linear models and empirical bayes methods for assessing differential expression in microarray experiments.. Stat Appl Genet Mol Biol.

[15] Tischler AS (2000). Divergent differentiation in neuroendocrine tumors of the adrenal gland.. Semin Diagn Pathol.

[16] Barrallo-Gimeno A, Holzschuh J, Driever W, Knapik EW (2004). Neural crest survival and differentiation in zebrafish depends on mont blanc/tfap2a gene function.. Development.

[17] Hong SJ, Lardaro T, Oh MS, Huh Y, Ding Y (2008). Regulation of the noradrenaline neurotransmitter phenotype by the transcription factor AP-2beta.. J Biol Chem.

[18] Kirchgessner AL, Dodd J, Gershon MD (1988). Markers shared between dorsal root and enteric ganglia.. J Comp Neurol.

[19] Zigmond RE, Sun Y (1997). Regulation of neuropeptide expression in sympathetic neurons. Paracrine and retrograde influences.. Ann N Y Acad Sci.

[20] Lindh B, Lundberg JM, Hokfelt T (1989). NPY-, galanin-, VIP/PHI-, CGRP- and substance P-immunoreactive neuronal subpopulations in cat autonomic and sensory ganglia and their projections.. Cell Tissue Res.

[21] Jessen KR, Thorpe R, Mirsky R (1984). Molecular identity, distribution and heterogeneity of glial fibrillary acidic protein: an immunoblotting and immunohistochemical study of Schwann cells, satellite cells, enteric glia and astrocytes.. J Neurocytol.

[22] Elfvin LG, Bjorklund H, Dahl D, Seiger A (1987). Neurofilament-like and glial fibrillary acidic protein-like immunoreactivities in rat and guinea-pig sympathetic ganglia in situ and after perturbation.. Cell Tissue Res.

[23] Hosoda K, Hammer RE, Richardson JA, Baynash AG, Cheung JC (1994). Targeted and natural (piebald-lethal) mutations of endothelin-B receptor gene produce megacolon associated with spotted coat color in mice.. Cell.

[24] Puffenberger EG, Hosoda K, Washington SS, Nakao K, deWit D (1994). A missense mutation of the endothelin-B receptor gene in multigenic Hirschsprung's disease.. Cell.

[25] Lee J, Platt KA, Censullo P, Ruiz i Altaba A (1997). Gli1 is a target of Sonic hedgehog that induces ventral neural tube development.. Development.

[26] Lipinski RJ, Cook CH, Barnett DH, Gipp JJ, Peterson RE (2005). Sonic hedgehog signaling regulates the expression of insulin-like growth factor binding protein-6 during fetal prostate development.. Dev Dyn.

[27] Oliver TG, Grasfeder LL, Carroll AL, Kaiser C, Gillingham CL (2003). Transcriptional profiling of the Sonic hedgehog response: a critical role for N-myc in proliferation of neuronal precursors.. Proc Natl Acad Sci U S A.

[28] Ingram WJ, Wicking CA, Grimmond SM, Forrest AR, Wainwright BJ (2002). Novel genes regulated by Sonic Hedgehog in pluripotent mesenchymal cells.. Oncogene.

[29] Yoon JW, Kita Y, Frank DJ, Majewski RR, Konicek BA (2002). Gene expression profiling leads to identification of GLI1-binding elements in target genes and a role for multiple downstream pathways in GLI1-induced cell transformation.. J Biol Chem.

[30] Cariboni A, Pimpinelli F, Colamarino S, Zaninetti R, Piccolella M (2004). The product of X-linked Kallmann's syndrome gene (KAL1) affects the migratory activity of gonadotropin-releasing hormone (GnRH)-producing neurons.. Hum Mol Genet.

[31] Whitlock KE, Wolf CD, Boyce ML (2003). Gonadotropin-releasing hormone (GnRH) cells arise from cranial neural crest and adenohypophyseal regions of the neural plate in the zebrafish, Danio rerio.. Dev Biol.

[32] Han J, Ishii M, Bringas P, Maas RL, Maxson RE (2007). Concerted action of Msx1 and Msx2 in regulating cranial neural crest cell differentiation during frontal bone development.. Mech Dev.

[33] Abe M, Ruest LB, Clouthier DE (2007). Fate of cranial neural crest cells during craniofacial development in endothelin-A receptor-deficient mice.. Int J Dev Biol.

[34] Nair S, Li W, Cornell R, Schilling TF (2007). Requirements for Endothelin type-A receptors and Endothelin-1 signaling in the facial ectoderm for the patterning of skeletogenic neural crest cells in zebrafish.. Development.

[35] Edery P, Lyonnet S, Mulligan LM, Pelet A, Dow E (1994). Mutations of the RET proto-oncogene in Hirschsprung's disease.. Nature.

[36] Rossi J, Herzig KH, Voikar V, Hiltunen PH, Segerstrale M (2003). Alimentary tract innervation deficits and dysfunction in mice lacking GDNF family receptor alpha2.. J Clin Invest.

[37] Oliver TG, Grasfeder LL, Carroll AL, Kaiser C, Gillingham CL (2003). Transcriptional profiling of the Sonic hedgehog response: a critical role for N-myc in proliferation of neuronal precursors.. Proceedings of the National Academy of Sciences of the United States of America.

[38] Oppenheimer O, Cheung N-K, Gerald WL (2007). The RET oncogene is a critical component of transcriptional programs associated with retinoic acid-induced differentiation in neuroblastoma.. Molecular cancer therapeutics.

[39] Quak SH, Prabhakaran K, Kwok R, O'Reilly AP (1988). Vasoactive intestinal peptide secreting tumours in children: a case report with literature review.. Aust Paediatr J.

[40] Shkumatava A, Neumann CJ (2005). Shh directs cell-cycle exit by activating p57Kip2 in the zebrafish retina.. EMBO Rep.

[41] Galvin KE, Ye H, Erstad DJ, Feddersen R, Wetmore C (2008). Gli1 induces G2/M arrest and apoptosis in hippocampal but not tumor-derived neural stem cells.. Stem Cells.

[42] Mao J, Ligon KL, Rakhlin EY, Thayer SP, Bronson RT (2006). A novel somatic mouse model to survey tumorigenic potential applied to the Hedgehog pathway.. Cancer Res.

[43] Weiss WA, Aldape K, Mohapatra G, Feuerstein BG, Bishop JM (1997). Targeted expression of MYCN causes neuroblastoma in transgenic mice.. EMBO J.

[44] Sharghi-Namini S, Turmaine M, Meier C, Sahni V, Umehara F (2006). The structural and functional integrity of peripheral nerves depends on the glial-derived signal desert hedgehog.. J Neurosci.

[45] Kwiatkowski JL, Rutkowski JL, Yamashiro DJ, Tennekoon GI, Brodeur GM (1998). Schwann cell-conditioned medium promotes neuroblastoma survival and differentiation.. Cancer Res.

[46] Liu S, Tian Y, Chlenski A, Yang Q, Salwen HR (2005). ‘Cross-talk’ between Schwannian stroma and neuroblasts promotes neuroblastoma tumor differentiation and inhibits angiogenesis.. Cancer Lett.

[47] Parmantier E, Lynn B, Lawson D, Turmaine M, Namini SS (1999). Schwann cell-derived Desert hedgehog controls the development of peripheral nerve sheaths.. Neuron.

[48] Alaminos M, Mora J, Cheung NK, Smith A, Qin J (2003). Genome-wide analysis of gene expression associated with MYCN in human neuroblastoma.. Cancer Res.

[49] Irizarry RA, Hobbs B, Collin F, Beazer-Barclay YD, Antonellis KJ (2003). Exploration, normalization, and summaries of high density oligonucleotide array probe level data.. Biostatistics.

