# Correction: Enteric Neural Crest Differentiation in Ganglioneuromas Implicates Hedgehog Signaling in Peripheral Neuroblastic Tumor Pathogenesis

**DOI:** 10.1371/journal.pone.0103881

**Published:** 2014-07-25

**Authors:** The PLOS ONE Staff

The second author’s name is spelled incorrectly. The correct name is: Ashton Shiraz. The correct citation is: Gershon TR, Shiraz A, Qin L-X, Gerald WL, Kenney AM, et al. (2009) Enteric Neural Crest Differentiation in Ganglioneuromas Implicates Hedgehog Signaling in Peripheral Neuroblastic Tumor Pathogenesis. PLoS ONE 4(10): e7491. doi:10.1371/journal.pone.0007491
