# Supplementary material for: Enteric Neural Crest Differentiation in Ganglioneuromas Implicates Hedgehog Signaling in Peripheral Neuroblastic Tumor Pathogenesis
Source: PLoS One. 2009 Oct 16;4(10):e7491. doi: 10.1371/journal.pone.0007491 (PMC2759000; doi:10.1371/journal.pone.0007491)
Supplement: Table S1 — Genes differentially expressed in GN compared to NB. Mean values are computed from log2 transformed expression values. The difference in log2 transformed means is therefore equal to log2 (fold change). (4.10 MB DOC) [file pone.0007491.s001.doc]

**Supplemental Table 1** Genes differentially expressed in GN compared to NB. Mean values are computed from log2 transformed expression values. The difference in log2 transformed means is therefore equal to log2 (fold change).

| ProbeSetID | GeneSymbol | Pvalue: GN -NB | Diff: GN - NB | NB-mean | GN mean |
| --- | --- | --- | --- | --- | --- |
| 35717_at | ABCA8 | 3.93E-49 | 4.85 | 3.07 | 7.92 |
| 40516_at | AHR | 2.89E-47 | 3.9 | 2.97 | 6.86 |
| 39654_at | ASPA | 4.18E-47 | 3.28 | 3.45 | 6.73 |
| 33387_at | GAS7 | 2.69E-45 | 4.31 | 3.83 | 8.14 |
| 39452_s_at | SPTBN1 | 1.66E-43 | 3.22 | 5.33 | 8.55 |
| 36018_at | SOX10 | 1.67E-43 | 3.35 | 6.72 | 10.07 |
| 32109_at | FXYD1 | 5.64E-43 | 4.27 | 7.05 | 11.32 |
| 34693_at | ST6GALNAC2 | 2.30E-42 | 3 | 4.99 | 7.99 |
| 37251_s_at | GPM6B | 6.59E-42 | 5.04 | 4.34 | 9.38 |
| 33118_at | SEMA3B | 2.06E-41 | 4.2 | 5.69 | 9.89 |
| 31790_at | STARD13 | 4.03E-41 | 2.93 | 4.03 | 6.96 |
| 182_at | ITPR3 | 1.03E-40 | 2.52 | 5.38 | 7.9 |
| 35817_at | MBP | 1.82E-40 | 4.1 | 4 | 8.1 |
| 39940_at | RPESP | 4.58E-40 | 2.07 | 4.39 | 6.46 |
| 39760_at | QKI | 1.73E-39 | 3.92 | 4.44 | 8.36 |
| 34922_at | CDH19 | 1.46E-38 | 5.33 | 3.78 | 9.11 |
| 34180_at | ARHGEF10 | 1.66E-38 | 2.14 | 6.86 | 9 |
| 38109_at | EGFL8 | 1.95E-38 | 2.89 | 6.4 | 9.29 |
| 41158_at | PLP1 | 3.95E-38 | 6.07 | 4.69 | 10.76 |
| 39271_at | MIA | 3.96E-38 | 2.91 | 5.92 | 8.83 |
| 37343_at | ITPR3 | 9.32E-38 | 2.57 | 5.9 | 8.47 |
| 36989_at | DAG1 | 2.02E-37 | 2.03 | 6.83 | 8.86 |
| 39631_at | EMP2 | 1.95E-36 | 2.25 | 5.13 | 7.38 |
| 406_at | ITGB4 | 6.95E-36 | 2.79 | 6.16 | 8.95 |
| 36681_at | APOD | 1.05E-35 | 6.86 | 5.35 | 12.21 |
| 34303_at | C10orf56 | 1.71E-35 | 3.04 | 6.9 | 9.94 |
| 35331_at | CTNNAL1 | 1.28E-34 | 3.17 | 5.89 | 9.06 |
| 1364_at | PTPRZ1 | 1.42E-34 | 2.45 | 2.77 | 5.22 |
| 34377_at | ATP1A2 | 1.60E-34 | 2.29 | 3.82 | 6.11 |
| 32239_at | MATN2 | 2.66E-33 | 3.08 | 3.15 | 6.22 |
| 32787_at | ERBB3 | 1.22E-31 | 3.79 | 4.38 | 8.17 |
| 36455_at | COL9A3 | 1.39E-31 | 2.34 | 4.33 | 6.67 |
| 37403_at | ANXA1 | 1.93E-31 | 3.26 | 4.31 | 7.57 |
| 33137_at | LTBP4 | 2.54E-31 | 1.99 | 7.88 | 9.87 |
| 2089_s_at | ERBB3 | 2.88E-31 | 1.97 | 5.11 | 7.08 |
| 1585_at | ERBB3 | 2.99E-31 | 4.06 | 3.79 | 7.85 |
| 32242_at | CRYAB | 7.68E-31 | 3.89 | 5.64 | 9.54 |
| 38051_at | MAL | 1.60E-30 | 2.21 | 7.17 | 9.38 |
| 34198_at | PTPN13 | 2.19E-30 | 2.17 | 3.46 | 5.64 |
| 34388_at | COL14A1 | 4.96E-30 | 2.66 | 3.62 | 6.28 |
| 40455_at | ENDOD1 | 5.51E-30 | 2.81 | 5.02 | 7.84 |
| 235_at | S100B | 2.53E-29 | 1.83 | 5.53 | 7.36 |
| 41871_at | PDPN | 7.93E-29 | 1.79 | 5.82 | 7.61 |
| 36650_at | CCND2 | 9.55E-29 | 2.61 | 4.42 | 7.03 |
| 39260_at | SLC16A4 | 1.26E-28 | 3.16 | 3.02 | 6.18 |
| 32243_g_at | CRYAB | 1.78E-28 | 3.98 | 5.97 | 9.95 |
| 35752_s_at | PROS1 | 1.82E-28 | 2.74 | 4.71 | 7.45 |
| 1629_s_at | --- | 2.74E-28 | 1.55 | 3.98 | 5.54 |
| 35704_at | HRASLS3 | 3.50E-28 | 2.04 | 4.98 | 7.02 |
| 34714_at | SAMHD1 | 4.36E-28 | 1.95 | 3.63 | 5.59 |
| 38972_at | KCTD12 | 5.57E-28 | 2.83 | 4.49 | 7.32 |
| 33891_at | CLIC4 | 8.31E-28 | 2.79 | 7.08 | 9.87 |
| 32585_at | EPB41L2 | 8.49E-28 | 3.07 | 4.62 | 7.69 |
| 37070_at | MPZ | 9.13E-28 | 1.97 | 6.25 | 8.22 |
| 39338_at | S100A10 | 1.66E-27 | 3.2 | 6.3 | 9.5 |
| 37821_at | BCAS1 | 1.97E-27 | 1.61 | 4.24 | 5.85 |
| 36014_at | GPR126 | 2.18E-27 | 2.71 | 3.8 | 6.51 |
| 36543_at | F3 | 2.22E-27 | 3.3 | 2.95 | 6.25 |
| 35390_at | ABCA6 | 4.40E-27 | 1.55 | 2.66 | 4.21 |
| 33303_at | SSPN | 9.30E-27 | 1.15 | 3.43 | 4.58 |
| 40488_at | DMD | 1.05E-26 | 3.35 | 4.39 | 7.74 |
| 39351_at | CD59 | 2.41E-26 | 2.4 | 6.37 | 8.77 |
| 37027_at | AHNAK | 7.36E-26 | 3.54 | 8.25 | 11.78 |
| 32226_at | MAP4 | 7.53E-26 | 1.65 | 7.74 | 9.39 |
| 33700_at | SPRY2 | 7.64E-26 | 1.93 | 4.88 | 6.82 |
| 32168_s_at | RCAN1 | 8.24E-26 | 2.13 | 4.44 | 6.58 |
| 38700_at | CSRP1 | 8.24E-26 | 2.13 | 7.79 | 9.91 |
| 35605_at | ANGPTL7 | 1.14E-25 | 1.96 | 4.01 | 5.97 |
| 37543_at | ARHGEF6 | 1.33E-25 | 1.67 | 5.53 | 7.19 |
| 33254_at | EVI5 | 1.50E-25 | 1.38 | 3.73 | 5.11 |
| 37842_at | MDFIC | 2.58E-25 | 1.69 | 3.78 | 5.47 |
| 36917_at | LAMA2 | 2.77E-25 | 3.24 | 5.32 | 8.56 |
| 38037_at | HBEGF | 3.80E-25 | 1.21 | 5.59 | 6.8 |
| 243_g_at | MAP4 | 4.84E-25 | 1.89 | 7.05 | 8.94 |
| 1815_g_at | TGFBR2 | 1.48E-24 | 3.16 | 3.4 | 6.56 |
| 1198_at | EDNRB | 2.02E-24 | 1.35 | 2.64 | 4 |
| 1736_at | IGFBP6 | 2.10E-24 | 2.4 | 5.22 | 7.62 |
| 37539_at | RGL1 | 2.28E-24 | 1.44 | 6.23 | 7.67 |
| 33218_at | ERBB2 | 2.86E-24 | 1.27 | 6.36 | 7.64 |
| 41098_at | DAAM2 | 3.12E-24 | 1.34 | 6.42 | 7.76 |
| 38338_at | RRAS | 3.84E-24 | 1.32 | 7.33 | 8.65 |
| 35367_at | LGALS3 | 4.40E-24 | 3.34 | 4.88 | 8.22 |
| 31855_at | SRPX | 4.84E-24 | 1.61 | 5.94 | 7.55 |
| 32610_at | PDLIM4 | 7.06E-24 | 1.79 | 6.96 | 8.75 |
| 37863_at | EGR2 | 9.06E-24 | 2.3 | 3.09 | 5.38 |
| 40782_at | DHRS3 | 9.34E-24 | 2.36 | 6.71 | 9.07 |
| 40504_at | PON2 | 1.88E-23 | 2.22 | 3.9 | 6.11 |
| 1673_at | NGFR | 3.75E-23 | 2.51 | 6.88 | 9.39 |
| 33905_at | MBD2 | 6.44E-23 | 1.48 | 4.95 | 6.43 |
| 37530_s_at | RELN | 6.64E-23 | 2.95 | 4.88 | 7.84 |
| 755_at | ITPR1 | 6.79E-23 | 2.05 | 5.16 | 7.21 |
| 37623_at | NR4A2 | 8.31E-23 | 3.51 | 3.72 | 7.23 |
| 40834_at | PDZD2 | 8.89E-23 | 1.99 | 3.35 | 5.33 |
| 35389_s_at | ABCA6 | 9.10E-23 | 1.46 | 2.89 | 4.35 |
| 33410_at | ITGA6 | 1.03E-22 | 2.3 | 5.34 | 7.65 |
| 40423_at | EHBP1 | 1.03E-22 | 1.99 | 7.32 | 9.31 |
| 39623_at | NDP | 1.11E-22 | 1.03 | 3.8 | 4.83 |
| 39170_at | CD59 | 1.21E-22 | 1.99 | 7.68 | 9.66 |
| 41870_at | PDPN | 1.55E-22 | 1.33 | 5.54 | 6.87 |
| 34887_at | RDX | 1.66E-22 | 2.11 | 5.09 | 7.2 |
| 36894_at | CBX7 | 2.74E-22 | 1.75 | 7 | 8.75 |
| 36396_at | --- | 3.09E-22 | 1.46 | 4.31 | 5.77 |
| 39214_at | PLXNB3 | 4.62E-22 | 1.11 | 5.21 | 6.33 |
| 37215_at | PYGL | 6.23E-22 | 2.54 | 3.62 | 6.16 |
| 33900_at | FSTL3 | 6.38E-22 | 1.63 | 7.32 | 8.95 |
| 40375_at | EGR3 | 6.40E-22 | 2.69 | 4.38 | 7.08 |
| 41688_at | PLLP | 7.79E-22 | 1.22 | 5.86 | 7.08 |
| 33452_at | PLAT | 9.47E-22 | 1.9 | 6.01 | 7.91 |
| 37762_at | EMP1 | 9.78E-22 | 2.41 | 3.95 | 6.35 |
| 1814_at | TGFBR2 | 1.19E-21 | 3.18 | 6.25 | 9.43 |
| 889_at | ITGB8 | 1.26E-21 | 1.2 | 3.29 | 4.49 |
| 38433_at | AXL | 1.88E-21 | 2.08 | 5.17 | 7.26 |
| 40570_at | FOXO1 | 2.09E-21 | 2.58 | 5.94 | 8.52 |
| 32612_at | GSN | 2.24E-21 | 3.41 | 5.77 | 9.18 |
| 34800_at | LRIG1 | 2.92E-21 | 1.87 | 4.51 | 6.38 |
| 33411_g_at | ITGA6 | 2.97E-21 | 1.88 | 3.84 | 5.72 |
| 1278_at | --- | 3.13E-21 | 2.31 | 4.73 | 7.04 |
| 32083_at | GPR137B | 3.67E-21 | 1.18 | 3.26 | 4.43 |
| 33876_at | WWTR1 | 4.17E-21 | 3.49 | 4.88 | 8.37 |
| 35219_at | LOC727882 | 4.22E-21 | 1.05 | 8.64 | 9.69 |
| 36452_at | SYNPO | 4.32E-21 | 1.32 | 9.02 | 10.34 |
| 38033_at | 10-Sep | 5.82E-21 | 2.57 | 5.55 | 8.12 |
| 242_at | MAP4 | 6.32E-21 | 1.85 | 6.63 | 8.48 |
| 41634_at | KIAA0256 | 6.51E-21 | 1.88 | 6.38 | 8.26 |
| 849_g_at | TRAF1 | 6.69E-21 | 1.21 | 5.81 | 7.03 |
| 37312_at | SERTAD2 | 7.51E-21 | 1.34 | 5.63 | 6.97 |
| 33462_at | P2RY14 | 7.65E-21 | 1.94 | 3.24 | 5.17 |
| 33236_at | RARRES3 | 7.74E-21 | 1.47 | 5.41 | 6.88 |
| 32664_at | RNASE4 | 7.80E-21 | 1.99 | 5.15 | 7.14 |
| 31895_at | BACH1 | 8.41E-21 | 1.68 | 5.93 | 7.61 |
| 41666_at | HSPA12A | 9.13E-21 | 2.07 | 5.7 | 7.77 |
| 38188_s_at | MAN2A2 | 9.73E-21 | 1.25 | 7.06 | 8.3 |
| 37344_at | HLA-DMA | 1.04E-20 | 2.26 | 7.24 | 9.5 |
| 32527_at | C10orf116 | 1.12E-20 | 2.02 | 5.36 | 7.38 |
| 38409_at | SSFA2 | 1.38E-20 | 1.11 | 4.12 | 5.23 |
| 35669_at | COBL | 1.52E-20 | 3.02 | 4.51 | 7.52 |
| 36834_at | MOXD1 | 1.56E-20 | 1.65 | 5.02 | 6.66 |
| 1563_s_at | TNFRSF1A | 1.72E-20 | 1.57 | 7.27 | 8.85 |
| 33850_at | MAP4 | 1.81E-20 | 1.19 | 7.79 | 8.98 |
| 37958_at | TMEM47 | 2.00E-20 | 2.62 | 4.38 | 7 |
| 34651_at | COMT | 2.17E-20 | 1.04 | 7.52 | 8.56 |
| 32533_s_at | VAMP5 | 2.97E-20 | 1.49 | 6.42 | 7.91 |
| 38402_at | LAMP2 | 3.60E-20 | 1.74 | 3.81 | 5.55 |
| 41266_at | ITGA6 | 3.71E-20 | 3.38 | 7.25 | 10.64 |
| 36369_at | PTRF | 6.63E-20 | 1.4 | 5.45 | 6.85 |
| 39842_at | CRLF1 | 6.69E-20 | 1.86 | 6.5 | 8.36 |
| 41274_at | DKFZp667G2110 | 6.93E-20 | 1 | 5.5 | 6.51 |
| 35644_at | HEPH | 7.23E-20 | 1.12 | 3.81 | 4.94 |
| 36577_at | PLEKHC1 | 8.40E-20 | 1.94 | 6.8 | 8.74 |
| 40511_at | GATA3 | 8.63E-20 | -3.59 | 8.73 | 5.14 |
| 37774_at | 6-Sep | 1.14E-19 | -2.32 | 8.25 | 5.92 |
| 41505_r_at | MAF | 1.23E-19 | 1.95 | 3.79 | 5.74 |
| 40790_at | BHLHB2 | 1.24E-19 | 2.55 | 6.79 | 9.34 |
| 32778_at | ITPR1 | 1.55E-19 | 1.66 | 3.32 | 4.97 |
| 31684_at | ANXA2P1 | 1.57E-19 | 1.31 | 6.54 | 7.85 |
| 38164_at | RPGR | 1.62E-19 | 1.54 | 4.33 | 5.87 |
| 41257_at | CAST | 1.70E-19 | 1.48 | 3.26 | 4.75 |
| 36637_at | ANXA11 | 1.72E-19 | 1.35 | 5.87 | 7.22 |
| 34193_at | CHL1 | 1.78E-19 | 3.64 | 5.31 | 8.95 |
| 37560_at | SNED1 | 2.40E-19 | 1.1 | 4.74 | 5.84 |
| 1761_at | PDGFRL | 2.77E-19 | 1.76 | 3.38 | 5.14 |
| 37775_at | 6-Sep | 2.80E-19 | -2.34 | 8.2 | 5.85 |
| 36488_at | MEGF9 | 2.81E-19 | 1.58 | 6.07 | 7.65 |
| 39341_at | TRIP6 | 3.01E-19 | 0.96 | 7.13 | 8.09 |
| 34320_at | PTRF | 3.10E-19 | 1.87 | 8.96 | 10.83 |
| 36686_at | ALDH1A3 | 3.44E-19 | 2.26 | 4.03 | 6.29 |
| 40272_at | CRMP1 | 4.23E-19 | -2.88 | 9.27 | 6.39 |
| 266_s_at | CD24 | 4.28E-19 | -2.67 | 8 | 5.32 |
| 32666_at | CXCL12 | 4.81E-19 | 2.3 | 4.91 | 7.21 |
| 36491_at | TMSL8 | 5.76E-19 | -3.51 | 8.48 | 4.97 |
| 39693_at | TMEM109 | 5.78E-19 | 1.04 | 6.98 | 8.01 |
| 36363_at | UGT8 | 6.37E-19 | 1.67 | 3.3 | 4.97 |
| 41209_at | LPL | 6.47E-19 | 2.23 | 5.68 | 7.91 |
| 37631_at | MYO1E | 7.56E-19 | 1.03 | 5.27 | 6.29 |
| 38021_at | PLEC1 | 8.23E-19 | 1.16 | 8.44 | 9.61 |
| 39057_at | KLC1 | 8.98E-19 | 1.58 | 7.52 | 9.09 |
| 36843_at | SIPA1 | 1.11E-18 | 0.91 | 6.55 | 7.46 |
| 39521_at | SLC12A4 | 1.24E-18 | 0.85 | 6.21 | 7.05 |
| 1395_at | RHOC | 1.29E-18 | 1.59 | 8.02 | 9.61 |
| 34306_at | MBNL1 | 1.32E-18 | 1.57 | 4.59 | 6.16 |
| 39709_at | SEPW1 | 1.48E-18 | 1.03 | 8.84 | 9.87 |
| 41137_at | PPP1R12B | 1.56E-18 | 1.12 | 5.97 | 7.09 |
| 34675_at | STON1 | 1.86E-18 | 0.7 | 4.69 | 5.39 |
| 40202_at | KLF9 | 2.18E-18 | 2.34 | 6.57 | 8.91 |
| 36690_at | NR3C1 | 2.26E-18 | 1.24 | 5.16 | 6.4 |
| 541_g_at | HSPB2 | 2.34E-18 | 0.78 | 7.03 | 7.81 |
| 1372_at | TNFAIP6 | 2.54E-18 | 1.15 | 4.3 | 5.45 |
| 39071_at | ITGAV | 2.63E-18 | 1.48 | 4.25 | 5.73 |
| 40841_at | TACC1 | 2.75E-18 | 1.73 | 8.02 | 9.75 |
| 1456_s_at | IFI16 | 2.84E-18 | 2.43 | 5.02 | 7.45 |
| 1035_g_at | TIMP3 | 2.86E-18 | 1.61 | 7.07 | 8.68 |
| 35681_r_at | ZEB2 | 3.20E-18 | 2.44 | 3.51 | 5.96 |
| 37676_at | PDE8A | 3.20E-18 | 1.29 | 6.97 | 8.26 |
| 37142_at | GFRA1 | 3.36E-18 | 0.99 | 2.53 | 3.52 |
| 1879_at | RRAS | 3.43E-18 | 1.48 | 5.45 | 6.93 |
| 41489_at | TLE1 | 3.51E-18 | 1.41 | 5.77 | 7.18 |
| 34752_at | NEK7 | 4.17E-18 | 1.51 | 4.95 | 6.46 |
| 37638_at | DOCK1 | 4.42E-18 | 1.11 | 5.24 | 6.35 |
| 32700_at | GBP2 | 4.52E-18 | 1.31 | 5.75 | 7.06 |
| 41153_f_at | CTNNA1 | 5.60E-18 | 1.51 | 7.69 | 9.2 |
| 32135_at | SREBF1 | 5.76E-18 | 0.97 | 8.47 | 9.44 |
| 35282_r_at | CD81 | 6.21E-18 | 1.19 | 10.07 | 11.26 |
| 32779_s_at | ITPR1 | 6.49E-18 | 0.97 | 5.24 | 6.21 |
| 36892_at | ITGA7 | 7.12E-18 | 1.9 | 7.65 | 9.55 |
| 39590_at | APBA2 | 7.23E-18 | -2.33 | 8.16 | 5.83 |
| 40953_at | CNN3 | 7.25E-18 | 2.02 | 7.01 | 9.03 |
| 39397_at | NR2F2 | 8.04E-18 | 2.73 | 5.98 | 8.71 |
| 38406_f_at | PTGDS | 8.15E-18 | 2 | 8.92 | 10.92 |
| 38584_at | IFIT3 | 8.19E-18 | 1.23 | 5.63 | 6.86 |
| 37459_at | COL8A1 | 8.38E-18 | 1.39 | 3.46 | 4.84 |
| 36825_at | TRIM22 | 8.51E-18 | 1.12 | 3.96 | 5.07 |
| 34886_at | RDX | 8.69E-18 | 1.42 | 6.02 | 7.44 |
| 38079_at | GNG12 | 8.75E-18 | 2.42 | 5.22 | 7.65 |
| 37015_at | ALDH1A1 | 1.05E-17 | 3.79 | 4.41 | 8.2 |
| 34246_at | C6orf145 | 1.08E-17 | 1.18 | 5.78 | 6.96 |
| 1802_s_at | ERBB2 | 1.11E-17 | 1.27 | 6.03 | 7.31 |
| 37745_s_at | ST5 | 1.13E-17 | 0.79 | 6.61 | 7.4 |
| 33813_at | TNFRSF1B | 1.17E-17 | 1.02 | 8.09 | 9.11 |
| 39409_at | C1R | 1.22E-17 | 2.8 | 6.84 | 9.64 |
| 32094_at | CHST3 | 1.26E-17 | 0.74 | 6.43 | 7.17 |
| 37043_at | ID3 | 1.33E-17 | 1.85 | 7.98 | 9.83 |
| 33295_at | DARC | 1.45E-17 | 1.75 | 6.56 | 8.31 |
| 39710_at | C5orf13 | 1.60E-17 | -2.44 | 9.91 | 7.47 |
| 1648_at | OSMR | 1.63E-17 | 1.07 | 3.04 | 4.11 |
| 757_at | ANXA2 | 1.90E-17 | 1.74 | 5.32 | 7.06 |
| 853_at | NFE2L2 | 1.92E-17 | 1.59 | 5.15 | 6.73 |
| 41449_at | SGCE | 2.29E-17 | 1.45 | 7.3 | 8.75 |
| 39277_at | --- | 2.48E-17 | 0.89 | 5.34 | 6.23 |
| 38488_s_at | IL15 | 2.57E-17 | 1.24 | 3.18 | 4.42 |
| 32035_at | HLA-DRB4 | 2.57E-17 | 2.6 | 5.53 | 8.14 |
| 1048_at | RXRG | 2.63E-17 | 1.25 | 4.62 | 5.87 |
| 36773_f_at | HLA-DQB1 | 3.02E-17 | 2.09 | 6.31 | 8.4 |
| 36812_at | BCAR3 | 3.18E-17 | 0.66 | 4.38 | 5.04 |
| 33803_at | THBD | 3.29E-17 | 1.51 | 4.75 | 6.26 |
| 216_at | PTGDS | 3.51E-17 | 1.94 | 9.02 | 10.96 |
| 38643_at | FAM114A1 | 3.59E-17 | 1.81 | 5.89 | 7.7 |
| 41589_at | KCNQ2 | 3.70E-17 | -2.32 | 7.78 | 5.45 |
| 36878_f_at | HLA-DQB1 | 3.99E-17 | 2.09 | 6.19 | 8.28 |
| 36933_at | NDRG1 | 4.17E-17 | 1.77 | 7.8 | 9.57 |
| 40071_at | CYP1B1 | 4.32E-17 | 2.98 | 4.09 | 7.07 |
| 35776_at | ITSN1 | 4.47E-17 | 0.81 | 7.27 | 8.08 |
| 41504_s_at | MAF | 5.18E-17 | 2.72 | 4.89 | 7.61 |
| 38243_at | NCAM1 | 5.97E-17 | 0.8 | 3.49 | 4.29 |
| 39182_at | EMP3 | 6.24E-17 | 1.74 | 6.11 | 7.85 |
| 706_at | --- | 6.58E-17 | 1.19 | 3.99 | 5.18 |
| 37270_at | ATP1B2 | 6.82E-17 | 0.91 | 7.09 | 8 |
| 32845_at | HSPG2 | 7.00E-17 | 1.81 | 8.21 | 10.01 |
| 38980_at | MAP3K7IP2 | 7.11E-17 | 1.39 | 5.81 | 7.19 |
| 1034_at | TIMP3 | 7.42E-17 | 1.43 | 7.01 | 8.44 |
| 35358_at | TENC1 | 7.50E-17 | 1.03 | 6.67 | 7.71 |
| 39990_at | ISL1 | 8.08E-17 | -3.04 | 9.13 | 6.09 |
| 1593_at | FGF2 | 8.74E-17 | 0.65 | 2.6 | 3.25 |
| 870_f_at | MT3 | 8.78E-17 | 1.35 | 7.93 | 9.28 |
| 38280_s_at | NTRK2 | 9.08E-17 | 0.87 | 2.83 | 3.7 |
| 37194_at | GATA2 | 1.01E-16 | -2.68 | 8.37 | 5.69 |
| 34678_at | FER1L3 | 1.01E-16 | 2.01 | 5.38 | 7.39 |
| 411_i_at | IFITM2 | 1.06E-16 | 2.25 | 7.9 | 10.15 |
| 2069_s_at | CTNNA1 | 1.08E-16 | 1.43 | 7.38 | 8.8 |
| 232_at | LAMC1 | 1.10E-16 | 1.83 | 6.42 | 8.25 |
| 1583_at | TNFRSF1B | 1.21E-16 | 1.26 | 6.51 | 7.77 |
| 41807_at | --- | 1.23E-16 | 0.59 | 3.9 | 4.49 |
| 32363_at | CH25H | 1.23E-16 | 1.28 | 3.43 | 4.71 |
| 39692_at | CREB3L2 | 1.28E-16 | 2.19 | 5.23 | 7.42 |
| 37025_at | LITAF | 1.31E-16 | 1.74 | 6.81 | 8.55 |
| 39416_at | TAX1BP3 | 1.36E-16 | 1.22 | 8.09 | 9.31 |
| 859_at | CYP1B1 | 1.72E-16 | 2.77 | 3.56 | 6.33 |
| 1901_s_at | ERBB2 | 1.74E-16 | 0.93 | 6.21 | 7.14 |
| 40866_at | NIPSNAP1 | 1.87E-16 | -1.11 | 8.77 | 7.66 |
| 40078_at | PRSS23 | 2.05E-16 | 1.75 | 6.63 | 8.38 |
| 37026_at | KLF6 | 2.15E-16 | 1.95 | 7.58 | 9.53 |
| 41155_at | CTNNA1 | 2.30E-16 | 1.58 | 8.61 | 10.18 |
| 40017_at | DKFZP586H2123 | 2.58E-16 | 0.88 | 5.81 | 6.69 |
| 37577_at | ARHGAP19 | 2.79E-16 | 0.92 | 4.22 | 5.14 |
| 38918_at | SOX13 | 3.07E-16 | 0.84 | 5.83 | 6.67 |
| 38636_at | ISLR | 4.17E-16 | 1.02 | 7.84 | 8.85 |
| 40398_s_at | MEOX2 | 4.54E-16 | 2.56 | 3.13 | 5.7 |
| 35703_at | PDGFA | 4.61E-16 | 1.71 | 5.9 | 7.62 |
| 40051_at | TRAM2 | 4.67E-16 | 1.14 | 5.86 | 7 |
| 37788_at | --- | 4.73E-16 | 2.18 | 4.49 | 6.67 |
| 37377_i_at | LMNA | 4.76E-16 | 2.03 | 6.5 | 8.52 |
| 547_s_at | NR4A2 | 4.93E-16 | 1.71 | 5.16 | 6.87 |
| 37643_at | FAS | 5.24E-16 | 0.68 | 3.27 | 3.95 |
| 37305_at | EZH2 | 5.27E-16 | -1.63 | 7.13 | 5.5 |
| 37177_at | CD58 | 5.35E-16 | 1.29 | 4.66 | 5.95 |
| 38978_at | PAIP1 | 5.80E-16 | -1.67 | 6.56 | 4.89 |
| 40350_at | ADAM23 | 5.84E-16 | 0.95 | 5.5 | 6.45 |
| 344_s_at | CNP | 7.65E-16 | 0.71 | 8.4 | 9.11 |
| 38052_at | F13A1 | 7.71E-16 | 2.65 | 5.42 | 8.07 |
| 34760_at | CD302 | 8.41E-16 | 0.94 | 3.61 | 4.55 |
| 36040_at | SH3BGR | 8.63E-16 | 1.32 | 4.7 | 6.03 |
| 709_at | TUBB | 8.89E-16 | -0.97 | 8.82 | 7.86 |
| 1249_at | GAB1 | 9.05E-16 | 0.84 | 3.31 | 4.14 |
| 40475_at | CAPN6 | 9.27E-16 | 1.27 | 4.17 | 5.43 |
| 38833_at | HLA-DPA1 | 9.48E-16 | 2.47 | 8.97 | 11.44 |
| 1052_s_at | CEBPD | 1.07E-15 | 3.23 | 5.93 | 9.16 |
| 39133_at | BLOC1S1 | 1.14E-15 | 0.85 | 6.2 | 7.05 |
| 38888_at | LGI1 | 1.29E-15 | 1.1 | 3.02 | 4.12 |
| 33929_at | GPC1 | 1.34E-15 | 1.21 | 7.56 | 8.77 |
| 37805_at | SRPX2 | 1.45E-15 | 0.78 | 4.96 | 5.74 |
| 41644_at | SASH1 | 1.48E-15 | 1.19 | 7.59 | 8.78 |
| 40210_at | RAB13 | 1.57E-15 | 1.56 | 7.49 | 9.05 |
| 38842_at | AMOTL2 | 1.70E-15 | 1.7 | 4.74 | 6.45 |
| 581_at | LAMB1 | 1.74E-15 | 1.79 | 6.29 | 8.08 |
| 37442_at | DKFZp586I1420 | 1.81E-15 | 1.07 | 7.31 | 8.39 |
| 34446_at | RABGAP1L | 1.92E-15 | 0.68 | 4.1 | 4.77 |
| 37001_at | CAPN2 | 2.26E-15 | 1.14 | 7.64 | 8.78 |
| 36159_s_at | PRNP | 2.46E-15 | 2.75 | 5.41 | 8.16 |
| 32143_at | OSR2 | 2.51E-15 | 0.94 | 4.25 | 5.19 |
| 39593_at | FGL2 | 2.53E-15 | 1.66 | 5.6 | 7.25 |
| 37230_at | KLHL21 | 2.59E-15 | 1.05 | 4.78 | 5.83 |
| 41138_at | CD99 | 2.85E-15 | 1.61 | 8.32 | 9.93 |
| 40646_at | CX3CR1 | 2.88E-15 | 1.81 | 3.34 | 5.15 |
| 39043_at | ARPC1B | 3.04E-15 | 1.57 | 7.66 | 9.23 |
| 37598_at | RASSF2 | 3.06E-15 | 1.24 | 5.5 | 6.74 |
| 36069_at | SRGAP2 | 3.10E-15 | 1.08 | 5.03 | 6.12 |
| 1491_at | PTX3 | 3.11E-15 | 1.68 | 3.62 | 5.3 |
| 35800_at | PAFAH1B3 | 3.13E-15 | -1.28 | 7.66 | 6.38 |
| 40310_at | TLR2 | 3.18E-15 | 0.95 | 4.79 | 5.74 |
| 1398_g_at | MAP3K11 | 3.46E-15 | 0.61 | 7.82 | 8.43 |
| 37281_at | FAM38A | 3.62E-15 | 1.18 | 7.66 | 8.85 |
| 39969_at | HIST1H4C | 4.24E-15 | -2.31 | 7.86 | 5.56 |
| 36116_at | TRIP10 | 4.25E-15 | 1.03 | 5.7 | 6.73 |
| 36915_at | CTSO | 4.30E-15 | 0.71 | 6.24 | 6.95 |
| 41140_at | IFNGR2 | 4.61E-15 | 1.01 | 8.01 | 9.02 |
| 41156_g_at | CTNNA1 | 4.85E-15 | 1.45 | 7.47 | 8.92 |
| 38075_at | SYPL1 | 4.93E-15 | 1.98 | 6.29 | 8.27 |
| 41549_s_at | AP1S2 | 5.03E-15 | 1.82 | 5.54 | 7.36 |
| 39240_at | NRXN1 | 5.12E-15 | 2.24 | 5.68 | 7.93 |
| 1467_at | EPS8 | 5.55E-15 | 1.63 | 4.94 | 6.57 |
| 41245_at | GDF10 | 6.98E-15 | 0.8 | 3.08 | 3.88 |
| 39839_at | CSDA | 7.53E-15 | 2.63 | 4.16 | 6.8 |
| 35020_at | PHOX2B | 8.31E-15 | -3.38 | 8.93 | 5.55 |
| 41435_at | PPFIA3 | 8.45E-15 | -0.92 | 7.07 | 6.15 |
| 1848_at | RAP1A | 8.63E-15 | 1.09 | 4.4 | 5.5 |
| 36689_at | OSBPL1A | 8.89E-15 | 0.61 | 3.66 | 4.27 |
| 37352_at | SP100 | 9.22E-15 | 1.09 | 4.71 | 5.8 |
| 35783_at | VAMP3 | 9.36E-15 | 1.23 | 7.07 | 8.3 |
| 38565_at | GAL3ST1 | 9.83E-15 | 0.9 | 5.5 | 6.4 |
| 37534_at | CXADR | 1.00E-14 | -2.26 | 6.9 | 4.64 |
| 33182_at | NTRK2 | 1.10E-14 | 1.18 | 3.87 | 5.05 |
| 32587_at | ZFP36L2 | 1.10E-14 | 1.56 | 6.32 | 7.88 |
| 32321_at | HLA-E | 1.12E-14 | 2.07 | 7.07 | 9.15 |
| 38740_at | ZFP36L1 | 1.12E-14 | 1.42 | 8.03 | 9.46 |
| 339_at | CAV2 | 1.16E-14 | 1.57 | 3.14 | 4.71 |
| 1891_at | MAP3K8 | 1.19E-14 | 0.75 | 3.17 | 3.92 |
| 34907_at | AATK | 1.33E-14 | 0.72 | 8.2 | 8.91 |
| 540_at | HSPB2 | 1.38E-14 | 0.71 | 7.62 | 8.33 |
| 39781_at | IGFBP4 | 1.43E-14 | 2.69 | 8.8 | 11.48 |
| 1737_s_at | IGFBP4 | 1.61E-14 | 2.58 | 7.94 | 10.53 |
| 38407_r_at | PTGDS | 1.75E-14 | 1.92 | 6.93 | 8.85 |
| 33997_at | --- | 1.80E-14 | 0.63 | 3.16 | 3.79 |
| 39224_at | CENTD1 | 1.83E-14 | 0.65 | 2.66 | 3.31 |
| 38064_at | MVP | 2.04E-14 | 1.1 | 7.24 | 8.34 |
| 36596_r_at | GATM | 2.05E-14 | 3.13 | 3.51 | 6.64 |
| 34409_at | LRP10 | 2.08E-14 | 0.88 | 7.06 | 7.95 |
| 38312_at | OLFML2A | 2.15E-14 | 2.06 | 5.88 | 7.95 |
| 36174_at | MARCKSL1 | 2.17E-14 | -1.72 | 10.58 | 8.86 |
| 34389_at | COL14A1 | 2.35E-14 | 0.58 | 5.35 | 5.93 |
| 37415_at | ATP10B | 2.59E-14 | 0.64 | 5.07 | 5.71 |
| 37028_at | PPP1R15A | 2.69E-14 | 1.21 | 7.12 | 8.33 |
| 277_at | MCL1 | 2.71E-14 | 1.4 | 8.12 | 9.52 |
| 38824_at | HTATIP2 | 2.73E-14 | 1.09 | 4.71 | 5.8 |
| 40020_at | CELSR3 | 2.84E-14 | -1.28 | 8.11 | 6.83 |
| 36595_s_at | GATM | 2.85E-14 | 3.19 | 3.19 | 6.38 |
| 32079_at | KIF13B | 2.92E-14 | 0.76 | 6.26 | 7.01 |
| 612_s_at | CNP | 2.98E-14 | 0.9 | 6.42 | 7.32 |
| 31881_at | SGMS1 | 3.06E-14 | 1.01 | 2.96 | 3.97 |
| 39301_at | CAPN3 | 3.07E-14 | 0.59 | 6.47 | 7.06 |
| 35396_at | HAS2 | 3.16E-14 | 0.77 | 3.75 | 4.53 |
| 39628_at | RAB9A | 3.25E-14 | 0.81 | 6.16 | 6.97 |
| 39021_at | DPY19L1 | 3.42E-14 | 1.1 | 3.36 | 4.46 |
| 39775_at | SERPING1 | 3.89E-14 | 2.47 | 8.48 | 10.96 |
| 38775_at | LRP1 | 4.02E-14 | 1 | 6.89 | 7.89 |
| 32166_at | TLN1 | 4.18E-14 | 1.62 | 8.6 | 10.22 |
| 1717_s_at | BIRC3 | 4.38E-14 | 1.62 | 3.72 | 5.34 |
| 2085_s_at | CTNNA1 | 4.55E-14 | 1.3 | 6.57 | 7.88 |
| 40832_s_at | TOR1AIP1 | 4.62E-14 | 1.37 | 5.3 | 6.66 |
| 1897_at | TGFBR3 | 4.62E-14 | 1.08 | 4.57 | 5.65 |
| 33146_at | MCL1 | 4.76E-14 | 1.45 | 7.72 | 9.17 |
| 36156_at | AQP1 | 5.24E-14 | 1.64 | 7.45 | 9.1 |
| 32598_at | NELL2 | 5.39E-14 | -2.23 | 8.22 | 5.99 |
| 37190_at | WASF1 | 5.59E-14 | -1.57 | 5.98 | 4.41 |
| 41766_at | MAN2A2 | 5.62E-14 | 0.89 | 6.68 | 7.57 |
| 39801_at | PLOD3 | 6.54E-14 | 0.61 | 8.07 | 8.68 |
| 36711_at | MAFF | 6.73E-14 | 2.61 | 4.73 | 7.34 |
| 151_s_at | TUBB | 6.95E-14 | -1.25 | 12.18 | 10.93 |
| 35951_at | NRXN3 | 7.24E-14 | 0.58 | 5.41 | 5.98 |
| 35735_at | GBP1 | 7.30E-14 | 1.05 | 3.98 | 5.02 |
| 33830_at | LEPROT | 7.46E-14 | 1.2 | 6.44 | 7.64 |
| 32765_f_at | PGCP | 7.63E-14 | 0.85 | 5.55 | 6.4 |
| 39544_at | DMN | 7.66E-14 | 1.65 | 4.23 | 5.88 |
| 133_at | CTSC | 7.73E-14 | 1.79 | 6.32 | 8.1 |
| 41419_at | GULP1 | 9.33E-14 | 2.05 | 3.22 | 5.26 |
| 41154_r_at | CTNNA1 | 9.76E-14 | 0.6 | 2.89 | 3.48 |
| 36920_at | MTM1 | 1.01E-13 | 0.47 | 2.95 | 3.42 |
| 37307_at | GNAI2 | 1.03E-13 | 1.14 | 9.36 | 10.51 |
| 39266_at | --- | 1.11E-13 | 1.08 | 3.28 | 4.36 |
| 33412_at | LGALS1 | 1.13E-13 | 2.82 | 8.5 | 11.32 |
| 39310_at | BDKRB2 | 1.14E-13 | 0.92 | 5.59 | 6.51 |
| 36750_at | RIMBP2 | 1.22E-13 | -2.55 | 8.46 | 5.91 |
| 38823_s_at | STK17A | 1.24E-13 | 0.61 | 3.12 | 3.73 |
| 32764_at | PGCP | 1.27E-13 | 0.55 | 4.98 | 5.53 |
| 34407_at | RARRES2 | 1.30E-13 | 2 | 7.98 | 9.98 |
| 1229_at | MTMR11 | 1.32E-13 | 0.85 | 4.31 | 5.16 |
| 195_s_at | CASP4 | 1.32E-13 | 1.83 | 4.08 | 5.91 |
| 39852_at | SPG20 | 1.36E-13 | 0.6 | 4.73 | 5.33 |
| 35698_at | CFI | 1.43E-13 | 1.81 | 4.68 | 6.49 |
| 34759_at | SLC30A1 | 1.43E-13 | 0.86 | 5.64 | 6.5 |
| 38087_s_at | S100A4 | 1.53E-13 | 1.36 | 6.75 | 8.12 |
| 38138_at | S100A11 | 1.54E-13 | 1.71 | 6.96 | 8.66 |
| 41830_at | KIAA0494 | 1.59E-13 | 1.38 | 6.69 | 8.07 |
| 33371_s_at | RAB31 | 1.60E-13 | 1.5 | 6.97 | 8.47 |
| 35016_at | CD74 | 1.69E-13 | 2.64 | 8.36 | 11 |
| 34091_s_at | VIM | 1.71E-13 | 2.14 | 10.53 | 12.66 |
| 34663_at | FCGR2B | 1.81E-13 | 0.78 | 5.85 | 6.64 |
| 37353_g_at | SP100 | 1.88E-13 | 1.25 | 4.66 | 5.91 |
| 36039_s_at | SH3BGR | 1.98E-13 | 0.69 | 4.58 | 5.28 |
| 40496_at | C1S | 2.01E-13 | 3.11 | 7.05 | 10.16 |
| 41745_at | IFITM3 | 2.02E-13 | 2.37 | 10.25 | 12.62 |
| 37003_at | CD63 | 2.15E-13 | 1.31 | 8.28 | 9.59 |
| 287_at | ATF3 | 2.17E-13 | 1.77 | 5.09 | 6.85 |
| 32579_at | SMARCA4 | 2.19E-13 | -1.38 | 7.99 | 6.61 |
| 33790_at | CCL14 | 2.35E-13 | 1.42 | 6.86 | 8.29 |
| 38132_at | CDC42EP1 | 2.42E-13 | 0.67 | 8.53 | 9.19 |
| 377_g_at | SEMA3C | 2.75E-13 | 2.62 | 3.29 | 5.91 |
| 39014_at | FCGBP | 2.92E-13 | 1.45 | 5.79 | 7.24 |
| 41222_at | STAT6 | 3.02E-13 | 0.82 | 6.82 | 7.64 |
| 37747_at | ANXA5 | 3.20E-13 | 1.74 | 6.79 | 8.53 |
| 32154_at | TFAP2A | 3.37E-13 | 1.18 | 4.43 | 5.62 |
| 37985_at | LMNB1 | 3.43E-13 | -1.42 | 6.64 | 5.22 |
| 36829_at | PER1 | 3.64E-13 | 0.67 | 8.16 | 8.83 |
| 36211_at | BCL2L2 | 3.67E-13 | 0.72 | 6.39 | 7.12 |
| 38095_i_at | HLA-DPB1 | 3.86E-13 | 2.7 | 8.6 | 11.3 |
| 1062_g_at | IL10RA | 4.37E-13 | 0.98 | 5.46 | 6.44 |
| 908_at | IFIT2 | 4.42E-13 | 0.54 | 5.25 | 5.79 |
| 33450_at | ACTL6B | 4.44E-13 | -1.07 | 7.46 | 6.39 |
| 34526_s_at | LOC157627 | 4.59E-13 | -1.65 | 7.86 | 6.21 |
| 34712_at | DNMBP | 5.13E-13 | 0.65 | 6.2 | 6.85 |
| 41609_at | HLA-DMB | 5.38E-13 | 1.43 | 7.26 | 8.69 |
| 35755_at | ITPK1 | 5.53E-13 | 0.56 | 8.08 | 8.64 |
| 38812_at | LAMB2 | 5.54E-13 | 1.13 | 6.93 | 8.05 |
| 34730_g_at | TRO | 5.74E-13 | -1.15 | 8.75 | 7.6 |
| 41723_s_at | hCG_1998957 | 6.07E-13 | 3.31 | 6.54 | 9.86 |
| 32107_at | C21orf25 | 6.10E-13 | 1.32 | 5.77 | 7.09 |
| 32450_at | SCN7A | 6.12E-13 | 0.63 | 3.73 | 4.36 |
| 34729_at | TRO | 6.26E-13 | -1.16 | 5.43 | 4.27 |
| 39070_at | FSCN1 | 6.26E-13 | -1.5 | 10.65 | 9.15 |
| 1394_at | RHOA | 6.41E-13 | 0.71 | 8.74 | 9.45 |
| 40063_at | CALCOCO2 | 6.62E-13 | 1.16 | 5.91 | 7.08 |
| 41397_at | LOC55565 | 6.63E-13 | -0.61 | 6.87 | 6.26 |
| 33883_at | EFS | 6.73E-13 | 1.09 | 5.5 | 6.58 |
| 34296_at | MID1 | 6.97E-13 | 0.92 | 5.94 | 6.86 |
| 1568_s_at | IFNAR2 | 7.20E-13 | 0.92 | 3.18 | 4.1 |
| 33109_f_at | SOX2 | 7.30E-13 | 0.77 | 6.62 | 7.39 |
| 1466_s_at | FGF7 | 7.57E-13 | 0.72 | 2.9 | 3.62 |
| 41418_at | LPHN1 | 7.71E-13 | -0.93 | 8.67 | 7.74 |
| 103_at | THBS4 | 8.05E-13 | 1.91 | 6.08 | 7.99 |
| 35844_at | SDC4 | 8.08E-13 | 1.16 | 4.51 | 5.67 |
| 464_s_at | IFI35 | 8.12E-13 | 0.85 | 6.42 | 7.27 |
| 35174_i_at | EEF1A2 | 8.47E-13 | -2.22 | 10.05 | 7.83 |
| 40196_at | CTDSPL | 8.66E-13 | 0.92 | 6.39 | 7.31 |
| 35718_at | SP110 | 9.20E-13 | 0.84 | 4.49 | 5.33 |
| 39038_at | FBLN5 | 9.21E-13 | 2.27 | 5.18 | 7.45 |
| 40931_at | TMED5 | 9.27E-13 | 1.15 | 5.27 | 6.42 |
| 38487_at | STAB1 | 9.91E-13 | 1.41 | 7.83 | 9.24 |
| 41531_at | TM4SF1 | 9.97E-13 | 1.99 | 4.01 | 5.99 |
| 1385_at | TGFBI | 1.02E-12 | 2.39 | 6.23 | 8.62 |
| 769_s_at | ANXA2 | 1.19E-12 | 2.22 | 10.05 | 12.28 |
| 947_at | MCM7 | 1.42E-12 | -1.63 | 8.76 | 7.13 |
| 32783_at | FBLN2 | 1.49E-12 | 0.92 | 7 | 7.93 |
| 36980_at | PNRC1 | 1.51E-12 | 1.12 | 4.59 | 5.72 |
| 31444_s_at | ANXA2 | 1.52E-12 | 2.1 | 10.41 | 12.51 |
| 36644_at | CD151 | 1.70E-12 | 0.72 | 8.29 | 9.02 |
| 36007_at | OLFML2B | 1.71E-12 | 1.07 | 7.26 | 8.32 |
| 32607_at | BASP1 | 1.75E-12 | -2.02 | 10.63 | 8.61 |
| 33385_g_at | CAST | 1.77E-12 | 0.74 | 4.21 | 4.95 |
| 41569_at | DNAJC9 | 1.88E-12 | -1.12 | 6.59 | 5.47 |
| 41690_at | ARID5B | 1.93E-12 | 1.84 | 5.48 | 7.32 |
| 38512_r_at | ELAVL3 | 1.94E-12 | -0.48 | 8.18 | 7.7 |
| 38342_at | PHF15 | 1.97E-12 | 0.57 | 7.3 | 7.87 |
| 34403_at | MFGE8 | 1.99E-12 | 0.87 | 7.12 | 8 |
| 37688_f_at | FCGR2A | 2.02E-12 | 1.68 | 3.01 | 4.69 |
| 33372_at | RAB31 | 2.03E-12 | 0.86 | 6.9 | 7.76 |
| 37684_at | SLCO2B1 | 2.17E-12 | 1.53 | 5.69 | 7.22 |
| 1723_g_at | ERBB3 | 2.21E-12 | 0.74 | 5.57 | 6.31 |
| 41472_at | APOBEC3G | 2.24E-12 | 0.5 | 2.94 | 3.44 |
| 1620_at | CDH6 | 2.27E-12 | 0.75 | 4.89 | 5.64 |
| 35237_at | COL21A1 | 2.55E-12 | 0.82 | 4.52 | 5.33 |
| 38251_at | MYL6B | 2.57E-12 | -1.08 | 8.57 | 7.49 |
| 1537_at | EGFR | 2.67E-12 | 1.33 | 5.3 | 6.64 |
| 39109_at | TPX2 | 2.71E-12 | -1.29 | 7.3 | 6.01 |
| 38034_at | STS | 2.76E-12 | 0.55 | 3.55 | 4.11 |
| 40766_at | C4A | 2.78E-12 | 1.9 | 7.64 | 9.54 |
| 845_at | STAT6 | 3.04E-12 | 0.81 | 5.78 | 6.59 |
| 36472_at | NMI | 3.05E-12 | 1.45 | 5.46 | 6.91 |
| 37780_at | PCLO | 3.40E-12 | -1.43 | 6.62 | 5.18 |
| 529_at | DUSP5 | 3.44E-12 | 1.23 | 5.55 | 6.78 |
| 34280_at | GABRE | 3.94E-12 | 0.69 | 5.2 | 5.88 |
| 39588_at | TNFSF12 | 4.01E-12 | 0.43 | 7.62 | 8.04 |
| 31869_at | SWAP70 | 4.01E-12 | 0.97 | 4.56 | 5.53 |
| 40380_at | ELAVL4 | 4.03E-12 | -1.61 | 5.5 | 3.89 |
| 38403_at | LAMP2 | 4.07E-12 | 1.46 | 3.93 | 5.39 |
| 33102_at | ADD3 | 4.14E-12 | 1.47 | 7.55 | 9.02 |
| 31697_s_at | FTH1 | 4.17E-12 | 1.03 | 11.11 | 12.14 |
| 40091_at | BCL6 | 4.32E-12 | 1.77 | 5.04 | 6.81 |
| 32120_at | SPAG5 | 4.53E-12 | -1.29 | 5.76 | 4.47 |
| 38220_at | DPYD | 4.62E-12 | 1.3 | 4.07 | 5.38 |
| 40855_at | SAMD4A | 4.64E-12 | 1.09 | 4.88 | 5.97 |
| 32250_at | CFH | 4.71E-12 | 1.66 | 5.81 | 7.47 |
| 31886_at | NT5E | 4.78E-12 | 0.91 | 4.38 | 5.29 |
| 35688_g_at | MTCP1 | 4.82E-12 | 1 | 5.93 | 6.93 |
| 39712_at | S100A13 | 4.99E-12 | 1.19 | 6.11 | 7.3 |
| 36941_at | MLLT11 | 5.06E-12 | -1.73 | 10.6 | 8.87 |
| 1049_g_at | RXRG | 5.12E-12 | 0.77 | 6.6 | 7.36 |
| 40698_at | CLEC2B | 5.23E-12 | 1.32 | 3.66 | 4.99 |
| 202_at | HSF2 | 5.46E-12 | -0.92 | 6 | 5.08 |
| 41788_i_at | TSC22D2 | 5.54E-12 | 0.69 | 2.99 | 3.68 |
| 40863_r_at | CKB | 5.62E-12 | -1.13 | 9.78 | 8.65 |
| 1230_g_at | MTMR11 | 5.62E-12 | 0.83 | 5.46 | 6.29 |
| 34324_at | CLN5 | 5.80E-12 | 0.74 | 4.2 | 4.93 |
| 37734_at | MOBK1B | 5.94E-12 | 0.79 | 6.3 | 7.09 |
| 40619_at | UBE2S | 6.28E-12 | -1.45 | 7.99 | 6.54 |
| 36627_at | SPARCL1 | 6.65E-12 | 2.93 | 5.64 | 8.57 |
| 38044_at | FAM107A | 6.80E-12 | 0.52 | 7.24 | 7.76 |
| 35118_at | LCAT | 7.01E-12 | 0.56 | 6.22 | 6.77 |
| 36629_at | TSC22D3 | 7.07E-12 | 1.24 | 7.03 | 8.27 |
| 36970_at | KIAA0182 | 7.22E-12 | -1.13 | 6.96 | 5.83 |
| 38508_s_at | TNXA | 7.32E-12 | 0.8 | 7.58 | 8.39 |
| 34796_at | TRAM1 | 7.35E-12 | 1.03 | 3.37 | 4.4 |
| 1825_at | IQGAP1 | 7.68E-12 | 1.22 | 6.27 | 7.48 |
| 676_g_at | IFITM1 | 7.75E-12 | 2.28 | 10.29 | 12.57 |
| 33249_at | NR3C2 | 7.82E-12 | 0.64 | 5.07 | 5.7 |
| 32188_at | MYT1 | 7.89E-12 | -1.66 | 6.35 | 4.69 |
| 37600_at | ECM1 | 7.93E-12 | 0.48 | 6.47 | 6.95 |
| 33759_at | BPGM | 8.22E-12 | 0.95 | 5.16 | 6.11 |
| 33943_at | FTH1 | 8.32E-12 | 1.16 | 8.65 | 9.8 |
| 892_at | TM4SF1 | 8.51E-12 | 1.63 | 5.75 | 7.38 |
| 1321_s_at | EMP1 | 8.63E-12 | 0.79 | 6.1 | 6.89 |
| 37032_at | NNMT | 8.97E-12 | 2.12 | 6.64 | 8.76 |
| 34224_at | FADS3 | 9.60E-12 | 0.85 | 7.45 | 8.3 |
| 39758_f_at | LAMP1 | 9.60E-12 | 1.17 | 9.3 | 10.46 |
| 39592_r_at | FGL2 | 9.74E-12 | 0.52 | 2.92 | 3.44 |
| 37618_at | HOXB7 | 9.99E-12 | 0.48 | 7.02 | 7.51 |
| 35614_at | TCFL5 | 1.01E-11 | 1 | 4.22 | 5.22 |
| 32905_s_at | TPSAB1 | 1.02E-11 | 1.35 | 5.56 | 6.9 |
| 36601_at | VCL | 1.04E-11 | 1.2 | 6.58 | 7.77 |
| 34412_s_at | GP1BB | 1.04E-11 | -1.61 | 7.98 | 6.38 |
| 40014_at | SEMA6C | 1.09E-11 | -0.57 | 6.42 | 5.85 |
| 376_at | SEMA3C | 1.19E-11 | 2.57 | 5.04 | 7.6 |
| 33134_at | ADCY3 | 1.24E-11 | 0.47 | 7.4 | 7.87 |
| 39965_at | RAC3 | 1.34E-11 | -0.94 | 9.02 | 8.08 |
| 41523_at | RAB32 | 1.37E-11 | 0.64 | 3.47 | 4.11 |
| 909_g_at | IFIT2 | 1.38E-11 | 0.61 | 4.25 | 4.86 |
| 33733_at | ABCG2 | 1.38E-11 | 0.51 | 4.34 | 4.85 |
| 35811_at | RNF13 | 1.39E-11 | 1.08 | 4.99 | 6.07 |
| 36130_f_at | MT1E | 1.40E-11 | 1.9 | 8.52 | 10.42 |
| 38653_at | PMP22 | 1.47E-11 | 2.67 | 9.01 | 11.68 |
| 32806_at | TSPO | 1.57E-11 | 1.17 | 8.52 | 9.7 |
| 37136_at | SH2B2 | 1.57E-11 | -0.68 | 7.52 | 6.84 |
| 32824_at | TPP1 | 1.59E-11 | 1.25 | 5.83 | 7.08 |
| 37272_at | ITPKB | 1.60E-11 | 0.73 | 6.77 | 7.51 |
| 32526_at | JAM3 | 1.73E-11 | 0.68 | 7.25 | 7.93 |
| 784_g_at | WWP1 | 1.86E-11 | 0.99 | 4.48 | 5.47 |
| 37545_at | SCAMP5 | 1.93E-11 | -0.95 | 8.64 | 7.7 |
| 32606_at | BASP1 | 2.02E-11 | -1.3 | 5.4 | 4.1 |
| 40685_at | ALDH3B1 | 2.06E-11 | 0.61 | 5.82 | 6.43 |
| 675_at | IFITM1 | 2.08E-11 | 1.62 | 8.01 | 9.64 |
| 31508_at | TXNIP | 2.08E-11 | 1.39 | 8.69 | 10.08 |
| 34808_at | KIAA0999 | 2.08E-11 | 0.84 | 6.66 | 7.51 |
| 1031_at | SRPK1 | 2.16E-11 | -1.11 | 5.48 | 4.37 |
| 783_at | WWP1 | 2.18E-11 | 1.08 | 5.68 | 6.76 |
| 609_f_at | MT1B | 2.29E-11 | 1.35 | 8.23 | 9.58 |
| 34276_at | RP4-691N24.1 | 2.30E-11 | -1.06 | 5.92 | 4.86 |
| 1828_s_at | FGF2 | 2.32E-11 | 0.66 | 3.66 | 4.32 |
| 31801_at | TMF1 | 2.48E-11 | -1.29 | 7.72 | 6.43 |
| 39707_at | MTMR4 | 2.59E-11 | -1.34 | 7.29 | 5.96 |
| 35962_at | MAST1 | 2.63E-11 | -1.19 | 7.4 | 6.22 |
| 1782_s_at | STMN1 | 2.72E-11 | -0.89 | 8.35 | 7.46 |
| 35146_at | TGFB1I1 | 2.76E-11 | 0.62 | 6.99 | 7.61 |
| 38822_at | STK17A | 2.88E-11 | 0.45 | 5.71 | 6.15 |
| 38857_r_at | KLHL23 | 3.10E-11 | -0.69 | 3.51 | 2.81 |
| 38510_at | --- | 3.13E-11 | 0.93 | 3.1 | 4.04 |
| 38427_at | COL15A1 | 3.16E-11 | 2.14 | 5.07 | 7.21 |
| 38354_at | CEBPB | 3.19E-11 | 2.43 | 6.91 | 9.34 |
| 32842_at | BCL7A | 3.22E-11 | -1.34 | 6.52 | 5.19 |
| 41314_at | CS | 3.23E-11 | -0.62 | 8.72 | 8.1 |
| 34368_at | HDAC2 | 3.31E-11 | -1.42 | 8.11 | 6.69 |
| 41704_at | C6orf137 | 3.46E-11 | -1.3 | 8.16 | 6.86 |
| 40024_at | STAC | 3.58E-11 | -1.26 | 7.16 | 5.9 |
| 40746_at | GRIA2 | 3.61E-11 | -2.79 | 7.58 | 4.79 |
| 38022_s_at | PLEC1 | 3.70E-11 | 0.78 | 6.59 | 7.37 |
| 40365_at | GNA15 | 3.73E-11 | 0.78 | 5.56 | 6.34 |
| 37039_at | HLA-DRA | 3.86E-11 | 2.64 | 9.95 | 12.58 |
| 33505_at | RARRES1 | 3.95E-11 | 1.1 | 4.65 | 5.75 |
| 574_s_at | CASP1 | 3.95E-11 | 0.63 | 4.42 | 5.05 |
| 39903_at | CIB2 | 4.09E-11 | -0.49 | 7.26 | 6.77 |
| 816_g_at | DOK1 | 4.16E-11 | 0.54 | 7.33 | 7.88 |
| 37009_at | CAT | 4.23E-11 | 1.27 | 5.31 | 6.58 |
| 40862_i_at | CKB | 4.24E-11 | -1.12 | 9.25 | 8.13 |
| 40808_at | CHGA | 4.39E-11 | -2.19 | 8.6 | 6.41 |
| 40578_s_at | TMOD1 | 4.63E-11 | -1.9 | 8.09 | 6.19 |
| 35545_at | SLC4A8 | 4.64E-11 | -1.42 | 6.26 | 4.84 |
| 34563_at | KIF14 | 4.67E-11 | -0.97 | 6.07 | 5.1 |
| 38204_at | KIAA0406 | 4.78E-11 | -0.7 | 6.73 | 6.02 |
| 34181_at | AGA | 4.85E-11 | 0.68 | 3.85 | 4.53 |
| 41481_at | ITGA2 | 5.12E-11 | 0.6 | 3.56 | 4.15 |
| 40407_at | KPNA2 | 5.15E-11 | -1.47 | 7.17 | 5.7 |
| 34882_at | NOL5A | 5.25E-11 | -1.02 | 6.77 | 5.75 |
| 40399_r_at | MEOX2 | 5.27E-11 | 1.17 | 4.24 | 5.41 |
| 39597_at | ABLIM3 | 5.30E-11 | 1.82 | 6.45 | 8.27 |
| 32002_at | GFRA3 | 5.40E-11 | 0.93 | 8.3 | 9.23 |
| 35104_r_at | LMO1 | 5.53E-11 | -0.78 | 7.61 | 6.83 |
| 31788_at | ZBED4 | 5.64E-11 | -0.65 | 5.59 | 4.94 |
| 33429_at | C18orf10 | 5.82E-11 | -1.05 | 7.6 | 6.55 |
| 38913_at | HFE | 5.83E-11 | 0.39 | 3.49 | 3.89 |
| 893_at | LOC731049 | 5.84E-11 | -1.47 | 8.75 | 7.28 |
| 39729_at | PRDX2 | 5.93E-11 | -1.04 | 8.87 | 7.83 |
| 37671_at | LAMA4 | 6.16E-11 | 1.98 | 5.41 | 7.39 |
| 33426_at | CHGB | 6.32E-11 | -3.08 | 9.21 | 6.13 |
| 40817_at | NUCB1 | 6.48E-11 | 0.61 | 8.21 | 8.82 |
| 36187_at | RNH1 | 6.49E-11 | 0.74 | 7.06 | 7.79 |
| 37375_at | PHLDB1 | 6.67E-11 | 0.87 | 6.95 | 7.82 |
| 39503_s_at | DPYSL4 | 6.92E-11 | -0.87 | 7.63 | 6.76 |
| 34189_at | SETDB1 | 7.03E-11 | -0.75 | 7.39 | 6.64 |
| 41651_at | KIAA1033 | 7.10E-11 | 0.91 | 6.29 | 7.2 |
| 32116_at | TMC6 | 7.13E-11 | 0.61 | 7.28 | 7.89 |
| 41409_at | C1orf38 | 7.16E-11 | 0.86 | 5.45 | 6.31 |
| 31431_at | FCGRT | 7.16E-11 | 1.21 | 7.53 | 8.74 |
| 32142_at | STK3 | 7.35E-11 | 0.83 | 3.32 | 4.15 |
| 33103_s_at | ADD3 | 7.45E-11 | 1.49 | 4.74 | 6.24 |
| 36393_at | DPF1 | 7.81E-11 | -0.58 | 6.93 | 6.36 |
| 31830_s_at | SMTN | 8.38E-11 | 0.92 | 7.87 | 8.8 |
| 37210_at | INA | 8.47E-11 | -1.72 | 9.52 | 7.8 |
| 31893_at | ARL2 | 8.75E-11 | 0.6 | 7.37 | 7.97 |
| 40775_at | ITM2A | 8.78E-11 | 1.72 | 3.41 | 5.13 |
| 34905_at | GRIK5 | 8.82E-11 | -0.53 | 8.5 | 7.97 |
| 41816_at | CARD10 | 8.89E-11 | 0.58 | 6.23 | 6.82 |
| 36189_at | ILF2 | 9.37E-11 | -0.89 | 8.39 | 7.5 |
| 34789_at | SERPINB6 | 9.41E-11 | 1.08 | 7.44 | 8.52 |
| 35336_at | UNC84B | 9.42E-11 | 0.6 | 8.42 | 9.02 |
| 32588_s_at | ZFP36L2 | 9.61E-11 | 1.58 | 8.65 | 10.23 |
| 39389_at | CD9 | 1.02E-10 | 2.13 | 5.59 | 7.71 |
| 1042_at | RARRES1 | 1.04E-10 | 1.44 | 3.88 | 5.32 |
| 40330_at | PLCE1 | 1.05E-10 | 1.22 | 3.77 | 4.99 |
| 40692_at | TLE4 | 1.13E-10 | -0.88 | 4.88 | 4 |
| 37589_at | --- | 1.21E-10 | -0.82 | 6.75 | 5.93 |
| 31899_at | TTC35 | 1.22E-10 | 0.78 | 3.71 | 4.48 |
| 37258_at | TMEFF1 | 1.25E-10 | -1.62 | 5.44 | 3.83 |
| 39250_at | NOV | 1.30E-10 | 1.01 | 3.78 | 4.79 |
| 35754_at | TMED10 | 1.33E-10 | 1.14 | 7.45 | 8.59 |
| 38438_at | NFKB1 | 1.33E-10 | 0.45 | 8.49 | 8.94 |
| 38005_at | SLC35D2 | 1.33E-10 | 0.83 | 3.87 | 4.7 |
| 1020_s_at | CIB1 | 1.37E-10 | 0.95 | 6.71 | 7.67 |
| 40726_at | KIF11 | 1.37E-10 | -1.76 | 5.98 | 4.22 |
| 41859_at | UST | 1.43E-10 | 0.54 | 3.63 | 4.17 |
| 35366_at | NID1 | 1.43E-10 | 1.87 | 6.56 | 8.42 |
| 1813_at | --- | 1.45E-10 | 0.56 | 6.24 | 6.81 |
| 40000_f_at | VPS13A | 1.63E-10 | -0.66 | 5.08 | 4.42 |
| 31874_at | GAS2L1 | 1.63E-10 | 0.94 | 6.04 | 6.98 |
| 35842_at | IL6ST | 1.66E-10 | 1.67 | 5.44 | 7.11 |
| 38652_at | C10orf26 | 1.68E-10 | 0.79 | 6.75 | 7.55 |
| 34739_at | FNBP1L | 1.68E-10 | -1.65 | 6.28 | 4.63 |
| 39603_at | DISC1 | 1.72E-10 | 0.47 | 5.41 | 5.89 |
| 32851_at | CUGBP2 | 1.73E-10 | 0.68 | 3.94 | 4.62 |
| 40369_f_at | HLA-G | 1.75E-10 | 0.91 | 7.62 | 8.53 |
| 40038_at | ST7 | 1.78E-10 | -0.48 | 8.11 | 7.63 |
| 32111_at | CDYL | 1.79E-10 | 1.01 | 5.54 | 6.55 |
| 31623_f_at | MT1A | 1.86E-10 | 1.47 | 8.31 | 9.78 |
| 34797_at | PPAP2A | 1.86E-10 | 1.53 | 5.16 | 6.69 |
| 40797_at | ADAM10 | 1.87E-10 | 0.78 | 5.52 | 6.31 |
| 38096_f_at | HLA-DPB1 | 1.87E-10 | 2.8 | 7.43 | 10.23 |
| 37304_at | CBX1 | 1.89E-10 | -1.65 | 8.44 | 6.79 |
| 40416_at | SMARCB1 | 1.96E-10 | -0.37 | 8.07 | 7.7 |
| 1072_g_at | GATA2 | 1.99E-10 | -0.94 | 8.16 | 7.22 |
| 36199_at | DAP | 1.99E-10 | 0.82 | 7.65 | 8.47 |
| 38121_at | WARS | 1.99E-10 | 0.7 | 7.72 | 8.43 |
| 1461_at | NFKBIA | 2.01E-10 | 1.38 | 7.99 | 9.36 |
| 31432_g_at | FCGRT | 2.01E-10 | 1.17 | 8.25 | 9.42 |
| 2093_s_at | XRCC5 | 2.03E-10 | -1.1 | 8.88 | 7.78 |
| 38160_at | LY75 | 2.04E-10 | 0.78 | 2.74 | 3.51 |
| 40420_at | STK10 | 2.05E-10 | 0.54 | 6.13 | 6.66 |
| 38416_at | CCT6A | 2.05E-10 | -0.91 | 7.61 | 6.69 |
| 38739_at | ETS2 | 2.05E-10 | 0.76 | 4.34 | 5.1 |
| 1651_at | UBE2C | 2.11E-10 | -1.66 | 8.35 | 6.69 |
| 37579_at | CYFIP2 | 2.15E-10 | -0.83 | 8.3 | 7.47 |
| 33630_s_at | SPTBN2 | 2.20E-10 | -0.87 | 6.72 | 5.84 |
| 32145_at | ADD1 | 2.47E-10 | 0.59 | 9.08 | 9.67 |
| 36694_at | KCNS3 | 2.51E-10 | 1.28 | 3.43 | 4.71 |
| 33975_at | BTC | 2.52E-10 | 0.41 | 2.93 | 3.34 |
| 33193_at | WIPI1 | 2.72E-10 | 0.71 | 3.16 | 3.87 |
| 41707_at | SARM1 | 2.87E-10 | -0.72 | 5.65 | 4.93 |
| 35907_at | CCNF | 2.94E-10 | -0.81 | 5.98 | 5.17 |
| 33834_at | CXCL12 | 2.97E-10 | 1.16 | 6.86 | 8.01 |
| 34390_at | P4HA2 | 2.98E-10 | 1.18 | 5.64 | 6.82 |
| 32260_at | PEA15 | 3.17E-10 | 1.11 | 8.31 | 9.42 |
| 32629_f_at | BTN3A1 | 3.25E-10 | 0.81 | 8.2 | 9 |
| 35134_at | ART3 | 3.40E-10 | 0.99 | 2.89 | 3.89 |
| 35699_at | BUB1B | 3.50E-10 | -1.69 | 5.57 | 3.89 |
| 38163_at | RIMS2 | 3.50E-10 | -1.54 | 5.5 | 3.97 |
| 36031_at | ING1 | 3.53E-10 | 0.42 | 5.37 | 5.79 |
| 37099_at | ALOX5AP | 3.62E-10 | 1.03 | 4.96 | 5.99 |
| 31800_at | --- | 3.63E-10 | -1.23 | 7.43 | 6.2 |
| 37706_at | GLG1 | 3.64E-10 | 0.48 | 7.56 | 8.04 |
| 35695_at | LYST | 3.68E-10 | 0.63 | 5.33 | 5.96 |
| 38767_at | SPRY1 | 3.69E-10 | 1.16 | 6.46 | 7.62 |
| 38047_at | RBPMS | 3.71E-10 | 1.02 | 5.72 | 6.74 |
| 34660_at | RNASE6 | 3.73E-10 | 1.2 | 4.93 | 6.13 |
| 32775_r_at | PLSCR1 | 3.76E-10 | 1.6 | 5.41 | 7.01 |
| 1377_at | NFKB1 | 4.14E-10 | 0.84 | 6.05 | 6.88 |
| 37302_at | CENPF | 4.15E-10 | -1.97 | 5.62 | 3.65 |
| 1376_at | LIG1 | 4.29E-10 | -0.7 | 7.33 | 6.62 |
| 37563_at | SRGAP3 | 4.37E-10 | -1.44 | 8.16 | 6.71 |
| 37981_at | DBN1 | 4.52E-10 | -0.95 | 8.25 | 7.3 |
| 39337_at | H2AFZ | 4.61E-10 | -1.37 | 7.24 | 5.87 |
| 36148_at | APLP1 | 4.66E-10 | -1.07 | 9.22 | 8.15 |
| 626_s_at | IFI35 | 4.80E-10 | 0.67 | 6.18 | 6.85 |
| 39350_at | GPC3 | 5.03E-10 | 2.4 | 5.32 | 7.71 |
| 38397_at | POLD4 | 5.08E-10 | 0.71 | 7.5 | 8.21 |
| 310_s_at | MAPT | 5.08E-10 | -1.14 | 6.51 | 5.38 |
| 38078_at | FLNB | 5.12E-10 | 0.84 | 5.63 | 6.47 |
| 485_at | DHH | 5.14E-10 | 0.67 | 5.18 | 5.85 |
| 37911_at | STX4 | 5.15E-10 | 0.79 | 6.25 | 7.05 |
| 1444_at | PSMD9 | 5.26E-10 | 0.39 | 6.78 | 7.17 |
| 35479_at | ADAM28 | 5.52E-10 | 0.55 | 3.38 | 3.93 |
| 32148_at | FARP1 | 5.63E-10 | -0.95 | 7.29 | 6.34 |
| 39556_at | SPTBN1 | 5.66E-10 | 0.75 | 6.24 | 7 |
| 37220_at | FCGR1A | 5.77E-10 | 0.86 | 4.81 | 5.67 |
| 34840_at | --- | 5.90E-10 | 1.39 | 4.12 | 5.5 |
| 1242_at | ERF | 5.92E-10 | 0.64 | 7.62 | 8.26 |
| 36119_at | CAV1 | 6.32E-10 | 1.86 | 4.9 | 6.75 |
| 37558_at | IGF2BP3 | 6.38E-10 | -1.84 | 5.36 | 3.52 |
| 36916_at | ST3GAL4 | 6.52E-10 | 0.41 | 7.94 | 8.35 |
| 34375_at | CCL2 | 6.54E-10 | 2.13 | 7.08 | 9.21 |
| 753_at | NID2 | 6.61E-10 | 1.65 | 4.54 | 6.19 |
| 32943_at | DBH | 6.64E-10 | -2.44 | 9.1 | 6.67 |
| 35995_at | ZWINT | 6.64E-10 | -1.73 | 6.49 | 4.77 |
| 1137_at | SLC20A2 | 6.83E-10 | 0.47 | 4.9 | 5.36 |
| 38241_at | BTN3A3 | 6.86E-10 | 1.19 | 4.14 | 5.33 |
| 38364_at | TLE4 | 7.18E-10 | -1.81 | 6.08 | 4.27 |
| 38671_at | PLXND1 | 7.28E-10 | 0.87 | 7.76 | 8.62 |
| 36128_at | TMED10 | 7.31E-10 | 0.91 | 7.26 | 8.17 |
| 32800_at | RXRA | 7.44E-10 | 0.82 | 6.53 | 7.35 |
| 40755_at | MICA | 7.46E-10 | 0.52 | 6.49 | 7.01 |
| 40145_at | TOP2A | 7.50E-10 | -1.88 | 6.33 | 4.44 |
| 32087_at | HSF2 | 7.80E-10 | -0.94 | 5.42 | 4.48 |
| 37979_at | YAP1 | 7.80E-10 | 0.38 | 2.89 | 3.26 |
| 33331_at | MALL | 7.81E-10 | 0.66 | 6.43 | 7.08 |
| 34363_at | SEPP1 | 7.94E-10 | 2.03 | 6.31 | 8.34 |
| 41224_at | ASCC3L1 | 7.96E-10 | -0.92 | 9.04 | 8.12 |
| 38120_at | PKD2 | 7.99E-10 | 0.8 | 5.2 | 5.99 |
| 1378_g_at | NFKB1 | 8.08E-10 | 0.52 | 6.4 | 6.92 |
| 35784_at | VAMP3 | 8.56E-10 | 0.58 | 4.33 | 4.91 |
| 36963_at | PGD | 8.66E-10 | -0.84 | 8.97 | 8.12 |
| 1346_at | MT3 | 8.68E-10 | 0.56 | 7.73 | 8.29 |
| 33266_at | AURKB | 9.01E-10 | -1.11 | 8.18 | 7.07 |
| 33807_at | PLEKHA6 | 9.12E-10 | -0.88 | 6.71 | 5.83 |
| 512_at | NR1H3 | 9.16E-10 | 1.08 | 5.41 | 6.49 |
| 39714_at | SH3BGRL | 9.26E-10 | 1.46 | 5.29 | 6.75 |
| 39649_at | ARHGAP4 | 9.37E-10 | 0.52 | 7.44 | 7.96 |
| 32092_at | SDC3 | 9.48E-10 | 1.07 | 8.85 | 9.92 |
| 40968_at | SOCS3 | 9.50E-10 | 0.87 | 6.56 | 7.43 |
| 1102_s_at | NR3C1 | 9.73E-10 | 0.7 | 4.69 | 5.39 |
| 32652_g_at | TAGLN3 | 9.82E-10 | -1.15 | 7.17 | 6.03 |
| 36956_at | SLC20A2 | 9.95E-10 | 0.49 | 5.56 | 6.06 |
| 37724_at | MYC | 1.01E-09 | 1.33 | 4.47 | 5.8 |
| 1012_at | PCAF | 1.02E-09 | 0.58 | 3.55 | 4.13 |
| 41754_at | LRPPRC | 1.02E-09 | -0.93 | 6.89 | 5.96 |
| 40764_at | GOT2 | 1.04E-09 | -0.85 | 7.95 | 7.1 |
| 37011_at | AIF1 | 1.04E-09 | 1.5 | 4.25 | 5.75 |
| 1441_s_at | FAS | 1.04E-09 | 0.37 | 2.48 | 2.85 |
| 38772_at | CYR61 | 1.05E-09 | 2.74 | 5.87 | 8.61 |
| 35103_i_at | LMO1 | 1.05E-09 | -1.74 | 5.83 | 4.09 |
| 37345_at | CALU | 1.08E-09 | 0.94 | 6.41 | 7.35 |
| 894_g_at | UBE2S | 1.08E-09 | -1.28 | 9.22 | 7.94 |
| 36194_at | LRPAP1 | 1.09E-09 | 0.63 | 6.94 | 7.57 |
| 39145_at | MYL9 | 1.10E-09 | 1.57 | 7.8 | 9.37 |
| 32249_at | CFH | 1.11E-09 | 2.35 | 5.38 | 7.73 |
| 40555_at | RHOQ | 1.11E-09 | 0.88 | 6.12 | 7 |
| 36578_at | BIRC2 | 1.12E-09 | 0.81 | 5.75 | 6.56 |
| 39286_at | TOB2 | 1.14E-09 | 0.64 | 6.58 | 7.22 |
| 33239_at | CCDC46 | 1.14E-09 | 0.47 | 2.59 | 3.06 |
| 37024_at | LITAF | 1.15E-09 | 2 | 6.34 | 8.35 |
| 41122_at | AOF2 | 1.16E-09 | -1 | 7.49 | 6.49 |
| 32878_f_at | PPP1R3E | 1.17E-09 | -0.59 | 7.51 | 6.92 |
| 39413_at | OPHN1 | 1.18E-09 | 0.55 | 2.81 | 3.36 |
| 1769_at | MAST1 | 1.18E-09 | -0.76 | 7.62 | 6.86 |
| 31944_at | TULP3 | 1.22E-09 | 0.74 | 6.85 | 7.59 |
| 41806_at | FGF2 | 1.23E-09 | 0.41 | 4.39 | 4.8 |
| 36138_at | CAPNS1 | 1.24E-09 | 0.92 | 9.08 | 10.01 |
| 31622_f_at | MT1F | 1.24E-09 | 1.48 | 8.43 | 9.91 |
| 33871_s_at | FOLR2 | 1.28E-09 | 0.54 | 7.06 | 7.59 |
| 32773_at | LOC731682 | 1.29E-09 | 1.3 | 6.33 | 7.63 |
| 40039_g_at | ST7 | 1.30E-09 | -0.61 | 6.7 | 6.09 |
| 37962_r_at | STXBP3 | 1.30E-09 | 0.92 | 4.14 | 5.06 |
| 40984_at | 76P | 1.31E-09 | -0.77 | 6.71 | 5.94 |
| 36921_at | DYNLT3 | 1.37E-09 | 1.36 | 6.07 | 7.43 |
| 35909_at | PHLDA1 | 1.39E-09 | 0.63 | 6.79 | 7.42 |
| 35365_at | ILK | 1.42E-09 | 0.75 | 7.75 | 8.5 |
| 37402_at | RNASE1 | 1.46E-09 | 2.22 | 6.23 | 8.45 |
| 40690_at | CKS2 | 1.49E-09 | -1.41 | 6.75 | 5.34 |
| 41837_at | C14orf132 | 1.49E-09 | -1.6 | 9.13 | 7.53 |
| 203_at | GATA2 | 1.50E-09 | -1.24 | 8.32 | 7.08 |
| 38788_at | LOC161527 | 1.51E-09 | 0.32 | 6.93 | 7.25 |
| 34854_at | CIAPIN1 | 1.51E-09 | -0.39 | 5.68 | 5.3 |
| 36610_at | R3HDM1 | 1.53E-09 | -0.96 | 6.07 | 5.11 |
| 34793_s_at | PLS3 | 1.55E-09 | 1.84 | 4.71 | 6.56 |
| 38937_at | AP3B2 | 1.55E-09 | -0.54 | 8.37 | 7.83 |
| 41457_at | KIAA0423 | 1.58E-09 | 0.67 | 5.31 | 5.99 |
| 34528_at | LOC157627 | 1.61E-09 | -0.76 | 5.34 | 4.58 |
| 40370_f_at | HLA-G | 1.64E-09 | 0.85 | 7.64 | 8.49 |
| 38996_at | ERF | 1.73E-09 | 0.8 | 7.2 | 8 |
| 628_at | FZD2 | 1.75E-09 | 1.04 | 5.76 | 6.8 |
| 33334_at | ACYP1 | 1.77E-09 | -0.97 | 6.52 | 5.55 |
| 34761_r_at | ADAM9 | 1.79E-09 | 0.68 | 2.81 | 3.49 |
| 32141_at | PPM1E | 1.79E-09 | -0.92 | 5.81 | 4.89 |
| 33261_at | HLA-DRB1 | 1.82E-09 | 1.45 | 8.81 | 10.25 |
| 35625_at | CD97 | 1.92E-09 | 0.39 | 7.91 | 8.31 |
| 39345_at | NPC2 | 1.95E-09 | 1.83 | 7.33 | 9.16 |
| 1505_at | TYMS | 1.98E-09 | -1.53 | 7.16 | 5.63 |
| 585_at | XRCC5 | 1.99E-09 | -1.03 | 9.36 | 8.34 |
| 40767_at | TFPI | 2.00E-09 | 1.53 | 4.78 | 6.31 |
| 39594_f_at | MT1H | 2.01E-09 | 1.45 | 8.71 | 10.16 |
| 201_s_at | B2M | 2.06E-09 | 1.57 | 10.23 | 11.8 |
| 39149_at | PRCC | 2.26E-09 | -0.49 | 7.6 | 7.11 |
| 33342_at | SNUPN | 2.27E-09 | 0.39 | 5.98 | 6.37 |
| 39037_at | AFF1 | 2.28E-09 | 0.93 | 6.26 | 7.19 |
| 34362_at | SLC2A5 | 2.31E-09 | 0.67 | 4.42 | 5.09 |
| 39424_at | TNFRSF14 | 2.37E-09 | 0.6 | 7.28 | 7.89 |
| 39557_at | LOC201229 | 2.41E-09 | 0.61 | 4.91 | 5.52 |
| 34433_at | DOK1 | 2.41E-09 | 0.46 | 7.08 | 7.54 |
| 40936_at | CRIM1 | 2.44E-09 | 1.64 | 5.82 | 7.46 |
| 174_s_at | ITSN2 | 2.44E-09 | 0.99 | 5.62 | 6.61 |
| 36900_at | STIM1 | 2.48E-09 | 0.51 | 6.6 | 7.11 |
| 39568_g_at | AQP7 | 2.49E-09 | 0.49 | 7.18 | 7.67 |
| 36902_at | RHOG | 2.53E-09 | 0.46 | 7.17 | 7.64 |
| 37326_at | PLP2 | 2.57E-09 | 0.78 | 6.73 | 7.51 |
| 874_at | CCL2 | 2.65E-09 | 0.62 | 4.96 | 5.58 |
| 32332_at | IDH2 | 2.67E-09 | -0.72 | 7.48 | 6.76 |
| 37495_at | PRR3 | 2.68E-09 | -0.88 | 6.23 | 5.35 |
| 1592_at | TOP2A | 2.70E-09 | -1.91 | 7.3 | 5.39 |
| 33641_g_at | AIF1 | 2.72E-09 | 0.88 | 6.27 | 7.15 |
| 39381_at | C6orf120 | 2.72E-09 | 0.54 | 5.33 | 5.87 |
| 36780_at | CLU | 2.76E-09 | 1.89 | 10.12 | 12.01 |
| 38038_at | LUM | 2.77E-09 | 2.73 | 5.53 | 8.26 |
| 32562_at | ENG | 2.81E-09 | 1.11 | 7.04 | 8.15 |
| 39044_s_at | DGKD | 2.85E-09 | -0.76 | 8.03 | 7.26 |
| 37544_at | NFIL3 | 2.87E-09 | 1.13 | 5.96 | 7.09 |
| 31675_s_at | PTEN | 2.89E-09 | 0.63 | 3.36 | 3.99 |
| 931_at | EBI2 | 2.93E-09 | 0.86 | 3.62 | 4.48 |
| 35675_at | SORBS3 | 3.01E-09 | 0.43 | 8.29 | 8.72 |
| 40920_at | CDC14B | 3.05E-09 | 0.51 | 4.02 | 4.53 |
| 40665_at | FMO3 | 3.06E-09 | 1.63 | 3.47 | 5.1 |
| 33728_at | BSN | 3.14E-09 | -0.41 | 6.53 | 6.12 |
| 40865_at | TDG | 3.17E-09 | -0.98 | 6.02 | 5.04 |
| 32544_s_at | RSU1 | 3.20E-09 | 0.81 | 5.8 | 6.61 |
| 36084_at | CUL7 | 3.29E-09 | -0.37 | 7.81 | 7.44 |
| 37169_at | ZNF536 | 3.31E-09 | 0.89 | 4.56 | 5.45 |
| 38737_at | IGF1 | 3.36E-09 | 1.05 | 2.81 | 3.86 |
| 32970_f_at | HABP4 | 3.39E-09 | 0.49 | 6.72 | 7.21 |
| 41191_at | PALLD | 3.40E-09 | 1.21 | 7.63 | 8.83 |
| 33302_at | SSPN | 3.43E-09 | 0.43 | 2.93 | 3.36 |
| 36823_at | RPIP8 | 3.56E-09 | -1.07 | 7.96 | 6.89 |
| 31897_at | FILIP1L | 3.61E-09 | 1.39 | 5.28 | 6.67 |
| 37421_f_at | HLA-F | 3.62E-09 | 1.17 | 8.29 | 9.46 |
| 34669_at | TFE3 | 3.73E-09 | 0.41 | 5.98 | 6.39 |
| 38287_at | PSMB9 | 3.74E-09 | 0.95 | 6.52 | 7.46 |
| 875_g_at | CCL2 | 3.74E-09 | 2.25 | 7.42 | 9.67 |
| 33838_at | BAT3 | 3.75E-09 | -0.6 | 9.34 | 8.74 |
| 36052_at | ADD2 | 3.77E-09 | -0.47 | 6.88 | 6.41 |
| 39178_at | RTN1 | 3.87E-09 | -1.93 | 9.19 | 7.26 |
| 936_s_at | --- | 3.89E-09 | -1.29 | 7.91 | 6.62 |
| 32751_at | ILF3 | 3.92E-09 | -1.07 | 7.66 | 6.6 |
| 36576_at | H2AFY | 3.94E-09 | -0.94 | 8.09 | 7.15 |
| 257_at | ITGAV | 3.97E-09 | 0.38 | 2.87 | 3.25 |
| 36272_r_at | PMP2 | 3.98E-09 | 0.42 | 2.36 | 2.77 |
| 37309_at | RHOA | 3.99E-09 | 0.91 | 10.05 | 10.96 |
| 40774_at | CCT3 | 4.16E-09 | -1.09 | 8.82 | 7.74 |
| 1150_at | --- | 4.22E-09 | 0.71 | 4.35 | 5.06 |
| 40067_at | ELF1 | 4.37E-09 | 0.66 | 3.14 | 3.8 |
| 38733_at | XRCC5 | 4.43E-09 | -0.93 | 9.8 | 8.87 |
| 37347_at | CKS1B | 4.46E-09 | -1.22 | 7.51 | 6.29 |
| 33956_at | LY96 | 4.51E-09 | 1.11 | 3.66 | 4.77 |
| 34182_at | NDST1 | 4.58E-09 | 0.42 | 7.41 | 7.83 |
| 33131_at | SOX4 | 4.65E-09 | -1.89 | 9.96 | 8.07 |
| 36965_at | ANK3 | 4.66E-09 | 2.07 | 6.64 | 8.7 |
| 38995_at | CLDN5 | 4.67E-09 | 0.85 | 7.25 | 8.1 |
| 35012_at | MNDA | 4.74E-09 | 0.98 | 3.23 | 4.21 |
| 39677_at | GINS1 | 4.75E-09 | -1.16 | 5.16 | 4 |
| 39964_at | RP2 | 4.94E-09 | 0.91 | 3.5 | 4.41 |
| 518_at | NR1H2 | 5.13E-09 | 0.51 | 5.85 | 6.37 |
| 34812_at | GORASP1 | 5.15E-09 | 0.37 | 5.48 | 5.85 |
| 38856_at | ADAMTSL3 | 5.17E-09 | 0.92 | 3.47 | 4.39 |
| 36593_at | EXT2 | 5.18E-09 | 0.45 | 5.88 | 6.33 |
| 37670_at | ANXA7 | 5.23E-09 | 1.4 | 5.25 | 6.65 |
| 35017_f_at | HLA-G | 5.23E-09 | 0.94 | 7.38 | 8.31 |
| 33108_i_at | SOX2 | 5.27E-09 | 0.72 | 4.77 | 5.49 |
| 32916_at | PTPRE | 5.38E-09 | 0.59 | 4.15 | 4.74 |
| 31792_at | ANXA3 | 5.42E-09 | 1.17 | 3.1 | 4.27 |
| 36600_at | PSME1 | 5.54E-09 | 0.75 | 7.1 | 7.85 |
| 40955_at | PTOV1 | 5.61E-09 | -0.74 | 8.67 | 7.93 |
| 40763_at | MEIS1 | 5.66E-09 | -1.59 | 5.78 | 4.19 |
| 38158_at | ESPL1 | 5.73E-09 | -0.77 | 7.18 | 6.4 |
| 36628_at | RALBP1 | 5.82E-09 | 0.96 | 6.87 | 7.83 |
| 1055_g_at | RFC4 | 5.84E-09 | -1.3 | 5.79 | 4.49 |
| 39879_s_at | LRRC37A2 | 5.89E-09 | -1.02 | 8.6 | 7.57 |
| 38546_at | IL1RAP | 6.01E-09 | 0.77 | 5.01 | 5.78 |
| 41193_at | DUSP6 | 6.07E-09 | 1.85 | 5.84 | 7.69 |
| 32749_s_at | FLNA | 6.08E-09 | 1.18 | 8.74 | 9.92 |
| 1499_at | FNTA | 6.11E-09 | 0.71 | 8.21 | 8.91 |
| 1519_at | ETS2 | 6.11E-09 | 0.93 | 5.65 | 6.58 |
| 32634_s_at | ICA1 | 6.19E-09 | -1.38 | 8.02 | 6.65 |
| 36908_at | MRC1 | 6.42E-09 | 1.04 | 4.19 | 5.23 |
| 37825_at | GALK2 | 6.59E-09 | 0.47 | 4.63 | 5.1 |
| 35531_at | NOS1AP | 6.61E-09 | -0.79 | 7.38 | 6.58 |
| 35329_at | CYB5R1 | 6.86E-09 | 0.54 | 6.75 | 7.28 |
| 38116_at | KIAA0101 | 6.99E-09 | -1.34 | 6.68 | 5.34 |
| 38651_at | FEZ2 | 7.01E-09 | 0.87 | 7.44 | 8.31 |
| 39694_at | TMEM109 | 7.13E-09 | 0.35 | 8.45 | 8.79 |
| 38151_at | LOH11CR2A | 7.19E-09 | 0.62 | 4.76 | 5.38 |
| 38101_at | ZCCHC14 | 7.31E-09 | -1.06 | 8.28 | 7.22 |
| 39346_at | KHDRBS1 | 7.41E-09 | -0.88 | 8.48 | 7.6 |
| 692_s_at | SOD3 | 7.43E-09 | 0.63 | 7.68 | 8.31 |
| 36200_at | EHMT2 | 7.48E-09 | -0.56 | 7.66 | 7.1 |
| 39704_s_at | HMGA1 | 7.70E-09 | -1.11 | 9 | 7.89 |
| 37741_at | PYCR1 | 7.90E-09 | -0.74 | 7.7 | 6.96 |
| 33825_at | SERPINA3 | 7.92E-09 | 1.91 | 6.85 | 8.75 |
| 41709_at | PPFIBP2 | 7.93E-09 | 0.6 | 6.54 | 7.14 |
| 41856_at | UNC5B | 8.05E-09 | -0.35 | 8.01 | 7.66 |
| 35234_at | RECK | 8.07E-09 | 0.59 | 4.11 | 4.7 |
| 39827_at | DDIT4 | 8.43E-09 | 1.56 | 6.18 | 7.74 |
| 41770_at | MAOA | 8.52E-09 | -2.35 | 7.45 | 5.1 |
| 41591_at | WDR68 | 8.63E-09 | -0.93 | 7.63 | 6.7 |
| 32979_at | GAB1 | 8.78E-09 | 0.52 | 4.19 | 4.7 |
| 32104_i_at | CAMK2G | 8.98E-09 | -0.47 | 8.56 | 8.09 |
| 35151_at | CDK2AP2 | 9.11E-09 | 0.42 | 7.97 | 8.39 |
| 40898_at | SQSTM1 | 9.18E-09 | 0.61 | 7.98 | 8.59 |
| 41835_at | NOL4 | 9.21E-09 | -0.67 | 4.73 | 4.06 |
| 172_at | INPP5D | 9.44E-09 | 0.63 | 6.52 | 7.14 |
| 38631_at | TNFAIP2 | 9.57E-09 | 1.19 | 5.44 | 6.63 |
| 41446_f_at | MT1F | 9.80E-09 | 1.41 | 8.34 | 9.75 |
| 32650_at | TAGLN3 | 1.04E-08 | -1.36 | 8.81 | 7.45 |
| 35778_at | KIF5C | 1.06E-08 | -1.89 | 10.19 | 8.3 |
| 36091_at | SKAP2 | 1.08E-08 | 1.28 | 3.49 | 4.76 |
| 40419_at | STOM | 1.09E-08 | 1.43 | 8.07 | 9.5 |
| 31831_at | SMTN | 1.11E-08 | 0.48 | 7.53 | 8 |
| 37378_r_at | LMNA | 1.11E-08 | 0.7 | 7.55 | 8.25 |
| 1945_at | CCNB1 | 1.13E-08 | -1.32 | 5.05 | 3.72 |
| 935_at | CAP1 | 1.15E-08 | 0.92 | 8.03 | 8.95 |
| 35730_at | ADH1B | 1.16E-08 | 1.95 | 3.41 | 5.35 |
| 39566_at | CHRFAM7A | 1.17E-08 | -2.03 | 8.26 | 6.23 |
| 39152_f_at | COIL | 1.20E-08 | -0.67 | 6.63 | 5.96 |
| 1380_at | FGF7 | 1.23E-08 | 0.66 | 3.03 | 3.69 |
| 41195_at | LPP | 1.23E-08 | 0.72 | 4.09 | 4.81 |
| 35347_at | EFEMP2 | 1.24E-08 | 0.49 | 6.2 | 6.69 |
| 41544_at | PLK2 | 1.26E-08 | 1.46 | 5.06 | 6.51 |
| 36042_at | NTRK2 | 1.26E-08 | 1.01 | 4.11 | 5.11 |
| 40448_at | ZFP36 | 1.29E-08 | 2.51 | 8.21 | 10.72 |
| 38139_at | FPGT | 1.29E-08 | 0.62 | 4.58 | 5.2 |
| 32227_at | SRGN | 1.29E-08 | 2.07 | 4.45 | 6.52 |
| 35645_at | SNX1 | 1.31E-08 | 0.65 | 5.39 | 6.05 |
| 40836_s_at | EML3 | 1.31E-08 | 0.33 | 7.05 | 7.39 |
| 41675_at | SNAP91 | 1.32E-08 | -1.64 | 7.84 | 6.2 |
| 40185_at | GFAP | 1.32E-08 | 0.6 | 5.64 | 6.24 |
| 41400_at | TK1 | 1.32E-08 | -0.8 | 7.47 | 6.67 |
| 280_g_at | NR4A1 | 1.34E-08 | 2.35 | 7.01 | 9.36 |
| 279_at | NR4A1 | 1.37E-08 | 1.65 | 6.77 | 8.42 |
| 37533_r_at | TTC9 | 1.37E-08 | -1.16 | 4.37 | 3.22 |
| 38893_at | NCF4 | 1.37E-08 | 0.64 | 6.78 | 7.42 |
| 1985_s_at | NME1 | 1.40E-08 | -1.27 | 8.9 | 7.63 |
| 36231_at | PIK3IP1 | 1.40E-08 | 0.42 | 6.62 | 7.03 |
| 40522_at | GLUL | 1.41E-08 | 0.88 | 7.17 | 8.05 |
| 40049_at | DAPK1 | 1.42E-08 | -0.96 | 5.17 | 4.21 |
| 39114_at | C10orf10 | 1.42E-08 | 1.27 | 6.9 | 8.18 |
| 34851_at | AURKA | 1.42E-08 | -0.6 | 6.76 | 6.17 |
| 32725_at | BID | 1.43E-08 | -0.54 | 6.87 | 6.33 |
| 36453_at | KBTBD11 | 1.45E-08 | -1.05 | 5.78 | 4.73 |
| 34862_at | SCCPDH | 1.46E-08 | 0.66 | 6.29 | 6.95 |
| 37899_at | TYMS | 1.52E-08 | -1.15 | 5.26 | 4.1 |
| 35622_at | SHC2 | 1.52E-08 | -0.87 | 8.11 | 7.23 |
| 41346_at | LARGE | 1.53E-08 | 0.52 | 6.03 | 6.55 |
| 39506_at | BICC1 | 1.53E-08 | 0.87 | 3.01 | 3.88 |
| 34382_at | DCX | 1.54E-08 | -2.31 | 7.96 | 5.64 |
| 32251_at | TCEAL4 | 1.54E-08 | 0.63 | 6.93 | 7.55 |
| 40417_at | CCT5 | 1.57E-08 | -0.96 | 7.81 | 6.85 |
| 38720_at | CCT7 | 1.57E-08 | -0.65 | 8.71 | 8.06 |
| 40552_s_at | LOC728499 | 1.58E-08 | -1.37 | 7.95 | 6.57 |
| 37265_at | RIMS3 | 1.60E-08 | -1.74 | 7.45 | 5.71 |
| 39091_at | ARL6IP5 | 1.63E-08 | 0.71 | 5.38 | 6.09 |
| 38756_at | RAP1A | 1.64E-08 | 0.4 | 3.87 | 4.27 |
| 1434_at | PTEN | 1.67E-08 | 0.36 | 3.41 | 3.77 |
| 37426_at | TOX3 | 1.72E-08 | -1.44 | 3.91 | 2.46 |
| 36749_at | CPA3 | 1.75E-08 | 0.56 | 3.88 | 4.44 |
| 38819_at | PTK7 | 1.85E-08 | -0.54 | 6.56 | 6.02 |
| 38363_at | TYROBP | 1.86E-08 | 1.59 | 7.48 | 9.07 |
| 33944_at | APLP2 | 1.92E-08 | 0.94 | 8.06 | 9 |
| 39812_at | MRPL12 | 1.93E-08 | -0.78 | 7.53 | 6.75 |
| 860_at | MSH2 | 1.93E-08 | -1.06 | 6.54 | 5.48 |
| 32696_at | PBX3 | 1.94E-08 | -1.26 | 6.67 | 5.41 |
| 34715_at | FOXM1 | 1.95E-08 | -1.01 | 6.99 | 5.97 |
| 1854_at | MYBL2 | 1.95E-08 | -1.39 | 6.49 | 5.1 |
| 41162_at | PPM1G | 1.99E-08 | -0.73 | 8.99 | 8.26 |
| 32553_at | MAZ | 1.99E-08 | -0.5 | 10.33 | 9.83 |
| 36856_at | PLEKHQ1 | 2.03E-08 | 0.43 | 6.93 | 7.36 |
| 469_at | EFNB3 | 2.04E-08 | -1.32 | 7.74 | 6.42 |
| 33304_at | ISG20 | 2.04E-08 | 0.5 | 6.51 | 7.01 |
| 39673_i_at | ECM2 | 2.05E-08 | 0.98 | 6.52 | 7.5 |
| 34942_at | CNIH3 | 2.06E-08 | 0.5 | 3.29 | 3.79 |
| 37588_s_at | MAPK8IP2 | 2.08E-08 | -0.85 | 8.34 | 7.49 |
| 37225_at | ANKRD15 | 2.10E-08 | 0.91 | 3.23 | 4.14 |
| 34273_at | RGS4 | 2.10E-08 | -1.61 | 7.5 | 5.89 |
| 879_at | MX2 | 2.12E-08 | 0.69 | 5.96 | 6.65 |
| 32776_at | RALB | 2.12E-08 | 0.47 | 6.1 | 6.57 |
| 39387_at | SEPHS1 | 2.15E-08 | -0.66 | 6.38 | 5.73 |
| 38086_at | IGSF3 | 2.16E-08 | -1.53 | 6.33 | 4.8 |
| 37565_at | MMD | 2.16E-08 | -1.27 | 7.87 | 6.6 |
| 904_s_at | TOP2A | 2.20E-08 | -1.51 | 5.16 | 3.65 |
| 39400_at | TBC1D2B | 2.21E-08 | 0.4 | 7.98 | 8.38 |
| 35153_at | NBN | 2.23E-08 | 0.95 | 5.95 | 6.89 |
| 32802_at | 6-Mar | 2.23E-08 | -0.99 | 6.08 | 5.09 |
| 41439_at | MYO1B | 2.26E-08 | -1.05 | 7.38 | 6.32 |
| 37722_s_at | DHPS | 2.26E-08 | -0.73 | 8.22 | 7.48 |
| 39388_at | CAMK2G | 2.26E-08 | -0.91 | 7.87 | 6.97 |
| 38278_at | ARID5A | 2.34E-08 | 0.57 | 6.7 | 7.27 |
| 39031_at | COX7A1 | 2.37E-08 | 1.44 | 6.88 | 8.32 |
| 32536_at | EBP | 2.41E-08 | -0.58 | 7.35 | 6.78 |
| 39872_at | GTSE1 | 2.42E-08 | -0.58 | 7.39 | 6.82 |
| 32090_at | NMNAT2 | 2.42E-08 | -1.21 | 7.24 | 6.03 |
| 39668_at | TFAP2B | 2.45E-08 | -1.98 | 8.05 | 6.08 |
| 38805_at | TGIF1 | 2.45E-08 | 1.36 | 5.06 | 6.42 |
| 1501_at | IGF1 | 2.46E-08 | 1.54 | 4.41 | 5.95 |
| 336_at | TBXA2R | 2.48E-08 | 0.5 | 6.91 | 7.4 |
| 38993_r_at | TGOLN2 | 2.55E-08 | 0.83 | 5.42 | 6.25 |
| 36661_s_at | CD14 | 2.57E-08 | 1.24 | 8.27 | 9.51 |
| 1873_at | XPC | 2.68E-08 | 0.56 | 7.33 | 7.88 |
| 37877_at | NAT9 | 2.78E-08 | -0.42 | 6.74 | 6.31 |
| 41779_at | RGS16 | 2.79E-08 | 0.81 | 6.53 | 7.34 |
| 39297_at | MAB21L1 | 2.83E-08 | -2.26 | 7.94 | 5.68 |
| 1147_at | --- | 2.83E-08 | 1.46 | 4.47 | 5.93 |
| 915_at | IFIT1 | 2.89E-08 | 1.1 | 5.02 | 6.12 |
| 39332_at | TUBB2B | 2.90E-08 | -1.42 | 12.18 | 10.75 |
| 31865_at | DBF4 | 2.92E-08 | -0.44 | 3.21 | 2.77 |
| 32105_f_at | CAMK2G | 2.94E-08 | -0.78 | 7.62 | 6.85 |
| 34118_at | ATP1B2 | 2.96E-08 | 0.49 | 4.26 | 4.75 |
| 38174_at | PSD | 2.96E-08 | -0.69 | 8.09 | 7.4 |
| 33158_at | KAL1 | 2.98E-08 | 0.63 | 4.27 | 4.9 |
| 39073_at | NME1 | 3.01E-08 | -1.28 | 7.79 | 6.51 |
| 34547_at | TNRC4 | 3.10E-08 | -0.62 | 7.78 | 7.16 |
| 37743_at | FEZ1 | 3.11E-08 | 1.67 | 6.4 | 8.07 |
| 40584_at | NUP88 | 3.14E-08 | -0.56 | 7.07 | 6.51 |
| 39867_at | TUFM | 3.17E-08 | -0.55 | 9.31 | 8.76 |
| 1294_at | UBE1L | 3.23E-08 | 0.66 | 6.83 | 7.49 |
| 40803_at | TMEM123 | 3.24E-08 | 1.41 | 6.19 | 7.6 |
| 32112_s_at | AIM1 | 3.28E-08 | 0.84 | 4.59 | 5.43 |
| 32150_at | GOLGA4 | 3.32E-08 | 0.51 | 4.21 | 4.72 |
| 39034_at | CHMP2B | 3.35E-08 | 0.47 | 3.07 | 3.54 |
| 32034_at | ZNF217 | 3.40E-08 | 1.15 | 4.11 | 5.25 |
| 41384_at | RIPK2 | 3.41E-08 | 0.38 | 4.13 | 4.51 |
| 1884_s_at | PCNA | 3.45E-08 | -1.38 | 7.55 | 6.17 |
| 1795_g_at | CCND3 | 3.47E-08 | 0.72 | 7.18 | 7.9 |
| 213_at | ROR1 | 3.52E-08 | 0.49 | 4.57 | 5.06 |
| 37264_at | ZNF131 | 3.53E-08 | -0.59 | 5.31 | 4.72 |
| 254_at | H3F3A | 3.56E-08 | -0.79 | 10.23 | 9.44 |
| 37067_at | LOC728215 | 3.58E-08 | -0.93 | 4.24 | 3.31 |
| 34290_f_at | MED24 | 3.61E-08 | -0.65 | 7.18 | 6.53 |
| 35692_at | TMEM158 | 3.62E-08 | 0.8 | 3.33 | 4.13 |
| 33829_at | LEPROT | 3.67E-08 | 0.86 | 6.07 | 6.93 |
| 39643_at | POLG2 | 3.68E-08 | -0.47 | 5.15 | 4.68 |
| 37721_at | DHPS | 3.77E-08 | -0.35 | 8.72 | 8.38 |
| 1497_at | LTBR | 3.78E-08 | 0.55 | 7.1 | 7.65 |
| 35712_at | LRRN3 | 3.82E-08 | -1.81 | 6.6 | 4.79 |
| 999_at | CYP27A1 | 3.82E-08 | 0.72 | 6.94 | 7.66 |
| 35615_at | BOP1 | 3.83E-08 | -0.9 | 6.9 | 6 |
| 39398_s_at | TBCD | 3.85E-08 | -0.57 | 8.21 | 7.64 |
| 38894_g_at | NCF4 | 3.85E-08 | 0.75 | 4.74 | 5.49 |
| 38346_at | DUS4L | 3.87E-08 | -0.3 | 5.72 | 5.43 |
| 36828_at | ZNF629 | 3.91E-08 | -0.5 | 6.39 | 5.89 |
| 861_g_at | MSH2 | 3.91E-08 | -0.84 | 5.06 | 4.22 |
| 35969_at | MPHOSPH9 | 4.02E-08 | -0.79 | 5.92 | 5.14 |
| 36197_at | CHI3L1 | 4.03E-08 | 1.05 | 4.26 | 5.31 |
| 39567_at | AQP7 | 4.05E-08 | 0.41 | 6.47 | 6.88 |
| 41772_at | MAOA | 4.06E-08 | -2.33 | 7.34 | 5.02 |
| 32066_g_at | CREM | 4.08E-08 | 1.31 | 6.22 | 7.53 |
| 35648_at | AUTS2 | 4.12E-08 | -1.42 | 7.52 | 6.11 |
| 36813_at | TRIP13 | 4.18E-08 | -0.96 | 6.28 | 5.32 |
| 38393_at | KIAA0247 | 4.23E-08 | 0.69 | 6.17 | 6.86 |
| 39552_at | PTEN | 4.26E-08 | 0.44 | 3.84 | 4.28 |
| 38469_at | TSPAN8 | 4.28E-08 | 1.59 | 4.19 | 5.78 |
| 33707_at | PLA2G4C | 4.30E-08 | 0.47 | 6.48 | 6.95 |
| 41035_at | TBKBP1 | 4.30E-08 | -0.37 | 8.15 | 7.78 |
| 39591_s_at | FGL2 | 4.34E-08 | 0.63 | 5.44 | 6.07 |
| 33713_at | TIMM17B | 4.44E-08 | -0.33 | 9.16 | 8.84 |
| 33601_at | ZNF804A | 4.53E-08 | -1.66 | 7.03 | 5.37 |
| 2057_g_at | FGFR1 | 4.54E-08 | 0.79 | 6.94 | 7.73 |
| 39981_at | MSR1 | 4.76E-08 | 0.37 | 2.92 | 3.29 |
| 36645_at | RELA | 4.78E-08 | 0.51 | 7.48 | 8 |
| 40040_at | ST7 | 4.95E-08 | -0.52 | 4.55 | 4.03 |
| 37769_at | EDG4 | 4.97E-08 | -0.32 | 8.64 | 8.32 |
| 35312_at | MCM2 | 5.00E-08 | -1.04 | 8.03 | 6.98 |
| 32717_at | NEURL | 5.04E-08 | -0.52 | 7.3 | 6.77 |
| 40096_at | ATP5A1 | 5.06E-08 | -0.99 | 8.51 | 7.52 |
| 32702_at | TROAP | 5.09E-08 | -0.57 | 7.97 | 7.4 |
| 38085_at | CBX3 | 5.18E-08 | -1.13 | 8.22 | 7.09 |
| 40560_at | TBX2 | 5.19E-08 | -1.13 | 8.24 | 7.11 |
| 37303_at | PARP4 | 5.19E-08 | 0.56 | 4.63 | 5.19 |
| 40710_at | CLGN | 5.23E-08 | -0.8 | 4.37 | 3.57 |
| 41771_g_at | MAOA | 5.27E-08 | -2.35 | 9.62 | 7.27 |
| 41583_at | FEN1 | 5.29E-08 | -1.35 | 7.6 | 6.25 |
| 40260_g_at | RBM9 | 5.38E-08 | -1.17 | 7.24 | 6.07 |
| 31685_at | FEV | 5.38E-08 | -0.81 | 8.68 | 7.86 |
| 41810_at | DKFZp434H1419 | 5.39E-08 | -0.97 | 6.49 | 5.52 |
| 41199_s_at | SFPQ | 5.40E-08 | -0.89 | 7.27 | 6.39 |
| 2003_s_at | MSH6 | 5.41E-08 | -0.98 | 6.12 | 5.14 |
| 39262_at | SAC3D1 | 5.46E-08 | -0.46 | 7.86 | 7.39 |
| 33353_at | ADCY1 | 5.49E-08 | -2.12 | 7.43 | 5.31 |
| 40258_at | CSNK2A1 | 5.49E-08 | -0.43 | 6.69 | 6.26 |
| 34163_g_at | RBPMS | 5.58E-08 | 1.03 | 3.02 | 4.05 |
| 33811_at | CCPG1 | 5.62E-08 | 0.65 | 4.06 | 4.7 |
| 36639_at | ADSL | 5.65E-08 | -0.48 | 6.35 | 5.87 |
| 659_g_at | THBS2 | 5.66E-08 | 1.37 | 6.84 | 8.22 |
| 39535_at | HMX1 | 5.69E-08 | -1.24 | 8.63 | 7.39 |
| 31521_f_at | HIST1H4J | 5.69E-08 | -0.79 | 7.2 | 6.41 |
| 35476_at | SH3PXD2A | 5.73E-08 | 0.46 | 3.13 | 3.59 |
| 32600_at | DOK4 | 5.77E-08 | -0.54 | 6.52 | 5.98 |
| 37698_at | AKAP1 | 5.82E-08 | -0.57 | 5.67 | 5.1 |
| 31820_at | HCLS1 | 5.85E-08 | 1.18 | 6.97 | 8.15 |
| 38774_at | STX7 | 5.88E-08 | 0.88 | 6.54 | 7.42 |
| 32153_s_at | UBB | 5.90E-08 | -0.64 | 11.45 | 10.81 |
| 39003_at | PTTG1IP | 5.93E-08 | 0.81 | 7.72 | 8.53 |
| 39685_at | ATXN10 | 5.98E-08 | -0.76 | 8.71 | 7.95 |
| 1521_at | NME1 | 6.17E-08 | -1.28 | 8.31 | 7.04 |
| 36569_at | CLEC3B | 6.25E-08 | 1.11 | 7 | 8.11 |
| 36249_at | ASPHD1 | 6.33E-08 | -0.79 | 3.81 | 3.02 |
| 37002_at | BLVRB | 6.40E-08 | 0.79 | 6.5 | 7.3 |
| 40820_at | RBM8A | 6.59E-08 | -0.78 | 5.63 | 4.86 |
| 38863_at | RFC5 | 6.70E-08 | -0.55 | 6.68 | 6.14 |
| 40856_at | SERPINF1 | 6.75E-08 | 1.16 | 8.44 | 9.59 |
| 34289_f_at | MED24 | 6.80E-08 | -0.7 | 7.85 | 7.15 |
| 41084_at | ZNF428 | 6.81E-08 | -0.98 | 8.86 | 7.87 |
| 36799_at | FZD2 | 6.93E-08 | 0.65 | 6.63 | 7.28 |
| 1794_at | CCND3 | 6.94E-08 | 0.7 | 7.18 | 7.88 |
| 35849_at | JMJD6 | 6.97E-08 | -0.9 | 7.67 | 6.77 |
| 38299_at | IL6 | 6.98E-08 | 2.11 | 4.14 | 6.25 |
| 38313_at | ABCA2 | 6.98E-08 | 1.02 | 6.82 | 7.84 |
| 41179_at | RNF44 | 7.10E-08 | -0.96 | 7.86 | 6.9 |
| 40140_at | RNF103 | 7.16E-08 | 0.49 | 7.77 | 8.27 |
| 36474_at | KIAA0776 | 7.16E-08 | 0.77 | 4.84 | 5.61 |
| 32340_s_at | YBX1 | 7.30E-08 | -0.75 | 10.36 | 9.61 |
| 123_at | PRKD1 | 7.48E-08 | 0.43 | 3.56 | 3.99 |
| 39292_r_at | TRIM24 | 7.57E-08 | -0.39 | 3.77 | 3.39 |
| 38084_at | CBX3 | 7.66E-08 | -0.95 | 8.03 | 7.08 |
| 40653_at | RGS7 | 7.69E-08 | -1.59 | 5.82 | 4.23 |
| 318_at | H1FX | 7.71E-08 | -0.83 | 7.95 | 7.13 |
| 41254_at | --- | 7.73E-08 | -1.22 | 4.69 | 3.47 |
| 31575_f_at | --- | 7.78E-08 | 0.44 | 6.19 | 6.63 |
| 33106_at | NR1H3 | 7.81E-08 | 0.68 | 5.43 | 6.1 |
| 1647_at | IQGAP2 | 7.99E-08 | 0.79 | 4.46 | 5.25 |
| 36071_at | IPO9 | 8.00E-08 | -0.54 | 5.27 | 4.73 |
| 39120_at | MT1X | 8.00E-08 | 1.21 | 6.16 | 7.38 |
| 35168_f_at | COL16A1 | 8.07E-08 | 2.03 | 5.31 | 7.35 |
| 40402_at | SLC6A2 | 8.14E-08 | -1.16 | 7.13 | 5.97 |
| 34736_at | CCNB1 | 8.27E-08 | -1.05 | 4.88 | 3.84 |
| 38818_at | SPTLC1 | 8.38E-08 | 0.77 | 7.2 | 7.97 |
| 819_at | TIMP4 | 8.61E-08 | 0.7 | 3.38 | 4.09 |
| 36179_at | MAPKAPK2 | 8.79E-08 | 0.56 | 7.46 | 8.02 |
| 40143_at | FAM53B | 8.89E-08 | 0.41 | 5.77 | 6.18 |
| 40099_at | ARHGEF2 | 8.97E-08 | 0.56 | 7.6 | 8.15 |
| 36235_at | --- | 9.05E-08 | -0.47 | 5.62 | 5.16 |
| 35185_at | FABP7 | 9.15E-08 | 0.63 | 4.42 | 5.05 |
| 41710_at | tcag7.1314 | 9.23E-08 | 0.91 | 5.04 | 5.95 |
| 37630_at | CHRDL1 | 9.32E-08 | 0.85 | 4.76 | 5.61 |
| 980_at | NPC1 | 9.46E-08 | 0.5 | 5.62 | 6.12 |
| 36688_at | SCP2 | 9.51E-08 | 1.15 | 5.37 | 6.52 |
| 39348_at | PRMT2 | 9.56E-08 | 0.81 | 7.61 | 8.42 |
| 40387_at | EDG2 | 9.56E-08 | 1.28 | 5.16 | 6.44 |
| 39274_at | NUP62 | 9.71E-08 | -0.51 | 7.22 | 6.71 |
| 39927_at | ARHGAP5 | 9.81E-08 | 0.6 | 3.68 | 4.28 |
| 32213_at | POP7 | 9.99E-08 | -0.39 | 5.9 | 5.51 |
| 40281_at | 2-Sep | 1.03E-07 | 0.58 | 10.09 | 10.67 |
| 1612_s_at | JUND | 1.03E-07 | 0.72 | 10.79 | 11.51 |
| 35807_at | CYBA | 1.03E-07 | 0.82 | 8.25 | 9.07 |
| 33464_at | --- | 1.04E-07 | -0.4 | 5.33 | 4.93 |
| 32209_at | FAM89B | 1.05E-07 | -0.44 | 8.25 | 7.81 |
| 40201_at | DDC | 1.05E-07 | -2.35 | 8.74 | 6.39 |
| 32054_at | CCNT2 | 1.09E-07 | -0.43 | 5.94 | 5.51 |
| 32649_at | TCF7 | 1.09E-07 | 0.89 | 3.38 | 4.26 |
| 35059_at | --- | 1.13E-07 | -0.43 | 5.54 | 5.11 |
| 32060_at | BNIP2 | 1.14E-07 | 0.48 | 5.88 | 6.36 |
| 41526_at | HMG20B | 1.14E-07 | 0.6 | 6.6 | 7.2 |
| 40117_at | MCM6 | 1.15E-07 | -1.31 | 8.09 | 6.78 |
| 36616_at | DAZAP2 | 1.16E-07 | 0.86 | 7.91 | 8.77 |
| 1323_at | UBB | 1.16E-07 | -0.61 | 11.29 | 10.68 |
| 37872_at | JRK | 1.19E-07 | -0.36 | 7.07 | 6.72 |
| 31817_at | GABRB3 | 1.19E-07 | -0.95 | 5.43 | 4.48 |
| 36170_at | ETHE1 | 1.21E-07 | 0.5 | 7.24 | 7.73 |
| 38517_at | ISGF3G | 1.21E-07 | 0.53 | 7.88 | 8.42 |
| 39314_at | ACVR2B | 1.22E-07 | -0.72 | 7.38 | 6.66 |
| 40467_at | SDHD | 1.25E-07 | 0.69 | 5.83 | 6.52 |
| 1071_at | GATA2 | 1.25E-07 | -0.68 | 7.17 | 6.49 |
| 319_g_at | H1FX | 1.29E-07 | -0.86 | 10.23 | 9.37 |
| 32398_s_at | LRP8 | 1.29E-07 | -0.42 | 5.12 | 4.7 |
| 40876_at | GYG1 | 1.30E-07 | 0.71 | 4.63 | 5.35 |
| 38559_at | hCG_40738 | 1.35E-07 | -0.37 | 5.79 | 5.42 |
| 428_s_at | B2M | 1.35E-07 | 1.33 | 10.31 | 11.65 |
| 1742_at | ERBB3 | 1.35E-07 | 0.39 | 6.71 | 7.1 |
| 31982_at | KIAA0894 | 1.35E-07 | 0.73 | 4.69 | 5.42 |
| 38293_s_at | HOXD3 | 1.36E-07 | -0.67 | 4.37 | 3.69 |
| 37073_at | EYA1 | 1.36E-07 | -1.28 | 5.45 | 4.17 |
| 36481_at | ORC4L | 1.36E-07 | -0.33 | 3.47 | 3.14 |
| 35164_at | WFS1 | 1.37E-07 | 0.48 | 6.5 | 6.97 |
| 32263_at | CCNB2 | 1.40E-07 | -1.12 | 5.28 | 4.16 |
| 39764_at | ACVR1 | 1.41E-07 | 0.68 | 4.2 | 4.88 |
| 37584_at | FANCG | 1.42E-07 | -0.43 | 7.19 | 6.76 |
| 33890_at | RGS5 | 1.44E-07 | -2.53 | 7.9 | 5.38 |
| 34327_at | HLTF | 1.46E-07 | -0.76 | 5.28 | 4.52 |
| 32366_at | CNTFR | 1.47E-07 | -1.03 | 6.97 | 5.94 |
| 31797_at | TBPL1 | 1.48E-07 | -0.72 | 5.05 | 4.33 |
| 38225_at | KCNH2 | 1.48E-07 | -0.8 | 6.43 | 5.62 |
| 38858_at | KCNH2 | 1.50E-07 | -0.57 | 7.59 | 7.03 |
| 1809_at | CDC7 | 1.53E-07 | -0.86 | 5.13 | 4.28 |
| 36104_at | UQCRH | 1.54E-07 | -0.92 | 8.54 | 7.62 |
| 40819_at | RBM8A | 1.54E-07 | -0.41 | 8.27 | 7.86 |
| 1038_s_at | IFNGR1 | 1.56E-07 | 0.89 | 5.61 | 6.5 |
| 115_at | THBS1 | 1.56E-07 | 0.96 | 6 | 6.97 |
| 33809_at | GNAI1 | 1.56E-07 | -1.22 | 5.81 | 4.59 |
| 1295_at | RELA | 1.58E-07 | 0.58 | 6.77 | 7.35 |
| 39061_at | BST2 | 1.62E-07 | 1.15 | 6.73 | 7.88 |
| 208_at | CTNNA2 | 1.65E-07 | -1.14 | 5.4 | 4.26 |
| 1826_at | RHOB | 1.66E-07 | 1.08 | 8.19 | 9.27 |
| 37940_f_at | APOBEC3C | 1.67E-07 | 0.33 | 7.87 | 8.2 |
| 671_at | SPARC | 1.73E-07 | 1.53 | 9.8 | 11.33 |
| 34417_at | DPY19L2P2 | 1.73E-07 | -1.24 | 5.77 | 4.53 |
| 32511_at | C9orf125 | 1.74E-07 | -0.58 | 7.37 | 6.79 |
| 37590_g_at | --- | 1.75E-07 | -0.64 | 6.58 | 5.94 |
| 37334_at | HNRNPA0 | 1.76E-07 | -1.09 | 7.05 | 5.96 |
| 34527_r_at | LOC157627 | 1.77E-07 | -0.53 | 7.01 | 6.48 |
| 37944_at | GCH1 | 1.77E-07 | -1.98 | 7.05 | 5.08 |
| 508_at | SUPT4H1 | 1.79E-07 | -0.74 | 7.54 | 6.81 |
| 34162_at | RBPMS | 1.80E-07 | 0.78 | 5.95 | 6.73 |
| 650_s_at | CAMK2G | 1.81E-07 | -0.47 | 6.06 | 5.59 |
| 40852_at | TDRD7 | 1.82E-07 | 0.5 | 5.23 | 5.73 |
| 40454_at | FAT | 1.83E-07 | 1.23 | 6.5 | 7.73 |
| 32535_at | FBN1 | 1.86E-07 | 1.25 | 6.02 | 7.27 |
| 32712_at | MYT1L | 1.88E-07 | -1.2 | 4.65 | 3.45 |
| 41196_at | --- | 1.89E-07 | -0.69 | 6.5 | 5.81 |
| 35334_at | GYG2 | 1.90E-07 | -0.84 | 5.62 | 4.78 |
| 37383_f_at | HLA-B | 1.91E-07 | 1.47 | 9.94 | 11.41 |
| 419_at | MKI67 | 1.92E-07 | -0.95 | 6.34 | 5.39 |
| 35008_at | PER2 | 1.92E-07 | 0.52 | 4.26 | 4.78 |
| 34408_at | RTN2 | 1.94E-07 | -0.59 | 7.38 | 6.79 |
| 39634_at | SLIT2 | 1.96E-07 | 1.19 | 7.37 | 8.56 |
| 34288_at | CXCR7 | 1.97E-07 | 0.9 | 3.2 | 4.1 |
| 37931_at | CENPB | 1.98E-07 | 0.31 | 8.47 | 8.78 |
| 32441_at | CTPS | 1.99E-07 | -0.75 | 4.61 | 3.86 |
| 35746_r_at | PCBP2 | 1.99E-07 | -0.64 | 10.58 | 9.95 |
| 35670_at | ATP1A3 | 2.02E-07 | -1.58 | 8.43 | 6.85 |
| 36150_at | PLEKHM2 | 2.03E-07 | 0.35 | 7.12 | 7.47 |
| 33227_at | IL10RB | 2.03E-07 | 0.46 | 4.8 | 5.26 |
| 34085_at | RPL38 | 2.04E-07 | -0.61 | 12.36 | 11.75 |
| 1463_at | PTPN12 | 2.05E-07 | 0.92 | 6.88 | 7.8 |
| 33373_at | FUBP1 | 2.06E-07 | -0.98 | 5.9 | 4.92 |
| 36896_s_at | ARNTL | 2.07E-07 | 0.49 | 3.39 | 3.89 |
| 1921_at | --- | 2.07E-07 | 0.47 | 3.26 | 3.73 |
| 37754_at | LGALS3BP | 2.07E-07 | 0.99 | 9.38 | 10.36 |
| 38279_at | GNAZ | 2.10E-07 | -0.93 | 6.85 | 5.93 |
| 38629_at | MAPT | 2.11E-07 | -0.97 | 7.91 | 6.94 |
| 39689_at | CST3 | 2.14E-07 | 0.62 | 6.65 | 7.27 |
| 34184_at | APC2 | 2.17E-07 | -1.26 | 9.34 | 8.07 |
| 37114_at | ZFHX3 | 2.17E-07 | -1.3 | 8.87 | 7.58 |
| 39866_at | USP22 | 2.18E-07 | -0.84 | 10.2 | 9.36 |
| 41390_at | UTRN | 2.20E-07 | 0.53 | 6.8 | 7.34 |
| 36451_at | ACVR1B | 2.20E-07 | -0.97 | 9.31 | 8.34 |
| 40081_at | PLTP | 2.21E-07 | 1.36 | 7.17 | 8.53 |
| 33936_at | GALC | 2.22E-07 | 1.15 | 4.44 | 5.59 |
| 37134_f_at | GRIN1 | 2.23E-07 | -0.61 | 8.66 | 8.05 |
| 36550_at | RIN2 | 2.24E-07 | 1.08 | 5.61 | 6.69 |
| 35314_at | NCAPD2 | 2.24E-07 | -0.73 | 5.97 | 5.23 |
| 40800_at | HN1L | 2.25E-07 | -0.32 | 5.51 | 5.19 |
| 40355_at | WDHD1 | 2.26E-07 | -0.35 | 3.68 | 3.33 |
| 40683_at | GRB14 | 2.27E-07 | 0.75 | 4.04 | 4.79 |
| 37241_at | PNMA2 | 2.37E-07 | -1.39 | 6.96 | 5.57 |
| 41536_at | ID4 | 2.41E-07 | 0.69 | 3.01 | 3.7 |
| 39715_at | --- | 2.44E-07 | -0.84 | 6.94 | 6.1 |
| 38200_at | FGD1 | 2.45E-07 | -0.42 | 6.68 | 6.26 |
| 31943_g_at | TULP3 | 2.46E-07 | 0.51 | 5.31 | 5.82 |
| 36513_at | MFAP5 | 2.46E-07 | 0.55 | 3.17 | 3.71 |
| 33899_at | ALDH9A1 | 2.46E-07 | 0.6 | 6.32 | 6.92 |
| 39374_at | GTSE1 | 2.49E-07 | -0.72 | 6.15 | 5.43 |
| 36493_at | LSP1 | 2.50E-07 | 1.16 | 5.94 | 7.09 |
| 349_g_at | KIFC1 | 2.52E-07 | -1.02 | 6.06 | 5.04 |
| 1573_at | PDGFB | 2.54E-07 | 0.48 | 7.12 | 7.6 |
| 34314_at | RRM1 | 2.54E-07 | -0.91 | 5.69 | 4.78 |
| 31903_at | SS18L1 | 2.55E-07 | -0.82 | 4.87 | 4.05 |
| 40165_at | TSPYL2 | 2.56E-07 | 0.77 | 6.81 | 7.58 |
| 34217_at | KLF7 | 2.56E-07 | -0.9 | 6.37 | 5.47 |
| 32814_at | IFIT1 | 2.60E-07 | 0.91 | 4.35 | 5.26 |
| 40492_at | FRYL | 2.63E-07 | 0.48 | 5.86 | 6.34 |
| 40451_at | POLE | 2.63E-07 | -0.52 | 7.74 | 7.22 |
| 32685_at | RALGPS1 | 2.64E-07 | -1.01 | 5.44 | 4.44 |
| 1536_at | CDC6 | 2.66E-07 | -1.1 | 4.47 | 3.37 |
| 37031_at | FAM120A | 2.66E-07 | 0.73 | 5.29 | 6.03 |
| 38736_at | WDR1 | 2.67E-07 | 0.65 | 7.33 | 7.98 |
| 37047_at | NPC1 | 2.69E-07 | 0.46 | 6.03 | 6.49 |
| 1397_at | MAP3K11 | 2.71E-07 | 0.42 | 6.22 | 6.65 |
| 38242_at | BLNK | 2.73E-07 | 0.8 | 3.78 | 4.58 |
| 41753_at | ACTN4 | 2.75E-07 | 0.63 | 8.74 | 9.37 |
| 36575_at | RGS1 | 2.78E-07 | 1.68 | 5.29 | 6.97 |
| 37811_at | CACNA2D2 | 2.79E-07 | -0.64 | 7.62 | 6.98 |
| 37158_at | SAMD14 | 2.79E-07 | -0.58 | 8.32 | 7.74 |
| 33852_at | TIA1 | 2.80E-07 | -1.1 | 7.4 | 6.29 |
| 1650_g_at | SMOX | 2.88E-07 | 0.34 | 7.74 | 8.08 |
| 38329_at | SLC25A13 | 2.89E-07 | -0.41 | 5.74 | 5.33 |
| 35686_s_at | MTCP1 | 2.92E-07 | 0.46 | 5.38 | 5.84 |
| 32156_at | PVRL2 | 2.94E-07 | 0.56 | 7.03 | 7.59 |
| 36815_at | --- | 2.95E-07 | -0.96 | 8.67 | 7.71 |
| 572_at | TTK | 2.97E-07 | -1.02 | 4.14 | 3.12 |
| 40358_at | GLI3 | 2.99E-07 | 0.37 | 4.56 | 4.93 |
| 41112_at | ZNF500 | 3.02E-07 | -0.3 | 5.59 | 5.29 |
| 36562_at | KIAA0427 | 3.03E-07 | -0.56 | 8.18 | 7.61 |
| 37420_i_at | HLA-F | 3.07E-07 | 1.36 | 7.59 | 8.95 |
| 1973_s_at | MYC | 3.09E-07 | 0.99 | 6.12 | 7.11 |
| 38988_at | WHSC1 | 3.12E-07 | -0.52 | 6.3 | 5.79 |
| 39360_at | SNX3 | 3.13E-07 | 0.73 | 8.33 | 9.05 |
| 37276_at | IQGAP2 | 3.13E-07 | 0.76 | 4.77 | 5.53 |
| 32838_at | MYH10 | 3.15E-07 | -0.81 | 7.16 | 6.35 |
| 1396_at | IGFBP5 | 3.24E-07 | 1.75 | 7.16 | 8.91 |
| 32791_at | TMEM97 | 3.32E-07 | -1.16 | 6.3 | 5.14 |
| 41405_at | SFRP4 | 3.34E-07 | 0.59 | 3.17 | 3.76 |
| 38172_at | CBR3 | 3.35E-07 | 0.37 | 4.49 | 4.86 |
| 34772_at | CORO2B | 3.35E-07 | 0.62 | 6.07 | 6.68 |
| 32573_at | SFRS9 | 3.36E-07 | -0.65 | 8.09 | 7.44 |
| 31807_at | DDX49 | 3.36E-07 | -0.38 | 7.81 | 7.42 |
| 1136_at | DTYMK | 3.37E-07 | -0.43 | 6.68 | 6.25 |
| 37976_at | VSIG4 | 3.37E-07 | 0.82 | 7.12 | 7.93 |
| 41237_at | HLA-A | 3.40E-07 | 1.61 | 9.34 | 10.95 |
| 39439_at | --- | 3.42E-07 | -0.52 | 5.41 | 4.88 |
| 33795_at | TCF20 | 3.44E-07 | -0.31 | 7.87 | 7.56 |
| 36898_r_at | PRIM2 | 3.44E-07 | -0.62 | 5.49 | 4.87 |
| 32633_at | ICA1 | 3.45E-07 | -1.31 | 7.57 | 6.26 |
| 41812_s_at | NUP210 | 3.47E-07 | -0.68 | 6.55 | 5.86 |
| 34243_i_at | L3MBTL | 3.48E-07 | -0.52 | 3.67 | 3.15 |
| 946_at | DYNLT1 | 3.49E-07 | -0.78 | 8.52 | 7.74 |
| 35171_at | SPAST | 3.50E-07 | -0.82 | 4.13 | 3.31 |
| 34683_at | CUGBP1 | 3.52E-07 | -0.72 | 6.16 | 5.44 |
| 38675_at | SNRPC | 3.53E-07 | -0.36 | 8.06 | 7.7 |
| 131_at | TAF11 | 3.54E-07 | -0.44 | 6.13 | 5.69 |
| 34027_f_at | HIST1H4J | 3.58E-07 | -0.65 | 6.7 | 6.05 |
| 37753_at | SMARCD1 | 3.59E-07 | -0.51 | 7.46 | 6.95 |
| 356_at | KIF22 | 3.63E-07 | -0.66 | 7.43 | 6.77 |
| 36410_f_at | ELAVL2 | 3.65E-07 | -1.1 | 7.22 | 6.12 |
| 1082_at | PLCG1 | 3.66E-07 | -0.5 | 5.87 | 5.37 |
| 39025_at | LOC201725 | 3.67E-07 | 0.7 | 8.77 | 9.47 |
| 910_at | TK1 | 3.70E-07 | -1.2 | 6.71 | 5.5 |
| 762_f_at | HIST1H4I | 3.73E-07 | -0.52 | 6.72 | 6.19 |
| 31952_at | LOC401725 | 3.77E-07 | -0.8 | 10.82 | 10.02 |
| 35998_at | LOC284244 | 3.79E-07 | -1.32 | 5.36 | 4.04 |
| 662_at | DHX9 | 3.82E-07 | -0.47 | 6.79 | 6.33 |
| 34337_s_at | MTF2 | 3.87E-07 | -0.82 | 6.43 | 5.62 |
| 40960_at | B4GALT1 | 3.87E-07 | 0.55 | 7.48 | 8.03 |
| 37263_at | GGH | 3.87E-07 | -0.9 | 4.8 | 3.9 |
| 32452_at | CDK3 | 3.88E-07 | -0.33 | 7.26 | 6.93 |
| 39375_g_at | GTSE1 | 3.95E-07 | -0.47 | 6.23 | 5.75 |
| 39081_at | LOC441019 | 3.98E-07 | 1.65 | 6.57 | 8.22 |
| 40119_at | CRTAP | 3.99E-07 | 0.55 | 4.53 | 5.09 |
| 37405_at | SELENBP1 | 4.00E-07 | 0.75 | 7.09 | 7.84 |
| 41008_at | KIAA0888 | 4.03E-07 | -0.76 | 4.84 | 4.09 |
| 39663_at | MAN2A1 | 4.09E-07 | 0.66 | 5.75 | 6.41 |
| 39313_at | WNK1 | 4.10E-07 | -0.8 | 5.26 | 4.46 |
| 1516_g_at | --- | 4.12E-07 | -1.26 | 7.37 | 6.11 |
| 1696_at | POLB | 4.15E-07 | -0.5 | 5.76 | 5.27 |
| 36034_at | KIAA0258 | 4.20E-07 | -0.35 | 5.73 | 5.38 |
| 39068_at | PPP2R5D | 4.21E-07 | -0.27 | 8.04 | 7.77 |
| 33844_at | RQCD1 | 4.23E-07 | -0.4 | 6.88 | 6.48 |
| 36837_at | KIF2C | 4.27E-07 | -0.8 | 5.86 | 5.05 |
| 37143_s_at | PFAS | 4.28E-07 | -0.59 | 5.81 | 5.22 |
| 32558_at | PIAS3 | 4.29E-07 | 0.34 | 6.01 | 6.35 |
| 40352_at | YPEL1 | 4.32E-07 | -0.51 | 6.77 | 6.26 |
| 34397_at | CROP | 4.41E-07 | -1.21 | 8.08 | 6.87 |
| 32065_at | CREM | 4.43E-07 | 1.02 | 4.71 | 5.73 |
| 41342_at | RANBP1 | 4.44E-07 | -0.93 | 6.3 | 5.37 |
| 39733_at | HERPUD1 | 4.51E-07 | 0.91 | 5.67 | 6.58 |
| 41251_at | ZNHIT3 | 4.60E-07 | -0.69 | 7.74 | 7.05 |
| 41393_at | ZNF195 | 4.61E-07 | -0.85 | 3.98 | 3.12 |
| 37384_at | PPM1F | 4.61E-07 | 0.36 | 7.43 | 7.79 |
| 1842_at | --- | 4.76E-07 | 0.6 | 7.12 | 7.72 |
| 41050_at | TAF5 | 4.79E-07 | -0.54 | 6.26 | 5.72 |
| 37449_i_at | GNAS | 4.81E-07 | -0.75 | 12.22 | 11.47 |
| 40331_at | MARCO | 4.87E-07 | 0.62 | 6.14 | 6.76 |
| 34866_at | LOC151162 | 4.91E-07 | -1.02 | 7.88 | 6.86 |
| 31935_s_at | DDX11 | 4.96E-07 | -0.5 | 7.61 | 7.11 |
| 1005_at | DUSP1 | 5.03E-07 | 1.62 | 8.05 | 9.67 |
| 37693_at | NUMB | 5.03E-07 | 0.52 | 3.8 | 4.32 |
| 36795_at | PSAP | 5.09E-07 | 0.97 | 10.47 | 11.44 |
| 36863_at | HMMR | 5.10E-07 | -0.6 | 5.44 | 4.84 |
| 35333_r_at | IL33 | 5.14E-07 | 0.37 | 2.43 | 2.8 |
| 38348_at | ACOX2 | 5.17E-07 | 0.4 | 3.2 | 3.61 |
| 41185_f_at | SUMO2 | 5.18E-07 | -0.67 | 10.27 | 9.6 |
| 34897_at | TNK2 | 5.25E-07 | -0.53 | 7.61 | 7.08 |
| 32178_r_at | SNAP23 | 5.25E-07 | 0.47 | 2.98 | 3.44 |
| 37963_at | ARSA | 5.27E-07 | 0.36 | 6.67 | 7.03 |
| 39369_at | MAN2B2 | 5.31E-07 | 0.68 | 6.18 | 6.86 |
| 35422_at | MAP2 | 5.34E-07 | -1.08 | 4.87 | 3.8 |
| 467_at | OSTF1 | 5.56E-07 | 0.47 | 4.81 | 5.29 |
| 34272_at | RGS4 | 5.65E-07 | -1.46 | 8.13 | 6.68 |
| 41661_at | --- | 5.68E-07 | -0.42 | 4.71 | 4.29 |
| 39165_at | ISCU | 5.76E-07 | 0.73 | 8.16 | 8.89 |
| 35169_at | COL16A1 | 5.77E-07 | 0.69 | 7.16 | 7.85 |
| 37450_r_at | GNAS | 5.84E-07 | -0.79 | 12.05 | 11.27 |
| 32694_at | RARB | 5.85E-07 | 0.56 | 4.9 | 5.46 |
| 40116_at | PFKL | 5.89E-07 | 0.34 | 7.51 | 7.85 |
| 40477_r_at | FOXK2 | 5.92E-07 | -0.76 | 4.95 | 4.19 |
| 1233_s_at | AXL | 5.97E-07 | 0.32 | 8.02 | 8.35 |
| 33402_at | KDELR3 | 5.99E-07 | 0.37 | 2.93 | 3.29 |
| 193_at | TAF9 | 6.02E-07 | -0.52 | 5.17 | 4.65 |
| 34644_at | B2M | 6.03E-07 | 1.17 | 9.7 | 10.87 |
| 37321_at | TTC1 | 6.05E-07 | 0.43 | 7.25 | 7.68 |
| 33126_at | GLT8D1 | 6.07E-07 | 0.55 | 5.61 | 6.16 |
| 37719_at | MLF2 | 6.18E-07 | -0.43 | 7.75 | 7.32 |
| 38641_at | TSC22D4 | 6.20E-07 | 0.52 | 7.6 | 8.12 |
| 33878_at | EFHD1 | 6.22E-07 | 0.46 | 6.31 | 6.77 |
| 34023_at | FCER1A | 6.23E-07 | 0.57 | 3.59 | 4.16 |
| 41290_at | NCAM1 | 6.27E-07 | -0.58 | 6.75 | 6.16 |
| 39910_at | 2-Mar | 6.30E-07 | 0.4 | 5.43 | 5.83 |
| 37641_at | IFI44 | 6.31E-07 | 1.04 | 5.84 | 6.88 |
| 33785_at | BAI2 | 6.35E-07 | -0.63 | 8.85 | 8.22 |
| 41600_at | PA2G4 | 6.45E-07 | -0.56 | 7.07 | 6.51 |
| 35299_at | MKNK1 | 6.51E-07 | 0.56 | 5.65 | 6.21 |
| 34218_at | SPEG | 6.59E-07 | -0.6 | 6.78 | 6.18 |
| 38724_at | KIAA0515 | 6.69E-07 | -0.8 | 8.27 | 7.47 |
| 37458_at | CDC45L | 6.70E-07 | -0.57 | 6.56 | 5.99 |
| 37231_at | DLG7 | 6.71E-07 | -0.62 | 4.48 | 3.86 |
| 38650_at | IGFBP5 | 6.76E-07 | 1.95 | 7.66 | 9.61 |
| 35349_at | COPS3 | 6.78E-07 | -0.47 | 6.83 | 6.36 |
| 34774_at | PPT1 | 6.80E-07 | 0.81 | 6.45 | 7.26 |
| 41474_at | KIF2A | 6.85E-07 | -0.87 | 6.54 | 5.67 |
| 37668_at | C1QBP | 6.88E-07 | -0.91 | 7.35 | 6.44 |
| 37184_at | STX1A | 6.90E-07 | -0.5 | 8.32 | 7.83 |
| 38511_at | ZNF529 | 6.92E-07 | -1.04 | 4.21 | 3.16 |
| 37499_at | KIAA0408 | 7.02E-07 | -0.8 | 4.67 | 3.87 |
| 38717_at | METTL7A | 7.08E-07 | 1.49 | 5.37 | 6.86 |
| 41483_s_at | JUND | 7.13E-07 | 0.7 | 10.67 | 11.37 |
| 38883_at | --- | 7.13E-07 | -0.39 | 7.17 | 6.77 |
| 943_at | RUNX1 | 7.14E-07 | 0.79 | 5.78 | 6.56 |
| 37448_s_at | GNAS | 7.15E-07 | -0.75 | 11.28 | 10.54 |
| 33222_at | FZD7 | 7.23E-07 | 0.71 | 4.45 | 5.16 |
| 38237_at | GGTLA1 | 7.32E-07 | 0.51 | 8.32 | 8.82 |
| 34304_s_at | SAT1 | 7.34E-07 | 1.2 | 6.02 | 7.22 |
| 34216_at | KLF7 | 7.34E-07 | -0.99 | 7.52 | 6.53 |
| 36371_at | MFAP3 | 7.41E-07 | -0.51 | 5.41 | 4.9 |
| 41168_at | TAPBP | 7.52E-07 | 0.96 | 8.02 | 8.98 |
| 39062_at | CTSA | 7.53E-07 | 0.71 | 7.46 | 8.17 |
| 1276_g_at | RBPMS | 7.61E-07 | 0.56 | 7.26 | 7.82 |
| 1478_at | ITK | 7.63E-07 | 1.1 | 3.61 | 4.71 |
| 39445_at | B4GALT3 | 7.66E-07 | -0.24 | 8.3 | 8.06 |
| 40187_at | --- | 7.80E-07 | -0.43 | 3.99 | 3.56 |
| 33457_at | C3orf63 | 7.82E-07 | 0.62 | 5.86 | 6.47 |
| 34656_at | MPP2 | 7.86E-07 | -0.76 | 7.02 | 6.26 |
| 996_at | FGF1 | 8.02E-07 | 1 | 3.94 | 4.94 |
| 39294_at | NR2F1 | 8.04E-07 | 0.91 | 6.71 | 7.62 |
| 2094_s_at | FOS | 8.09E-07 | 1.67 | 8.75 | 10.42 |
| 35175_f_at | EEF1A2 | 8.13E-07 | -0.7 | 10.69 | 9.99 |
| 35506_s_at | CST1 | 8.14E-07 | 0.65 | 4.32 | 4.97 |
| 798_at | PRIM1 | 8.14E-07 | -0.63 | 5.1 | 4.47 |
| 2056_at | FGFR1 | 8.16E-07 | 0.38 | 7.22 | 7.6 |
| 763_at | GMFB | 8.21E-07 | 0.7 | 6.34 | 7.04 |
| 1840_g_at | --- | 8.25E-07 | -0.81 | 9.67 | 8.86 |
| 38953_at | TRAIP | 8.27E-07 | -0.44 | 5.97 | 5.53 |
| 183_at | MAP2 | 8.33E-07 | -1.18 | 4.59 | 3.4 |
| 503_at | POLR2L | 8.40E-07 | 0.59 | 8.53 | 9.12 |
| 40571_at | MYO5A | 8.45E-07 | 0.7 | 3.85 | 4.54 |
| 41395_at | CHST1 | 8.48E-07 | -1.06 | 8.3 | 7.24 |
| 33033_at | ATP6V1G2 | 8.52E-07 | -0.46 | 7.04 | 6.58 |
| 1868_g_at | CFLAR | 8.60E-07 | 0.69 | 4.19 | 4.88 |
| 33862_at | PPAP2B | 8.72E-07 | 0.86 | 7.81 | 8.67 |
| 36020_at | KIAA1641 | 8.76E-07 | -0.56 | 5.08 | 4.52 |
| 39713_at | JARID1B | 8.84E-07 | -0.62 | 6.28 | 5.67 |
| 39571_at | CAMSAP1 | 8.89E-07 | -0.52 | 5.38 | 4.86 |
| 903_at | PPP2R5A | 8.95E-07 | 0.7 | 5.22 | 5.93 |
| 40652_at | PASK | 8.97E-07 | -0.6 | 5.37 | 4.77 |
| 32210_at | PGM1 | 9.02E-07 | 0.66 | 6.06 | 6.72 |
| 1178_at | --- | 9.02E-07 | -1.02 | 4.29 | 3.27 |
| 36845_at | MORC3 | 9.02E-07 | 0.6 | 7.3 | 7.9 |
| 39540_at | ZBTB7A | 9.27E-07 | 0.45 | 6.21 | 6.66 |
| 40678_at | ST8SIA1 | 9.28E-07 | -0.56 | 4.11 | 3.55 |
| 40985_g_at | 76P | 9.51E-07 | -0.6 | 5.84 | 5.24 |
| 32924_at | MMP24 | 9.63E-07 | -0.37 | 7.27 | 6.9 |
| 38761_s_at | FKBP9 | 9.72E-07 | 0.65 | 6.15 | 6.8 |
| 33433_at | FAM98A | 9.81E-07 | 0.46 | 5.84 | 6.3 |
| 33576_at | SLITRK5 | 9.83E-07 | 0.85 | 6.07 | 6.92 |
| 36611_at | ACP1 | 9.83E-07 | -0.59 | 8.26 | 7.67 |
| 2062_at | IGFBP7 | 9.84E-07 | 1.42 | 9.34 | 10.76 |
| 1427_g_at | SLA | 1.00E-06 | 0.68 | 3.63 | 4.31 |
| 34843_at | ZNF516 | 1.00E-06 | 0.5 | 5.28 | 5.78 |
| 33425_at | TRIM28 | 1.00E-06 | -0.73 | 9.7 | 8.97 |
| 1768_s_at | CSK | 1.01E-06 | -0.31 | 8.04 | 7.73 |
| 39045_at | ADIPOR2 | 1.01E-06 | 0.54 | 8.1 | 8.63 |
| 34008_at | REM1 | 1.02E-06 | 0.28 | 7.06 | 7.34 |
| 1161_at | HSP90AB1 | 1.04E-06 | -0.63 | 10.85 | 10.22 |
| 36021_at | LEF1 | 1.05E-06 | 0.73 | 5.61 | 6.34 |
| 1700_at | BBC3 | 1.06E-06 | -0.27 | 8.01 | 7.73 |
| 35745_f_at | PCBP2 | 1.06E-06 | -0.66 | 9.71 | 9.04 |
| 41083_at | ZNF428 | 1.06E-06 | -0.69 | 7.27 | 6.58 |
| 37710_at | MEF2C | 1.07E-06 | 0.71 | 3.51 | 4.23 |
| 36779_at | FABP6 | 1.08E-06 | -0.89 | 6.94 | 6.05 |
| 38545_at | INHBB | 1.09E-06 | 1.52 | 6.14 | 7.65 |
| 33849_at | PBEF1 | 1.10E-06 | 0.86 | 4.18 | 5.04 |
| 37457_at | LRRTM2 | 1.10E-06 | -1.01 | 5.46 | 4.45 |
| 34947_at | APOBEC3G | 1.10E-06 | 0.34 | 4.93 | 5.27 |
| 36149_at | DPYSL3 | 1.11E-06 | -1.57 | 8.71 | 7.14 |
| 38356_at | FST | 1.12E-06 | 0.47 | 6 | 6.48 |
| 38746_at | ITGB4 | 1.14E-06 | 0.34 | 6.74 | 7.09 |
| 35494_at | SLC2A3P1 | 1.14E-06 | -0.88 | 5.26 | 4.38 |
| 38908_s_at | REV3L | 1.15E-06 | -1.15 | 6.95 | 5.8 |
| 35936_g_at | CHKB | 1.17E-06 | -0.78 | 6.9 | 6.12 |
| 32857_at | RASSF3 | 1.19E-06 | 0.65 | 5.13 | 5.78 |
| 41626_at | TIMELESS | 1.19E-06 | -0.55 | 4.75 | 4.2 |
| 39611_at | --- | 1.20E-06 | -0.75 | 7.74 | 6.99 |
| 37306_at | CYFIP1 | 1.20E-06 | 0.82 | 6.71 | 7.53 |
| 36987_at | LMNB2 | 1.21E-06 | -0.79 | 8.16 | 7.37 |
| 34674_at | S100A1 | 1.21E-06 | 0.41 | 5.1 | 5.51 |
| 37213_at | DNASE1L1 | 1.21E-06 | 0.38 | 5.31 | 5.69 |
| 719_g_at | HTRA1 | 1.23E-06 | 1.5 | 6.82 | 8.32 |
| 32067_at | CREM | 1.23E-06 | 1.26 | 4.94 | 6.2 |
| 37267_at | THOP1 | 1.24E-06 | -0.31 | 7.46 | 7.16 |
| 33982_f_at | ZNF43 | 1.24E-06 | -0.87 | 5.23 | 4.35 |
| 37528_at | ASPH | 1.26E-06 | 0.31 | 5.18 | 5.49 |
| 41184_s_at | PSMB8 | 1.26E-06 | 0.67 | 6.35 | 7.01 |
| 32680_at | TNIK | 1.27E-06 | -0.46 | 5.88 | 5.43 |
| 31802_at | KIAA0226 | 1.28E-06 | 0.54 | 7.58 | 8.12 |
| 41113_at | ZNF500 | 1.29E-06 | -0.37 | 6.29 | 5.93 |
| 33346_r_at | TUBG1 | 1.30E-06 | -0.68 | 7.71 | 7.03 |
| 425_at | IFI27 | 1.30E-06 | 0.99 | 7.84 | 8.83 |
| 36607_at | NAGA | 1.31E-06 | 0.34 | 5.69 | 6.02 |
| 35342_at | PTPLB | 1.32E-06 | 0.63 | 5.75 | 6.38 |
| 39269_at | RFC3 | 1.33E-06 | -0.75 | 4.38 | 3.63 |
| 40244_s_at | MPPE1 | 1.33E-06 | 0.39 | 6.49 | 6.88 |
| 36555_at | SNCG | 1.34E-06 | 1.18 | 8.26 | 9.44 |
| 1649_at | SMOX | 1.34E-06 | 0.43 | 6.01 | 6.44 |
| 40631_at | TOB1 | 1.35E-06 | 1.02 | 6.16 | 7.17 |
| 37135_f_at | GRIN1 | 1.35E-06 | -0.73 | 8.09 | 7.36 |
| 41192_at | CCBL2 | 1.36E-06 | 0.45 | 4.22 | 4.67 |
| 35147_at | MCF2L | 1.36E-06 | -1.04 | 8.42 | 7.38 |
| 36638_at | CTGF | 1.36E-06 | 2.1 | 6.89 | 8.99 |
| 36236_at | --- | 1.37E-06 | -0.5 | 5.66 | 5.16 |
| 35284_f_at | EXDL2 | 1.41E-06 | -0.34 | 4.11 | 3.77 |
| 36336_s_at | SBNO2 | 1.42E-06 | 0.59 | 6.41 | 7 |
| 1915_s_at | FOS | 1.42E-06 | 2.24 | 7.88 | 10.13 |
| 38847_at | MELK | 1.42E-06 | -0.5 | 2.85 | 2.35 |
| 38968_at | SH3BP5 | 1.43E-06 | 1.09 | 7.7 | 8.79 |
| 36557_at | CACNB1 | 1.46E-06 | -0.63 | 8.11 | 7.48 |
| 35659_at | IL10RA | 1.46E-06 | 0.31 | 7.58 | 7.9 |
| 37852_at | --- | 1.46E-06 | -0.62 | 5.52 | 4.9 |
| 35657_at | TARBP2 | 1.47E-06 | -0.36 | 7.45 | 7.09 |
| 36591_at | TUBA4A | 1.48E-06 | 1.07 | 7.16 | 8.23 |
| 41236_at | SMCR7L | 1.49E-06 | -0.37 | 7.03 | 6.66 |
| 2031_s_at | CDKN1A | 1.51E-06 | 0.92 | 7.47 | 8.39 |
| 38422_s_at | FHL2 | 1.51E-06 | 1.09 | 5.17 | 6.26 |
| 38032_at | SV2A | 1.52E-06 | -0.89 | 8.37 | 7.48 |
| 38159_at | --- | 1.53E-06 | -1.1 | 3.68 | 2.58 |
| 33875_at | ATP6V0E1 | 1.54E-06 | 0.89 | 6.42 | 7.31 |
| 36739_at | PDK4 | 1.55E-06 | 0.28 | 3.42 | 3.7 |
| 38171_at | WDR68 | 1.56E-06 | -0.6 | 7.14 | 6.54 |
| 40966_at | STK39 | 1.57E-06 | -0.79 | 6.88 | 6.09 |
| 1173_g_at | --- | 1.58E-06 | 1.28 | 9.04 | 10.32 |
| 35768_at | RNF40 | 1.59E-06 | -0.29 | 7.6 | 7.31 |
| 37364_at | PHB2 | 1.59E-06 | -0.71 | 9.04 | 8.33 |
| 34827_at | ULK1 | 1.60E-06 | -0.54 | 8.16 | 7.61 |
| 36215_at | PRKACB | 1.62E-06 | -1.26 | 7.16 | 5.91 |
| 38140_at | EXTL1 | 1.63E-06 | 0.42 | 3.85 | 4.27 |
| 36299_at | CALCA | 1.64E-06 | 1.07 | 5.67 | 6.74 |
| 38233_at | HOMER3 | 1.66E-06 | 0.42 | 5.67 | 6.1 |
| 924_s_at | PPP2CB | 1.67E-06 | 0.58 | 7.9 | 8.49 |
| 38094_at | HNRPAB | 1.68E-06 | -0.6 | 8.4 | 7.8 |
| 35397_at | RAD51AP1 | 1.69E-06 | -0.49 | 4.31 | 3.82 |
| 36649_at | EXOSC10 | 1.69E-06 | 0.7 | 5.94 | 6.64 |
| 31503_at | --- | 1.70E-06 | -0.39 | 8.16 | 7.77 |
| 37990_at | RYR1 | 1.70E-06 | 0.63 | 5.24 | 5.87 |
| 31593_at | LOC652147 | 1.70E-06 | -0.47 | 7.45 | 6.98 |
| 38973_at | HIC2 | 1.70E-06 | -0.62 | 7.69 | 7.06 |
| 37975_at | CYBB | 1.72E-06 | 0.67 | 5.89 | 6.56 |
| 33447_at | MRCL3 | 1.73E-06 | 1.13 | 6.74 | 7.87 |
| 41758_at | TMEM184B | 1.74E-06 | 0.7 | 7.46 | 8.17 |
| 35666_at | SEMA3F | 1.76E-06 | -0.32 | 7.79 | 7.47 |
| 34845_at | SAMM50 | 1.77E-06 | -0.45 | 7.02 | 6.57 |
| 33679_f_at | TUBB2C | 1.78E-06 | -0.93 | 11.14 | 10.2 |
| 35759_at | CCT2 | 1.82E-06 | -1.26 | 9.91 | 8.65 |
| 31520_at | CBX2 | 1.82E-06 | -0.59 | 5.9 | 5.32 |
| 1426_at | SLA | 1.82E-06 | 0.6 | 6.44 | 7.05 |
| 40508_at | GSTA4 | 1.89E-06 | -0.82 | 8.04 | 7.22 |
| 39213_at | PAK7 | 1.90E-06 | -0.71 | 3.3 | 2.59 |
| 39056_at | PAICS | 1.90E-06 | -1.21 | 5.53 | 4.32 |
| 41735_at | DENND3 | 1.91E-06 | 0.48 | 4.54 | 5.01 |
| 40222_s_at | --- | 1.91E-06 | -0.47 | 7.56 | 7.09 |
| 36635_at | ATP11B | 1.92E-06 | 0.53 | 6.47 | 7 |
| 40701_at | USP13 | 1.92E-06 | -0.44 | 6.13 | 5.69 |
| 39100_at | SPOCK1 | 1.93E-06 | -0.78 | 6.2 | 5.42 |
| 36411_s_at | ELAVL2 | 1.93E-06 | -0.76 | 7.65 | 6.89 |
| 1706_at | ARAF | 1.96E-06 | 0.41 | 7.32 | 7.72 |
| 37692_at | DBI | 2.01E-06 | 0.68 | 6 | 6.68 |
| 33337_at | DEGS1 | 2.02E-06 | 0.65 | 7.31 | 7.95 |
| 38049_g_at | RBPMS | 2.03E-06 | 0.61 | 6.28 | 6.89 |
| 35139_at | ZNF248 | 2.04E-06 | -0.68 | 6.23 | 5.55 |
| 36255_at | ADORA3 | 2.06E-06 | 0.44 | 3.74 | 4.18 |
| 37137_at | GZMB | 2.08E-06 | 0.48 | 3.45 | 3.93 |
| 41187_at | MRLC2 | 2.08E-06 | 0.73 | 7.83 | 8.57 |
| 1916_s_at | FOS | 2.10E-06 | 2.37 | 7.29 | 9.66 |
| 34904_at | GRIK5 | 2.11E-06 | -0.9 | 6.78 | 5.88 |
| 1279_s_at | --- | 2.13E-06 | -0.27 | 6.46 | 6.19 |
| 32076_at | RCAN2 | 2.15E-06 | 0.88 | 5.21 | 6.09 |
| 37170_at | BMP2K | 2.16E-06 | 0.49 | 4.18 | 4.67 |
| 32268_at | MAGI1 | 2.17E-06 | -0.7 | 4.62 | 3.93 |
| 35970_g_at | MPHOSPH9 | 2.19E-06 | -0.89 | 5.58 | 4.69 |
| 41829_at | LARP1 | 2.19E-06 | -0.55 | 8.26 | 7.71 |
| 1839_at | --- | 2.22E-06 | -0.88 | 8.39 | 7.51 |
| 922_at | PPP2R1A | 2.26E-06 | -0.53 | 9.44 | 8.91 |
| 1211_s_at | CRADD | 2.27E-06 | 0.67 | 5.82 | 6.49 |
| 41300_s_at | ITM2B | 2.27E-06 | 0.85 | 8.24 | 9.09 |
| 33074_g_at | PAK3 | 2.28E-06 | -1.21 | 5.72 | 4.52 |
| 33898_at | MCRS1 | 2.28E-06 | -0.23 | 7.35 | 7.12 |
| 35985_at | AKAP2 | 2.29E-06 | 0.95 | 5.81 | 6.75 |
| 848_at | TRAF1 | 2.30E-06 | 0.32 | 7.09 | 7.4 |
| 34821_at | CCDC28A | 2.32E-06 | 0.55 | 5.7 | 6.26 |
| 36505_at | CASQ2 | 2.32E-06 | 0.26 | 4.04 | 4.3 |
| 35130_at | GSR | 2.32E-06 | -0.36 | 5.67 | 5.31 |
| 35841_at | POLR2L | 2.33E-06 | 0.52 | 7.79 | 8.31 |
| 32753_at | SF3B3 | 2.34E-06 | -0.58 | 5.47 | 4.88 |
| 36497_at | AHNAK2 | 2.36E-06 | 0.92 | 5.33 | 6.25 |
| 37228_at | PLK1 | 2.37E-06 | -0.44 | 7.26 | 6.81 |
| 718_at | HTRA1 | 2.37E-06 | 1.14 | 6.89 | 8.02 |
| 31949_at | RASGRF1 | 2.42E-06 | 0.45 | 3.2 | 3.65 |
| 37038_at | ABCD3 | 2.42E-06 | 0.67 | 5.92 | 6.59 |
| 33345_at | KIF3C | 2.43E-06 | -0.75 | 8.26 | 7.51 |
| 35214_at | UGDH | 2.44E-06 | 0.59 | 7.04 | 7.63 |
| 37781_at | NRXN2 | 2.47E-06 | -0.56 | 8.14 | 7.58 |
| 34673_r_at | HDGFRP3 | 2.47E-06 | -0.29 | 3.66 | 3.37 |
| 34460_at | BZRAP1 | 2.48E-06 | -1.05 | 7.14 | 6.09 |
| 41853_at | PRPSAP2 | 2.49E-06 | -0.61 | 5.05 | 4.44 |
| 34028_at | GPR19 | 2.50E-06 | -0.46 | 3.98 | 3.52 |
| 32643_at | GBE1 | 2.52E-06 | 0.8 | 6.23 | 7.03 |
| 39438_at | CREBL2 | 2.53E-06 | 0.64 | 4.34 | 4.98 |
| 1313_at | PSMB7 | 2.56E-06 | -0.73 | 7.26 | 6.53 |
| 1440_s_at | FAS | 2.57E-06 | 0.48 | 3.43 | 3.91 |
| 36317_at | CORO2A | 2.58E-06 | -0.51 | 5.58 | 5.07 |
| 41729_at | ARIH1 | 2.59E-06 | 0.49 | 5.72 | 6.2 |
| 40708_at | MPHOSPH9 | 2.61E-06 | -0.53 | 4.14 | 3.61 |
| 35638_at | RUNX1T1 | 2.62E-06 | -1.05 | 7.75 | 6.71 |
| 34811_at | ATP5G3 | 2.64E-06 | -0.55 | 8.15 | 7.59 |
| 36979_at | SLC2A3 | 2.65E-06 | 1.22 | 7.25 | 8.47 |
| 40638_at | SFPQ | 2.66E-06 | -0.73 | 6.11 | 5.38 |
| 40136_at | TBC1D9B | 2.67E-06 | 0.33 | 7.31 | 7.64 |
| 1729_at | TRADD | 2.68E-06 | 0.29 | 7.01 | 7.29 |
| 40628_at | AAK1 | 2.70E-06 | -0.35 | 4.17 | 3.82 |
| 1895_at | JUN | 2.70E-06 | 0.71 | 5.87 | 6.58 |
| 34242_at | L3MBTL | 2.72E-06 | -0.36 | 6.43 | 6.08 |
| 34328_s_at | HLTF | 2.74E-06 | -0.66 | 3.33 | 2.67 |
| 371_at | IRF3 | 2.75E-06 | 0.33 | 7.51 | 7.84 |
| 34521_at | MAP3K13 | 2.77E-06 | -0.32 | 5.1 | 4.79 |
| 39060_at | PSMB7 | 2.78E-06 | -0.54 | 7.46 | 6.91 |
| 1206_at | CDK5 | 2.78E-06 | -0.48 | 7.03 | 6.55 |
| 41475_at | NINJ1 | 2.79E-06 | 0.32 | 6.84 | 7.16 |
| 1544_at | BLM | 2.79E-06 | -0.73 | 5.48 | 4.76 |
| 37935_at | PRPF4 | 2.81E-06 | -0.48 | 6.03 | 5.55 |
| 743_at | NAP1L3 | 2.81E-06 | -0.9 | 4.92 | 4.02 |
| 35341_at | TRIM38 | 2.81E-06 | 0.52 | 5.64 | 6.16 |
| 36785_at | HSPB1 | 2.81E-06 | 1.19 | 9.92 | 11.1 |
| 35679_s_at | DPP6 | 2.85E-06 | -1.49 | 6.65 | 5.15 |
| 41013_at | C10orf72 | 2.86E-06 | 0.58 | 6.04 | 6.62 |
| 41705_at | RFNG | 2.87E-06 | -0.35 | 6.78 | 6.43 |
| 506_s_at | STAT5A | 2.89E-06 | 0.41 | 6.75 | 7.15 |
| 34238_at | IGSF1 | 2.92E-06 | 0.56 | 5.61 | 6.17 |
| 34809_at | KIAA0999 | 2.93E-06 | 0.28 | 3.85 | 4.13 |
| 1803_at | CDC2 | 2.94E-06 | -0.67 | 6.18 | 5.52 |
| 34265_at | SCG5 | 2.96E-06 | -1.44 | 7.31 | 5.87 |
| 38413_at | DAD1 | 3.02E-06 | 0.62 | 7.32 | 7.94 |
| 35323_at | EIF3B | 3.02E-06 | -0.44 | 8.76 | 8.32 |
| 38669_at | SLK | 3.10E-06 | 0.55 | 5.75 | 6.3 |
| 32683_at | TMPO | 3.11E-06 | -0.51 | 3.08 | 2.57 |
| 41136_s_at | APP | 3.11E-06 | 0.88 | 7.23 | 8.11 |
| 32758_g_at | RAE1 | 3.14E-06 | -0.39 | 6.73 | 6.34 |
| 34935_at | FMO2 | 3.14E-06 | 0.3 | 3.03 | 3.33 |
| 33865_at | ZMYND11 | 3.16E-06 | 0.76 | 5.7 | 6.47 |
| 34726_at | CACNB3 | 3.20E-06 | -0.83 | 7.39 | 6.56 |
| 631_g_at | DCTD | 3.22E-06 | 0.69 | 6.76 | 7.45 |
| 35674_at | PADI2 | 3.22E-06 | 0.3 | 5.16 | 5.46 |
| 39968_at | LTC4S | 3.22E-06 | 0.24 | 7.21 | 7.46 |
| 41738_at | CALD1 | 3.25E-06 | 1.21 | 5.6 | 6.81 |
| 39421_at | RUNX1 | 3.27E-06 | 0.66 | 6.04 | 6.69 |
| 39860_at | FUBP1 | 3.29E-06 | -0.59 | 6.99 | 6.4 |
| 895_at | MIF | 3.34E-06 | -0.64 | 10.65 | 10.01 |
| 31523_f_at | HIST1H2BE | 3.37E-06 | -0.46 | 6.01 | 5.55 |
| 35983_at | WDR18 | 3.37E-06 | -0.52 | 8.44 | 7.92 |
| 31838_at | C12orf24 | 3.42E-06 | -0.61 | 3.42 | 2.81 |
| 1764_s_at | MAZ | 3.42E-06 | -0.98 | 6.7 | 5.72 |
| 35109_at | NFASC | 3.42E-06 | -0.41 | 6.69 | 6.28 |
| 865_at | RPS6KA3 | 3.43E-06 | 0.32 | 5.23 | 5.56 |
| 37221_at | PRKAR2B | 3.44E-06 | -0.93 | 4.99 | 4.06 |
| 38710_at | OTUB1 | 3.46E-06 | -0.35 | 9.46 | 9.11 |
| 32151_at | RANGAP1 | 3.46E-06 | -0.38 | 8.15 | 7.76 |
| 41277_at | SAP18 | 3.46E-06 | 0.45 | 4.66 | 5.11 |
| 1994_at | ATF2 | 3.46E-06 | -0.6 | 5.39 | 4.79 |
| 34687_at | MRS2L | 3.47E-06 | -0.25 | 4.27 | 4.02 |
| 37503_at | KIAA0984 | 3.49E-06 | -0.56 | 4.04 | 3.48 |
| 34852_g_at | AURKA | 3.51E-06 | -0.62 | 5.21 | 4.59 |
| 32291_at | UNC13A | 3.55E-06 | -0.5 | 4.57 | 4.07 |
| 36098_at | SFRS1 | 3.57E-06 | -0.96 | 7.22 | 6.27 |
| 40414_at | VARS | 3.58E-06 | -0.4 | 8.19 | 7.79 |
| 36850_at | --- | 3.58E-06 | -0.39 | 5.18 | 4.78 |
| 37873_g_at | JRK | 3.59E-06 | -0.43 | 5.87 | 5.43 |
| 35303_at | INSIG1 | 3.59E-06 | 0.92 | 5.32 | 6.25 |
| 1565_s_at | GRB2 | 3.60E-06 | -0.58 | 6.95 | 6.37 |
| 32126_at | FGF7 | 3.61E-06 | 0.39 | 2.64 | 3.03 |
| 409_at | YWHAQ | 3.63E-06 | -0.74 | 9.88 | 9.14 |
| 37700_at | BLMH | 3.63E-06 | -0.65 | 6.96 | 6.31 |
| 35221_at | PURA | 3.64E-06 | 0.85 | 6.76 | 7.6 |
| 306_s_at | HMGN1 | 3.65E-06 | -0.81 | 9.15 | 8.34 |
| 41781_at | PPFIA1 | 3.66E-06 | -0.28 | 4.98 | 4.7 |
| 368_at | TPBG | 3.67E-06 | 0.46 | 4.7 | 5.16 |
| 40360_at | SLC10A3 | 3.67E-06 | 0.29 | 7.29 | 7.57 |
| 33324_s_at | CDC2 | 3.69E-06 | -0.51 | 3.78 | 3.26 |
| 40771_at | MSN | 3.70E-06 | 1.38 | 8.51 | 9.89 |
| 40867_at | PPP2R1A | 3.72E-06 | -0.51 | 8.67 | 8.16 |
| 41604_at | ANKRD46 | 3.74E-06 | -0.81 | 6.73 | 5.92 |
| 36783_f_at | ZNF117 | 3.78E-06 | -0.88 | 6.89 | 6.02 |
| 40041_at | NDC80 | 3.79E-06 | -1.16 | 4.54 | 3.38 |
| 429_f_at | TUBB2A | 3.79E-06 | -1 | 11.05 | 10.05 |
| 31522_f_at | HIST1H2BF | 3.79E-06 | -0.54 | 5.88 | 5.34 |
| 1274_s_at | CDC34 | 3.80E-06 | -0.39 | 6.85 | 6.46 |
| 37465_at | TPPP | 3.83E-06 | -0.41 | 6.96 | 6.55 |
| 38429_at | FASN | 3.84E-06 | -0.77 | 9.05 | 8.27 |
| 38527_at | NONO | 3.85E-06 | -0.75 | 9.73 | 8.98 |
| 39019_at | LAPTM4A | 3.86E-06 | 0.89 | 8.33 | 9.22 |
| 1772_s_at | FNTA | 3.86E-06 | 0.48 | 5.83 | 6.31 |
| 31731_at | CBX4 | 3.87E-06 | -0.51 | 6.64 | 6.13 |
| 544_at | NFKB2 | 3.89E-06 | 0.7 | 5.5 | 6.2 |
| 37144_at | PIAS4 | 3.90E-06 | -0.32 | 5.08 | 4.76 |
| 36049_at | RAB6B | 3.94E-06 | -1.12 | 8.13 | 7.01 |
| 34864_at | C2orf24 | 3.97E-06 | 0.24 | 8.6 | 8.84 |
| 791_g_at | NGFB | 4.00E-06 | 0.55 | 4.44 | 4.98 |
| 38110_at | SDCBP | 4.03E-06 | 0.95 | 6.38 | 7.33 |
| 41289_at | NCAM1 | 4.04E-06 | -1.11 | 8.8 | 7.69 |
| 34192_at | VPS13B | 4.04E-06 | 0.61 | 5.39 | 6 |
| 39434_at | FAM21A | 4.04E-06 | 0.65 | 5.84 | 6.49 |
| 38800_at | STMN2 | 4.05E-06 | -1.79 | 11.34 | 9.54 |
| 38576_at | HIST1H2BD | 4.07E-06 | -0.61 | 5.03 | 4.42 |
| 1000_at | MAPK3 | 4.10E-06 | 0.41 | 7.69 | 8.1 |
| 40437_at | TMEM87A | 4.15E-06 | 0.54 | 6.75 | 7.29 |
| 39420_at | DDIT3 | 4.17E-06 | 0.5 | 7.71 | 8.2 |
| 1036_at | IL15 | 4.27E-06 | 0.37 | 3.01 | 3.39 |
| 33226_at | JMJD2B | 4.27E-06 | -0.33 | 8.39 | 8.06 |
| 39674_r_at | ECM2 | 4.27E-06 | 0.48 | 5.27 | 5.75 |
| 404_at | IL4R | 4.28E-06 | 0.69 | 5.8 | 6.49 |
| 41447_at | CHSY1 | 4.31E-06 | 0.88 | 6.16 | 7.03 |
| 33873_at | VPS72 | 4.36E-06 | -0.43 | 8.38 | 7.95 |
| 35433_s_at | APC | 4.40E-06 | -0.32 | 4.3 | 3.97 |
| 2047_s_at | JUP | 4.45E-06 | -0.51 | 8.37 | 7.85 |
| 41479_s_at | RAD51C | 4.46E-06 | -0.39 | 4.39 | 4 |
| 35546_at | SMC1A | 4.46E-06 | -0.7 | 4.6 | 3.9 |
| 1726_at | --- | 4.46E-06 | -0.37 | 6.9 | 6.53 |
| 39811_at | C19orf50 | 4.50E-06 | 0.46 | 7.19 | 7.65 |
| 40846_g_at | ILF3 | 4.51E-06 | -0.66 | 8.33 | 7.67 |
| 37446_at | GPRASP1 | 4.51E-06 | -0.9 | 6.07 | 5.17 |
| 37214_g_at | DNASE1L1 | 4.52E-06 | 0.42 | 5.08 | 5.49 |
| 933_f_at | ZNF91 | 4.54E-06 | -0.85 | 6.74 | 5.89 |
| 40070_at | RBM10 | 4.58E-06 | -0.39 | 7.52 | 7.14 |
| 41091_at | BPTF | 4.64E-06 | -0.99 | 7.58 | 6.59 |
| 1195_s_at | ITGB1BP1 | 4.67E-06 | 0.5 | 4.89 | 5.38 |
| 32248_at | WDR82 | 4.68E-06 | -0.43 | 7.27 | 6.84 |
| 40053_at | TXNDC4 | 4.76E-06 | -0.35 | 4.26 | 3.91 |
| 39537_at | KLHDC3 | 4.77E-06 | -0.62 | 8.25 | 7.62 |
| 32290_at | SCAMP1 | 4.78E-06 | -0.37 | 5.88 | 5.51 |
| 38768_at | HADH | 4.81E-06 | 0.71 | 5.7 | 6.4 |
| 36553_at | ASMTL | 4.82E-06 | 0.76 | 5.99 | 6.75 |
| 37908_at | GNG11 | 4.87E-06 | 1.25 | 5.12 | 6.38 |
| 1085_s_at | PLCG2 | 4.89E-06 | 0.47 | 6.74 | 7.21 |
| 33909_at | SF3B4 | 4.89E-06 | -0.33 | 7.82 | 7.49 |
| 34212_at | AGTPBP1 | 5.01E-06 | -0.48 | 4.6 | 4.12 |
| 40023_at | BDNF | 5.07E-06 | 0.38 | 2.56 | 2.94 |
| 1642_at | MTA1 | 5.07E-06 | -0.59 | 8.21 | 7.62 |
| 40789_at | AK2 | 5.09E-06 | 0.35 | 5.35 | 5.7 |
| 41264_at | RBM4B | 5.12E-06 | -0.44 | 7.41 | 6.97 |
| 33188_at | PPIL2 | 5.13E-06 | -0.5 | 7.18 | 6.67 |
| 37838_at | F12 | 5.16E-06 | -0.55 | 7.3 | 6.76 |
| 36131_at | CLIC1 | 5.19E-06 | 1.02 | 7.97 | 8.99 |
| 41866_s_at | DTNB | 5.24E-06 | -0.31 | 7.82 | 7.5 |
| 1054_at | RFC4 | 5.24E-06 | -0.56 | 4.51 | 3.95 |
| 1009_at | HINT1 | 5.26E-06 | -0.59 | 9.16 | 8.57 |
| 39254_at | RAI14 | 5.28E-06 | 0.36 | 6 | 6.37 |
| 34395_at | ZBTB5 | 5.34E-06 | -0.47 | 7.99 | 7.52 |
| 33213_g_at | RRBP1 | 5.39E-06 | 0.53 | 7.22 | 7.75 |
| 37720_at | HSPD1 | 5.40E-06 | -0.82 | 9.93 | 9.11 |
| 40381_at | ZNF510 | 5.40E-06 | -0.43 | 5.18 | 4.75 |
| 33241_at | MFAP3L | 5.44E-06 | 0.94 | 4.16 | 5.1 |
| 40574_at | MAP3K9 | 5.47E-06 | -0.45 | 5.36 | 4.91 |
| 33125_at | FANCL | 5.49E-06 | -0.75 | 5.03 | 4.28 |
| 40282_s_at | CFD | 5.52E-06 | 0.68 | 6.6 | 7.27 |
| 38317_at | TCEAL1 | 5.54E-06 | 0.52 | 6.59 | 7.11 |
| 40845_at | ILF3 | 5.55E-06 | -0.71 | 7.34 | 6.63 |
| 897_at | PKD1 | 5.55E-06 | -0.65 | 8.98 | 8.33 |
| 31584_at | TPT1 | 5.57E-06 | 0.51 | 9.86 | 10.37 |
| 40495_at | ORAI2 | 5.62E-06 | -0.59 | 6.71 | 6.12 |
| 35668_at | RAMP1 | 5.63E-06 | -1.11 | 6.99 | 5.88 |
| 40344_at | NLGN1 | 5.65E-06 | 0.39 | 3.23 | 3.62 |
| 39983_at | GLMN | 5.68E-06 | -0.28 | 3.74 | 3.46 |
| 41842_at | FLJ35348 | 5.69E-06 | -0.35 | 7.1 | 6.74 |
| 36604_at | UBE2N | 5.71E-06 | -0.71 | 6.68 | 5.97 |
| 34819_at | CD164 | 5.73E-06 | 1.06 | 5.79 | 6.85 |
| 41574_at | PNN | 5.74E-06 | -0.65 | 4.25 | 3.6 |
| 41211_at | RBM12 | 5.74E-06 | -0.43 | 5.18 | 4.75 |
| 39069_at | AEBP1 | 5.80E-06 | 0.87 | 7.44 | 8.31 |
| 39077_at | C17orf45 | 5.86E-06 | -1 | 6.79 | 5.79 |
| 40285_at | NVL | 5.92E-06 | -0.43 | 6.43 | 6.01 |
| 33075_at | PAK3 | 5.94E-06 | -0.68 | 4.18 | 3.5 |
| 1515_at | --- | 6.00E-06 | -1 | 7.2 | 6.2 |
| 38910_at | ATPAF2 | 6.06E-06 | -0.71 | 5.37 | 4.67 |
| 35756_at | GIPC1 | 6.08E-06 | 0.33 | 7.12 | 7.45 |
| 37955_at | TMEM4 | 6.11E-06 | 0.38 | 8.03 | 8.41 |
| 36617_at | ID1 | 6.16E-06 | 1.11 | 7.57 | 8.68 |
| 37092_at | PCDHA9 | 6.23E-06 | -0.69 | 5.57 | 4.87 |
| 36669_at | FOSB | 6.24E-06 | 1.29 | 8.32 | 9.61 |
| 32786_at | JUNB | 6.26E-06 | 1.56 | 7.82 | 9.38 |
| 32976_s_at | ZNF711 | 6.28E-06 | -0.5 | 3.8 | 3.3 |
| 39146_at | ATRX | 6.28E-06 | -0.37 | 4.02 | 3.65 |
| 37062_at | GABRA2 | 6.29E-06 | 0.38 | 3.98 | 4.36 |
| 40077_at | ACO1 | 6.30E-06 | 0.67 | 5.18 | 5.86 |
| 36630_at | TSC22D3 | 6.31E-06 | 0.31 | 7.17 | 7.47 |
| 38970_s_at | TNIP1 | 6.33E-06 | 0.37 | 7.15 | 7.51 |
| 479_at | DAB2 | 6.37E-06 | 1.3 | 6.26 | 7.56 |
| 35795_at | HDAC6 | 6.37E-06 | -0.32 | 7.33 | 7.01 |
| 37936_at | PRPF4 | 6.37E-06 | -0.45 | 5.88 | 5.42 |
| 40069_at | SVIL | 6.38E-06 | 0.83 | 5.74 | 6.57 |
| 40127_at | SFXN3 | 6.45E-06 | 0.57 | 7.4 | 7.97 |
| 384_at | PSMB10 | 6.49E-06 | 0.47 | 6.86 | 7.33 |
| 38760_f_at | BTN3A2 | 6.51E-06 | 0.64 | 5.66 | 6.3 |
| 40760_at | DHX57 | 6.58E-06 | -0.36 | 4.71 | 4.35 |
| 34427_g_at | MR1 | 6.58E-06 | 0.27 | 4.22 | 4.49 |
| 35620_at | TOMM40 | 6.60E-06 | -0.41 | 7.68 | 7.27 |
| 33819_at | LDHB | 6.63E-06 | -0.85 | 9.74 | 8.89 |
| 33229_at | RPS6KA3 | 6.66E-06 | 0.44 | 3.99 | 4.43 |
| 35642_at | MTX2 | 6.69E-06 | -0.58 | 5.93 | 5.35 |
| 32855_at | LDLR | 6.73E-06 | 0.98 | 6.43 | 7.41 |
| 527_at | CENPA | 6.74E-06 | -0.61 | 5.1 | 4.49 |
| 38281_at | CASP7 | 6.76E-06 | 0.37 | 3.5 | 3.87 |
| 38426_at | TAF11 | 6.79E-06 | -0.56 | 5.99 | 5.43 |
| 36672_at | PRCP | 7.05E-06 | 0.63 | 7.54 | 8.18 |
| 37573_at | ANGPTL2 | 7.05E-06 | 0.52 | 7.1 | 7.62 |
| 38019_at | CSNK1E | 7.07E-06 | -0.69 | 9.13 | 8.44 |
| 40583_at | PTPRJ | 7.07E-06 | 0.31 | 4.1 | 4.41 |
| 33978_at | ABCB4 | 7.16E-06 | 0.53 | 4.77 | 5.3 |
| 37695_at | RNF144A | 7.18E-06 | -0.84 | 6.42 | 5.58 |
| 189_s_at | PLAUR | 7.29E-06 | 0.55 | 6.95 | 7.5 |
| 35244_at | KIAA0460 | 7.35E-06 | -0.39 | 7.66 | 7.28 |
| 1589_s_at | IFNAR2 | 7.36E-06 | 0.43 | 4.14 | 4.57 |
| 1303_at | SH3BP2 | 7.38E-06 | 0.38 | 4.03 | 4.41 |
| 33714_at | HMGB3 | 7.42E-06 | -0.63 | 4.59 | 3.95 |
| 33735_at | SEZ6L | 7.43E-06 | -0.84 | 5.49 | 4.66 |
| 34011_at | HRK | 7.45E-06 | -0.65 | 6.94 | 6.29 |
| 39788_at | PKP4 | 7.61E-06 | 0.46 | 5.39 | 5.85 |
| 36273_at | CDK5R1 | 7.63E-06 | -0.51 | 5.84 | 5.33 |
| 37354_at | SP100 | 7.67E-06 | 0.35 | 3.44 | 3.79 |
| 36029_at | MPPED2 | 7.73E-06 | -1.17 | 5.21 | 4.04 |
| 41601_at | --- | 7.77E-06 | 0.62 | 4.23 | 4.84 |
| 1566_at | NCAM1 | 7.82E-06 | -0.72 | 6.88 | 6.15 |
| 567_s_at | LOC161527 | 7.83E-06 | 0.27 | 7.39 | 7.66 |
| 36950_at | TMED9 | 7.84E-06 | 0.35 | 8.64 | 8.99 |
| 41689_at | PLLP | 7.86E-06 | 0.47 | 5.87 | 6.35 |
| 519_g_at | NR1H2 | 7.88E-06 | 0.24 | 7.95 | 8.19 |
| 40577_at | C9orf97 | 7.89E-06 | -0.55 | 5.05 | 4.49 |
| 40693_at | KCNB1 | 7.90E-06 | -0.67 | 6.01 | 5.34 |
| 41356_at | BCL11A | 7.96E-06 | -0.8 | 6.05 | 5.25 |
| 41480_at | RAD51C | 7.98E-06 | -0.76 | 4.87 | 4.11 |
| 39686_g_at | ATXN10 | 8.02E-06 | -0.63 | 7.45 | 6.82 |
| 38933_at | KIFC1 | 8.02E-06 | -0.45 | 6.31 | 5.86 |
| 38414_at | CDC20 | 8.11E-06 | -0.52 | 7.36 | 6.84 |
| 39428_at | SH2B3 | 8.12E-06 | 0.66 | 6 | 6.67 |
| 41625_at | MED13 | 8.15E-06 | -0.71 | 7.32 | 6.6 |
| 40104_at | STK25 | 8.25E-06 | -0.35 | 8.51 | 8.16 |
| 33866_at | LOC643634 | 8.26E-06 | 0.9 | 6.35 | 7.25 |
| 658_at | THBS2 | 8.35E-06 | 1.61 | 4.23 | 5.84 |
| 35445_at | SNX26 | 8.36E-06 | -0.7 | 7.07 | 6.37 |
| 32063_at | PBX1 | 8.36E-06 | -0.45 | 6.58 | 6.13 |
| 1668_s_at | VHL | 8.38E-06 | -0.32 | 6.6 | 6.28 |
| 41412_at | CSDC2 | 8.45E-06 | -0.51 | 7.15 | 6.64 |
| 594_s_at | CSNK2A1 | 8.52E-06 | -0.35 | 7.52 | 7.18 |
| 33237_at | DDX46 | 8.61E-06 | -0.23 | 7.72 | 7.49 |
| 32478_f_at | SUMO4 | 8.83E-06 | -0.45 | 6.86 | 6.42 |
| 37161_at | RSL1D1 | 8.88E-06 | -0.88 | 5.25 | 4.37 |
| 34863_s_at | SCCPDH | 8.90E-06 | 0.5 | 5.29 | 5.79 |
| 40268_at | FOSL2 | 8.94E-06 | 0.46 | 8.51 | 8.97 |
| 34370_at | ARCN1 | 9.02E-06 | 0.53 | 5.64 | 6.17 |
| 37742_at | GLB1 | 9.05E-06 | 0.43 | 7.2 | 7.62 |
| 39838_at | CLASP1 | 9.10E-06 | -0.61 | 8.36 | 7.75 |
| 36250_at | ASPHD1 | 9.12E-06 | -0.34 | 7.58 | 7.25 |
| 32389_at | --- | 9.13E-06 | -1.79 | 7.8 | 6.01 |
| 41058_g_at | THEM2 | 9.21E-06 | 0.94 | 4.34 | 5.28 |
| 2028_s_at | E2F1 | 9.29E-06 | -0.35 | 6.49 | 6.14 |
| 418_at | MKI67 | 9.37E-06 | -0.55 | 3.65 | 3.11 |
| 1625_at | --- | 9.42E-06 | 0.34 | 2.67 | 3.01 |
| 31652_at | MYH15 | 9.46E-06 | -0.35 | 5.71 | 5.36 |
| 36261_at | SYT17 | 9.52E-06 | -0.49 | 6.81 | 6.31 |
| 39411_at | TIPARP | 9.52E-06 | 0.48 | 5.31 | 5.79 |
| 40805_at | SIPA1L1 | 9.56E-06 | 0.7 | 4.49 | 5.19 |
| 38176_at | GNB5 | 9.60E-06 | -0.52 | 5.15 | 4.63 |
| 36538_at | PPP1R13B | 9.61E-06 | -0.6 | 6.35 | 5.74 |
| 33556_at | HYPE | 9.64E-06 | 0.29 | 3.23 | 3.52 |
| 38690_at | CLDND1 | 9.66E-06 | 0.39 | 7.42 | 7.82 |
| 1710_s_at | TOP1 | 9.67E-06 | -0.51 | 5.58 | 5.07 |
| 35775_at | SMYD2 | 9.78E-06 | -0.47 | 3.23 | 2.76 |
| 40211_at | HNRNPA1 | 9.79E-06 | -0.76 | 10.06 | 9.3 |
| 40589_at | SNTB2 | 9.79E-06 | 0.53 | 5.27 | 5.8 |
| 36884_at | CD163 | 1.00E-05 | 0.44 | 4.21 | 4.66 |
| 38248_at | ProSAPiP1 | 1.01E-05 | -0.88 | 7.62 | 6.75 |
| 35848_at | ZMIZ1 | 1.01E-05 | -0.95 | 7.76 | 6.82 |
| 40549_at | CDK5 | 1.02E-05 | -0.61 | 7.06 | 6.45 |
| 39687_at | ATXN10 | 1.02E-05 | -0.54 | 6.6 | 6.06 |
| 35710_s_at | STRA13 | 1.03E-05 | -0.49 | 7.8 | 7.31 |
| 36879_at | ECGF1 | 1.04E-05 | 0.72 | 7.1 | 7.82 |
| 40366_at | SELP | 1.05E-05 | 0.7 | 5.32 | 6.02 |
| 31808_at | ING3 | 1.06E-05 | -0.4 | 4.19 | 3.79 |
| 36176_at | TBCC | 1.06E-05 | -0.61 | 5.1 | 4.49 |
| 37686_s_at | UNG | 1.07E-05 | -0.83 | 6.44 | 5.61 |
| 38076_at | ATP5G1 | 1.08E-05 | -0.43 | 8.74 | 8.31 |
| 38759_at | BTN3A2 | 1.08E-05 | 0.53 | 5.92 | 6.45 |
| 34347_at | C3orf60 | 1.09E-05 | 0.47 | 8.02 | 8.49 |
| 41325_at | KCNK3 | 1.10E-05 | -0.61 | 7.46 | 6.85 |
| 34055_at | ACVR1B | 1.11E-05 | -0.33 | 4.23 | 3.9 |
| 225_at | GNB3 | 1.11E-05 | -0.29 | 7.15 | 6.86 |
| 36679_at | SRPR | 1.11E-05 | 0.41 | 5.73 | 6.15 |
| 34755_at | PARP2 | 1.12E-05 | -0.49 | 6.94 | 6.45 |
| 38063_at | PBXIP1 | 1.12E-05 | 0.32 | 7.38 | 7.71 |
| 34325_at | PQBP1 | 1.13E-05 | -0.52 | 7.37 | 6.85 |
| 32921_at | LOC730390 | 1.13E-05 | -1.07 | 5.14 | 4.07 |
| 41453_at | DLG3 | 1.13E-05 | -0.43 | 6.4 | 5.97 |
| 41049_at | IRS1 | 1.13E-05 | 0.61 | 4.24 | 4.85 |
| 122_at | PRIM2 | 1.13E-05 | -0.34 | 5.37 | 5.03 |
| 40986_s_at | 76P | 1.14E-05 | -0.64 | 8 | 7.37 |
| 37171_at | KIF23 | 1.15E-05 | -0.4 | 3.07 | 2.67 |
| 37246_at | ACD | 1.16E-05 | -0.24 | 8.81 | 8.57 |
| 41528_at | LOC130074 | 1.16E-05 | -0.35 | 7.19 | 6.84 |
| 38729_at | FKBP4 | 1.16E-05 | -0.55 | 9.03 | 8.49 |
| 178_f_at | LOC732139 | 1.17E-05 | -0.38 | 6.39 | 6.01 |
| 40924_at | TMEM118 | 1.18E-05 | -0.81 | 5.82 | 5.01 |
| 33960_s_at | CACNA1B | 1.18E-05 | -0.25 | 8.07 | 7.82 |
| 39252_at | SUCLG2 | 1.18E-05 | 0.29 | 2.98 | 3.27 |
| 41079_at | ACCN3 | 1.19E-05 | -0.52 | 7.52 | 7 |
| 38796_at | C1QB | 1.21E-05 | 1.27 | 7.52 | 8.79 |
| 424_s_at | FGFR1 | 1.22E-05 | 0.4 | 7.91 | 8.32 |
| 34007_at | GRM8 | 1.23E-05 | -0.79 | 3.58 | 2.79 |
| 37501_at | NKRF | 1.24E-05 | -0.45 | 5.25 | 4.8 |
| 41223_at | COX5A | 1.25E-05 | -0.67 | 7.15 | 6.47 |
| 33892_at | PKP2 | 1.25E-05 | 0.27 | 2.57 | 2.85 |
| 32045_at | DIDO1 | 1.26E-05 | -0.46 | 6.29 | 5.83 |
| 34853_at | FLRT2 | 1.27E-05 | 0.98 | 5.25 | 6.23 |
| 35618_at | HELZ | 1.28E-05 | -0.64 | 6.59 | 5.95 |
| 32846_s_at | KTN1 | 1.28E-05 | 0.55 | 6.73 | 7.28 |
| 33399_at | C14orf147 | 1.28E-05 | 0.67 | 6.85 | 7.52 |
| 1972_s_at | MAP2 | 1.28E-05 | -0.72 | 6.68 | 5.96 |
| 37417_at | POU2F2 | 1.28E-05 | -0.43 | 8.37 | 7.94 |
| 32583_at | JUN | 1.28E-05 | 0.95 | 6.7 | 7.65 |
| 34267_r_at | LEPR | 1.29E-05 | 0.31 | 2.67 | 2.98 |
| 33835_at | TSPYL4 | 1.29E-05 | -1.01 | 7.78 | 6.76 |
| 41045_at | SECTM1 | 1.29E-05 | 0.31 | 6.59 | 6.9 |
| 41037_at | TEAD4 | 1.30E-05 | 0.44 | 5.14 | 5.57 |
| 32887_at | AZI1 | 1.30E-05 | -0.49 | 7.32 | 6.83 |
| 34292_at | TMEM187 | 1.31E-05 | 0.27 | 6.2 | 6.47 |
| 35504_at | ARID1A | 1.31E-05 | -0.46 | 6.05 | 5.58 |
| 39153_r_at | COIL | 1.31E-05 | -0.32 | 2.76 | 2.44 |
| 35256_at | SLC5A6 | 1.32E-05 | -0.42 | 7.57 | 7.14 |
| 35407_at | CDC25A | 1.32E-05 | -0.38 | 5.58 | 5.21 |
| 33810_at | TRRAP | 1.33E-05 | -0.72 | 6.4 | 5.67 |
| 32058_at | CHST10 | 1.34E-05 | -0.26 | 6.59 | 6.33 |
| 1201_at | RAB33A | 1.35E-05 | -0.48 | 5.27 | 4.79 |
| 34284_at | IFT88 | 1.35E-05 | 0.33 | 4.55 | 4.87 |
| 39570_at | CAMSAP1 | 1.36E-05 | -0.4 | 7.81 | 7.41 |
| 38031_at | EIF4A3 | 1.36E-05 | -0.64 | 7.55 | 6.91 |
| 35224_at | MGC14376 | 1.36E-05 | 0.39 | 5.47 | 5.86 |
| 38625_g_at | SLC12A4 | 1.37E-05 | 0.3 | 6.67 | 6.97 |
| 41712_at | AQR | 1.37E-05 | -0.48 | 5.29 | 4.81 |
| 40303_at | TFAP2C | 1.38E-05 | 0.32 | 4.06 | 4.38 |
| 36634_at | BTG2 | 1.38E-05 | 1.18 | 7.26 | 8.44 |
| 36214_at | KLF4 | 1.39E-05 | 0.4 | 5.61 | 6.01 |
| 40976_at | KATNB1 | 1.39E-05 | -0.35 | 8.33 | 7.98 |
| 36026_at | PGAM2 | 1.39E-05 | -0.51 | 5.27 | 4.76 |
| 37657_at | PALM | 1.39E-05 | -0.5 | 8.49 | 7.99 |
| 735_s_at | --- | 1.40E-05 | 0.5 | 7.32 | 7.82 |
| 39430_at | TNKS | 1.40E-05 | -0.66 | 7.42 | 6.75 |
| 33514_at | CAMK4 | 1.42E-05 | -0.36 | 5.1 | 4.75 |
| 39382_at | TRIM2 | 1.44E-05 | -1.29 | 8.14 | 6.84 |
| 1943_at | CCNA2 | 1.45E-05 | -0.82 | 6.09 | 5.26 |
| 31598_s_at | GALE | 1.45E-05 | -0.32 | 6.15 | 5.83 |
| 38747_at | CD34 | 1.45E-05 | 0.54 | 7.25 | 7.79 |
| 33678_i_at | TUBB2C | 1.46E-05 | -1.03 | 12.27 | 11.24 |
| 41452_at | ZKSCAN5 | 1.47E-05 | -0.27 | 6.23 | 5.96 |
| 36247_f_at | ADH1C | 1.49E-05 | 0.74 | 5.95 | 6.69 |
| 1562_g_at | DUSP8 | 1.49E-05 | -0.86 | 7.78 | 6.91 |
| 33726_at | POLR2D | 1.49E-05 | -0.41 | 4.84 | 4.43 |
| 31463_s_at | hCG_2023776 | 1.49E-05 | -0.71 | 10.45 | 9.74 |
| 38300_at | FZD1 | 1.50E-05 | 0.28 | 2.77 | 3.05 |
| 40351_at | GNB3 | 1.51E-05 | -0.27 | 6.72 | 6.46 |
| 34059_at | PVT1 | 1.51E-05 | -0.51 | 4.3 | 3.78 |
| 35812_at | TNPO3 | 1.54E-05 | -0.41 | 6.87 | 6.47 |
| 35792_at | MGLL | 1.54E-05 | 0.63 | 5.55 | 6.18 |
| 41146_at | PARP1 | 1.55E-05 | -0.61 | 7.89 | 7.28 |
| 33403_at | C1orf77 | 1.55E-05 | -0.73 | 6.51 | 5.79 |
| 37014_at | MX1 | 1.55E-05 | 1.02 | 5.6 | 6.62 |
| 2049_s_at | JUNB | 1.56E-05 | 1.26 | 7.24 | 8.5 |
| 35743_at | CPSF4 | 1.56E-05 | -0.54 | 7.46 | 6.92 |
| 38065_at | HMGB2 | 1.57E-05 | -1.24 | 8.26 | 7.02 |
| 41081_at | BUB1 | 1.57E-05 | -0.41 | 3.19 | 2.78 |
| 1452_at | LMO4 | 1.58E-05 | 0.88 | 6.53 | 7.41 |
| 330_s_at | --- | 1.58E-05 | 0.71 | 6.57 | 7.28 |
| 40362_at | NFKB2 | 1.58E-05 | 0.39 | 6.08 | 6.46 |
| 41722_at | NNT | 1.59E-05 | -0.54 | 3.88 | 3.35 |
| 41610_at | LAMA5 | 1.60E-05 | 0.55 | 6.65 | 7.2 |
| 41699_f_at | BRD1 | 1.60E-05 | -0.38 | 7.85 | 7.47 |
| 36594_s_at | EXT2 | 1.60E-05 | 0.27 | 5.49 | 5.77 |
| 41671_at | EML1 | 1.61E-05 | -0.3 | 5.88 | 5.58 |
| 39601_at | RASSF1 | 1.61E-05 | 0.22 | 7.71 | 7.93 |
| 34599_at | TUB | 1.61E-05 | -0.34 | 6.9 | 6.56 |
| 41642_at | SMA4 | 1.61E-05 | -1 | 5.37 | 4.37 |
| 33984_at | HSP90AB1 | 1.61E-05 | -0.56 | 11.06 | 10.51 |
| 41441_at | TBP | 1.61E-05 | -0.33 | 6.01 | 5.68 |
| 33299_at | GPR125 | 1.61E-05 | -0.76 | 5.27 | 4.5 |
| 40912_s_at | BPHL | 1.61E-05 | -0.43 | 7.89 | 7.46 |
| 35400_at | --- | 1.62E-05 | -0.88 | 4.55 | 3.67 |
| 36254_at | TAC1 | 1.64E-05 | 0.71 | 3.17 | 3.88 |
| 33495_at | PPFIA2 | 1.65E-05 | -0.51 | 5.57 | 5.06 |
| 34768_at | TXNDC1 | 1.65E-05 | 0.41 | 4.37 | 4.78 |
| 1850_at | MLH1 | 1.65E-05 | -0.29 | 7.56 | 7.27 |
| 1287_at | PARP1 | 1.67E-05 | -0.63 | 8.3 | 7.67 |
| 36186_at | RNPS1 | 1.67E-05 | -0.55 | 9.61 | 9.05 |
| 770_at | GPX3 | 1.67E-05 | 1.19 | 7.28 | 8.47 |
| 37950_at | PREP | 1.68E-05 | -0.47 | 5.99 | 5.52 |
| 1747_at | DNASE2 | 1.69E-05 | 0.36 | 6.47 | 6.83 |
| 39384_at | ELAVL1 | 1.69E-05 | -0.48 | 3.29 | 2.81 |
| 265_s_at | SELE | 1.69E-05 | 1.13 | 3.11 | 4.24 |
| 41742_s_at | OPTN | 1.70E-05 | 0.75 | 5.83 | 6.58 |
| 39791_at | ATP2A2 | 1.70E-05 | -0.77 | 7.2 | 6.43 |
| 38834_at | TOPBP1 | 1.70E-05 | -0.73 | 4.85 | 4.12 |
| 33777_at | TBXAS1 | 1.70E-05 | 0.54 | 5.81 | 6.35 |
| 468_at | FGF13 | 1.71E-05 | -0.86 | 6.02 | 5.16 |
| 644_at | RND2 | 1.71E-05 | -0.67 | 6.57 | 5.9 |
| 35026_f_at | ZNF273 | 1.72E-05 | -0.58 | 3.96 | 3.38 |
| 34699_at | CD2AP | 1.73E-05 | 0.63 | 4.73 | 5.36 |
| 33910_at | PTPRD | 1.74E-05 | -1.41 | 4.94 | 3.52 |
| 39955_at | DLEU2 | 1.74E-05 | -0.36 | 3.33 | 2.97 |
| 32196_at | CAND1 | 1.75E-05 | -0.5 | 4.5 | 4 |
| 33035_at | TIPRL | 1.75E-05 | -0.52 | 5.79 | 5.27 |
| 39433_at | C22orf9 | 1.75E-05 | -0.43 | 5.26 | 4.83 |
| 32343_at | EPB41 | 1.76E-05 | -0.49 | 4.39 | 3.9 |
| 36184_at | PLOD1 | 1.76E-05 | 0.4 | 8.22 | 8.62 |
| 39185_at | WDR45L | 1.78E-05 | -0.44 | 7.25 | 6.81 |
| 41054_at | FCHO1 | 1.78E-05 | -0.31 | 6.47 | 6.16 |
| 1983_at | CCND2 | 1.78E-05 | 0.23 | 6.44 | 6.67 |
| 1171_s_at | --- | 1.80E-05 | -0.34 | 6.79 | 6.45 |
| 33326_at | RAB21 | 1.81E-05 | 0.59 | 5.43 | 6.01 |
| 39342_at | MARS | 1.82E-05 | -0.45 | 6.87 | 6.42 |
| 33073_at | PAK3 | 1.82E-05 | -0.74 | 5.78 | 5.04 |
| 34345_at | PRPF6 | 1.82E-05 | -0.45 | 8.25 | 7.8 |
| 1256_at | PTPRD | 1.83E-05 | -0.45 | 5.48 | 5.04 |
| 37226_at | BNIP1 | 1.83E-05 | -0.22 | 5.49 | 5.27 |
| 32088_at | BLZF1 | 1.85E-05 | 0.18 | 2.58 | 2.76 |
| 41355_at | BCL11A | 1.87E-05 | -0.77 | 5.51 | 4.74 |
| 36253_at | BGLAP | 1.88E-05 | -0.45 | 6.65 | 6.2 |
| 37912_at | TRAF4 | 1.91E-05 | -0.3 | 7.93 | 7.62 |
| 34637_f_at | ADH1A | 1.92E-05 | 1.2 | 7.06 | 8.26 |
| 39830_at | RPL27 | 1.92E-05 | -0.59 | 10.63 | 10.04 |
| 36912_at | CHAF1B | 1.93E-05 | -0.26 | 5.98 | 5.72 |
| 39401_at | LOC552889 | 1.95E-05 | -0.49 | 8.72 | 8.23 |
| 32406_at | C20orf117 | 1.95E-05 | -0.34 | 5.2 | 4.86 |
| 39862_at | POL3S | 1.95E-05 | -0.27 | 7.56 | 7.29 |
| 36181_at | LASP1 | 1.96E-05 | 0.66 | 8.28 | 8.94 |
| 33363_at | EIF2AK1 | 1.96E-05 | -0.28 | 6 | 5.72 |
| 31783_at | RENBP | 1.96E-05 | 0.43 | 5.84 | 6.27 |
| 36618_g_at | ID1 | 1.97E-05 | 0.81 | 6.51 | 7.32 |
| 39738_at | MYH9 | 1.99E-05 | 0.97 | 8.02 | 8.99 |
| 32726_g_at | BID | 1.99E-05 | -0.26 | 3.53 | 3.27 |
| 40154_at | TTLL3 | 2.00E-05 | 0.25 | 7.8 | 8.05 |
| 36010_at | MEOX1 | 2.00E-05 | 0.4 | 3.64 | 4.04 |
| 32073_at | JMJD2A | 2.01E-05 | -0.28 | 7.37 | 7.1 |
| 31733_at | P2RX3 | 2.02E-05 | -0.38 | 6.52 | 6.14 |
| 36074_at | IPW | 2.03E-05 | -0.72 | 5.23 | 4.52 |
| 1007_s_at | DDR1 | 2.03E-05 | 0.6 | 7.42 | 8.02 |
| 33228_g_at | IL10RB | 2.05E-05 | 0.56 | 6.99 | 7.55 |
| 32102_at | SACS | 2.05E-05 | -0.77 | 5.73 | 4.97 |
| 38708_at | RAN | 2.06E-05 | -0.66 | 10.37 | 9.71 |
| 1202_g_at | RAB33A | 2.06E-05 | -0.5 | 6.3 | 5.79 |
| 37333_at | DNMT1 | 2.06E-05 | -0.47 | 7.27 | 6.79 |
| 41426_at | ZNF212 | 2.06E-05 | -0.27 | 7.23 | 6.96 |
| 33253_at | TRIM14 | 2.06E-05 | 0.49 | 5.44 | 5.93 |
| 37585_at | SNRPA1 | 2.07E-05 | -0.33 | 6.92 | 6.59 |
| 156_s_at | GPR19 | 2.07E-05 | -0.46 | 4.25 | 3.8 |
| 35859_f_at | PMS2L3 | 2.08E-05 | -0.48 | 5.5 | 5.02 |
| 35845_at | SEC24B | 2.08E-05 | 0.53 | 5.71 | 6.24 |
| 2025_s_at | APEX1 | 2.08E-05 | -0.69 | 8.24 | 7.54 |
| 40118_at | ZNF3 | 2.10E-05 | -0.46 | 7.57 | 7.11 |
| 1824_s_at | PCNA | 2.10E-05 | -0.7 | 7.23 | 6.52 |
| 38528_at | ACACA | 2.11E-05 | -0.4 | 5.96 | 5.56 |
| 34472_at | FZD6 | 2.11E-05 | 0.5 | 3.57 | 4.07 |
| 36259_at | C7orf54 | 2.12E-05 | -0.24 | 4.37 | 4.13 |
| 41344_s_at | PURA | 2.13E-05 | 0.73 | 7.53 | 8.25 |
| 39074_at | FLAD1 | 2.15E-05 | -0.3 | 6.39 | 6.1 |
| 36300_at | CALCA | 2.16E-05 | 0.75 | 6.14 | 6.89 |
| 36701_at | FUT9 | 2.18E-05 | -1.16 | 4.01 | 2.85 |
| 950_at | TLOC1 | 2.18E-05 | 0.38 | 4.47 | 4.86 |
| 37725_at | PPP1CC | 2.19E-05 | -0.48 | 7.76 | 7.28 |
| 36267_at | NFKBIL1 | 2.19E-05 | -0.23 | 6.43 | 6.2 |
| 366_s_at | NEK2 | 2.20E-05 | -0.42 | 3.84 | 3.42 |
| 41247_at | --- | 2.21E-05 | -0.5 | 6.34 | 5.84 |
| 31751_f_at | HIST1H4L | 2.22E-05 | -0.42 | 4.88 | 4.46 |
| 391_at | PPP5C | 2.23E-05 | -0.38 | 7.11 | 6.73 |
| 35149_at | CD40 | 2.25E-05 | 0.23 | 5.49 | 5.72 |
| 34126_at | BRSK2 | 2.25E-05 | -0.6 | 6.61 | 6.01 |
| 37571_at | DOCK9 | 2.25E-05 | 0.72 | 3.54 | 4.26 |
| 546_at | PKIA | 2.26E-05 | -1.04 | 5.09 | 4.05 |
| 39666_at | GNG4 | 2.27E-05 | -0.87 | 7.72 | 6.86 |
| 34197_at | PIK3R2 | 2.27E-05 | -0.31 | 6.86 | 6.55 |
| 448_s_at | MEN1 | 2.28E-05 | -0.4 | 6.96 | 6.56 |
| 36095_at | CLIP3 | 2.28E-05 | -0.7 | 8.79 | 8.09 |
| 884_at | ITGA3 | 2.29E-05 | 0.36 | 7.59 | 7.95 |
| 32300_s_at | TH | 2.31E-05 | -1.78 | 9.21 | 7.43 |
| 31833_at | PIP5K1A | 2.33E-05 | -0.26 | 7.72 | 7.46 |
| 34558_at | OPRL1 | 2.34E-05 | -0.27 | 7.48 | 7.21 |
| 41628_at | FUT8 | 2.34E-05 | 0.36 | 3.16 | 3.52 |
| 32064_at | MED1 | 2.35E-05 | -0.49 | 6.83 | 6.34 |
| 38340_at | HIP1R | 2.36E-05 | -0.49 | 7.65 | 7.16 |
| 40068_at | STX5 | 2.36E-05 | 0.3 | 6.07 | 6.37 |
| 37823_at | CCL8 | 2.37E-05 | 0.6 | 3.63 | 4.23 |
| 36202_at | PKIA | 2.37E-05 | -1.07 | 6.8 | 5.73 |
| 41256_at | EEF1D | 2.38E-05 | 0.61 | 9.02 | 9.63 |
| 36852_at | TUSC3 | 2.40E-05 | -1.01 | 6.63 | 5.62 |
| 35839_at | SQLE | 2.43E-05 | -0.63 | 5.2 | 4.58 |
| 33235_at | NAV3 | 2.43E-05 | 0.94 | 4.55 | 5.49 |
| 38748_at | ADARB1 | 2.44E-05 | 0.78 | 4.59 | 5.36 |
| 33783_at | PLXNB1 | 2.45E-05 | -0.31 | 8.13 | 7.81 |
| 38804_at | CSE1L | 2.46E-05 | -0.63 | 6.32 | 5.69 |
| 35917_at | MAP1A | 2.48E-05 | 0.66 | 8.99 | 9.66 |
| 32931_at | --- | 2.49E-05 | -0.39 | 5.21 | 4.83 |
| 39684_at | MPP3 | 2.49E-05 | -0.45 | 7.24 | 6.79 |
| 39532_at | RIN1 | 2.49E-05 | 0.44 | 4.57 | 5.01 |
| 34510_at | CDT1 | 2.50E-05 | -0.43 | 7.12 | 6.68 |
| 36468_at | DTNA | 2.50E-05 | -0.26 | 5.59 | 5.32 |
| 36826_at | GTF2F1 | 2.50E-05 | -0.26 | 7.41 | 7.15 |
| 34254_at | RALGDS | 2.50E-05 | -0.42 | 6.44 | 6.02 |
| 33836_at | DKFZp547E087 | 2.52E-05 | -0.7 | 8.8 | 8.1 |
| 33157_at | INSM1 | 2.54E-05 | -1.33 | 5.79 | 4.46 |
| 36846_s_at | LSM7 | 2.54E-05 | -0.56 | 8.29 | 7.73 |
| 40215_at | UGCG | 2.55E-05 | 0.88 | 7.49 | 8.38 |
| 35223_at | HPS5 | 2.55E-05 | 0.42 | 3 | 3.43 |
| 41795_at | NCK1 | 2.58E-05 | 0.29 | 5.16 | 5.44 |
| 38408_at | TSPAN7 | 2.58E-05 | -0.75 | 8.01 | 7.26 |
| 393_s_at | RUNX1 | 2.58E-05 | 0.78 | 4.03 | 4.81 |
| 37760_at | BAIAP2 | 2.59E-05 | 0.62 | 7.05 | 7.66 |
| 41664_at | TIMM44 | 2.59E-05 | -0.26 | 7.54 | 7.29 |
| 39856_at | RPL36AL | 2.59E-05 | 0.74 | 8.49 | 9.23 |
| 37595_at | --- | 2.60E-05 | -1.07 | 6.56 | 5.49 |
| 35713_at | FANCC | 2.60E-05 | -0.32 | 5.39 | 5.08 |
| 39033_at | TMEM59 | 2.62E-05 | 0.68 | 7.86 | 8.54 |
| 38618_at | LIMK2 | 2.65E-05 | -0.61 | 6.75 | 6.13 |
| 41554_at | OSGIN2 | 2.65E-05 | 0.28 | 2.96 | 3.24 |
| 36030_at | HOM-TES-103 | 2.69E-05 | 0.34 | 7.72 | 8.07 |
| 40563_at | --- | 2.70E-05 | -0.35 | 4.39 | 4.04 |
| 34895_at | SEC23IP | 2.70E-05 | -0.43 | 4.28 | 3.85 |
| 39171_at | CTNNBIP1 | 2.70E-05 | -0.35 | 6.86 | 6.51 |
| 35189_at | TMEM24 | 2.71E-05 | -0.26 | 4.33 | 4.07 |
| 37071_at | CNGB1 | 2.72E-05 | -1.41 | 6.71 | 5.3 |
| 39929_at | KIAA0922 | 2.72E-05 | -0.49 | 5.68 | 5.19 |
| 38472_at | KIAA0143 | 2.74E-05 | 0.5 | 5.81 | 6.3 |
| 39155_at | PSMD3 | 2.75E-05 | -0.64 | 8.19 | 7.54 |
| 37519_at | ASGR1 | 2.78E-05 | -0.39 | 6.77 | 6.38 |
| 33145_at | FANCA | 2.78E-05 | -0.4 | 6.15 | 5.75 |
| 37474_at | OIP5 | 2.78E-05 | -0.42 | 3.55 | 3.13 |
| 36448_at | ABI2 | 2.78E-05 | -0.91 | 6.17 | 5.27 |
| 36153_at | DHX9 | 2.78E-05 | -0.54 | 7.3 | 6.76 |
| 39065_s_at | TTC3 | 2.82E-05 | -0.85 | 7.87 | 7.02 |
| 33794_g_at | TCF20 | 2.83E-05 | -0.32 | 6.55 | 6.23 |
| 31707_at | ZNF10 | 2.86E-05 | -0.3 | 3.99 | 3.69 |
| 37155_at | CNTNAP2 | 2.87E-05 | -1.31 | 7.91 | 6.6 |
| 36180_s_at | MAPKAPK2 | 2.89E-05 | 0.43 | 6.46 | 6.89 |
| 35450_s_at | GTF2I | 2.93E-05 | -0.86 | 10.05 | 9.19 |
| 38358_at | DDX52 | 2.96E-05 | -0.43 | 4.59 | 4.16 |
| 39614_at | KIAA0802 | 2.96E-05 | -0.74 | 5.26 | 4.52 |
| 35084_at | AMH | 2.97E-05 | -1.04 | 6.53 | 5.49 |
| 1237_at | IER3 | 2.98E-05 | 0.98 | 6.05 | 7.02 |
| 35158_at | MYCN | 2.99E-05 | -2.37 | 7.3 | 4.93 |
| 38573_at | YAF2 | 3.00E-05 | -0.32 | 4.95 | 4.63 |
| 39426_at | TCERG1 | 3.00E-05 | -0.59 | 5.14 | 4.55 |
| 35355_at | DHX30 | 3.04E-05 | -0.34 | 8.77 | 8.43 |
| 387_at | CDK9 | 3.04E-05 | 0.35 | 6.54 | 6.89 |
| 33404_at | CAP2 | 3.05E-05 | -0.66 | 4.96 | 4.29 |
| 357_at | DNM1L | 3.06E-05 | -0.61 | 5.11 | 4.5 |
| 32530_at | YWHAQ | 3.06E-05 | -0.81 | 9.35 | 8.54 |
| 41046_s_at | ZMYM3 | 3.08E-05 | -0.3 | 6.73 | 6.43 |
| 33690_at | --- | 3.08E-05 | 0.43 | 5.81 | 6.24 |
| 38916_at | CXorf6 | 3.09E-05 | 0.46 | 5.45 | 5.9 |
| 1818_at | --- | 3.10E-05 | 0.6 | 6.61 | 7.21 |
| 35963_at | PFDN6 | 3.11E-05 | -0.39 | 6.4 | 6.01 |
| 37685_at | PICALM | 3.20E-05 | 0.71 | 6.47 | 7.18 |
| 1678_g_at | IGFBP5 | 3.21E-05 | 0.87 | 4.51 | 5.38 |
| 35321_at | TLK2 | 3.25E-05 | -0.58 | 8.05 | 7.46 |
| 34780_at | PLXNB2 | 3.26E-05 | 0.35 | 8.66 | 9 |
| 37293_at | CKAP5 | 3.27E-05 | -0.56 | 5.83 | 5.26 |
| 39272_g_at | RAB4B | 3.27E-05 | 0.29 | 6.25 | 6.55 |
| 41240_at | PSMA2 | 3.28E-05 | -0.38 | 3.58 | 3.2 |
| 34379_at | ERAL1 | 3.29E-05 | -0.31 | 8.24 | 7.92 |
| 35753_at | PRPF8 | 3.29E-05 | -0.52 | 8.86 | 8.34 |
| 35435_s_at | HADH | 3.30E-05 | 0.38 | 5.66 | 6.04 |
| 34706_at | MGA | 3.32E-05 | -0.26 | 6.32 | 6.06 |
| 36111_s_at | SFRS2 | 3.33E-05 | -0.6 | 9.7 | 9.1 |
| 39583_at | LRRN2 | 3.34E-05 | -0.42 | 6.64 | 6.22 |
| 32429_f_at | ZNF33B | 3.34E-05 | -0.56 | 4.04 | 3.48 |
| 38440_s_at | ARMCX6 | 3.35E-05 | 0.4 | 6.93 | 7.33 |
| 40896_at | POU2F1 | 3.36E-05 | -0.39 | 6.66 | 6.28 |
| 33913_at | BAT2 | 3.36E-05 | -0.34 | 8.85 | 8.51 |
| 35706_at | NR1D2 | 3.38E-05 | 0.38 | 3.3 | 3.68 |
| 37984_s_at | ARF6 | 3.38E-05 | 0.79 | 6.06 | 6.85 |
| 36889_at | FCER1G | 3.38E-05 | 1.06 | 5.85 | 6.9 |
| 37953_s_at | ACCN2 | 3.40E-05 | -0.49 | 5.59 | 5.1 |
| 33896_at | EP300 | 3.41E-05 | -0.33 | 6.3 | 5.97 |
| 31736_at | --- | 3.42E-05 | -0.47 | 6.12 | 5.66 |
| 36492_at | PSMD9 | 3.43E-05 | 0.3 | 7.34 | 7.65 |
| 35813_at | TNPO3 | 3.44E-05 | -0.35 | 8.16 | 7.8 |
| 394_at | BLMH | 3.45E-05 | -0.52 | 5.95 | 5.44 |
| 33474_at | GLI1 | 3.47E-05 | 0.38 | 6.36 | 6.74 |
| 40783_s_at | PI4KA | 3.48E-05 | -0.59 | 9.79 | 9.2 |
| 40056_at | SLC35B1 | 3.49E-05 | -0.32 | 6.43 | 6.11 |
| 39832_at | ARS2 | 3.51E-05 | -0.32 | 8.23 | 7.91 |
| 36736_f_at | PSPH | 3.52E-05 | -0.56 | 3.46 | 2.9 |
| 39767_at | CCT8 | 3.53E-05 | -0.71 | 8.41 | 7.7 |
| 714_at | --- | 3.54E-05 | -0.25 | 4.89 | 4.64 |
| 40538_at | SLC26A10 | 3.55E-05 | -0.53 | 6.6 | 6.07 |
| 36981_at | SRP9 | 3.56E-05 | -0.63 | 8.28 | 7.64 |
| 35100_at | ST8SIA3 | 3.56E-05 | -0.44 | 4.39 | 3.95 |
| 40153_at | TAP1 | 3.57E-05 | 0.44 | 6.32 | 6.76 |
| 36143_at | CASP3 | 3.60E-05 | -0.62 | 5.4 | 4.78 |
| 36243_at | TLR1 | 3.61E-05 | 0.32 | 4.13 | 4.45 |
| 35162_s_at | ACVR2A | 3.62E-05 | -0.44 | 5.88 | 5.44 |
| 33017_at | GPLD1 | 3.62E-05 | -0.33 | 3.23 | 2.9 |
| 39022_at | INTS1 | 3.63E-05 | -0.39 | 7.8 | 7.41 |
| 37849_at | SLIT1 | 3.63E-05 | -0.74 | 7.33 | 6.59 |
| 40647_at | XK | 3.68E-05 | -0.68 | 5.88 | 5.2 |
| 34902_at | KIAA0492 | 3.70E-05 | -0.36 | 6.6 | 6.24 |
| 39142_at | NUDT21 | 3.70E-05 | -0.89 | 4.85 | 3.96 |
| 41822_at | ZNF324 | 3.73E-05 | -0.28 | 6.32 | 6.05 |
| 39975_at | UBFD1 | 3.74E-05 | -0.36 | 3.85 | 3.49 |
| 36814_at | KIAA1109 | 3.75E-05 | 0.62 | 6.26 | 6.88 |
| 36960_at | PHC2 | 3.80E-05 | 0.53 | 6.55 | 7.08 |
| 33198_at | ARL2BP | 3.80E-05 | 0.52 | 7.94 | 8.46 |
| 37636_at | PHF16 | 3.84E-05 | -0.34 | 4.52 | 4.18 |
| 36036_at | SPTB | 3.84E-05 | -0.41 | 6.19 | 5.78 |
| 38543_at | ALK | 3.85E-05 | -0.99 | 6.75 | 5.76 |
| 1786_at | MERTK | 3.86E-05 | 0.51 | 5.07 | 5.58 |
| 32918_at | --- | 3.88E-05 | -0.24 | 6.68 | 6.44 |
| 32559_s_at | LSM4 | 3.95E-05 | -0.51 | 6.83 | 6.31 |
| 33901_at | SLC29A1 | 3.95E-05 | -0.71 | 8.33 | 7.62 |
| 33062_at | GSTA1 | 3.97E-05 | -0.28 | 6.33 | 6.05 |
| 37910_at | HCFC1 | 3.97E-05 | -0.58 | 6.22 | 5.64 |
| 39386_at | MAD2L1BP | 3.98E-05 | -0.33 | 6.21 | 5.88 |
| 36303_f_at | ZNF85 | 4.01E-05 | -0.35 | 5.6 | 5.24 |
| 1676_s_at | EEF1G | 4.06E-05 | -0.57 | 11.29 | 10.72 |
| 39067_at | MEA1 | 4.09E-05 | -0.32 | 7.83 | 7.5 |
| 38337_at | ZNF193 | 4.09E-05 | -0.47 | 6.52 | 6.05 |
| 41639_at | NCAPH | 4.13E-05 | -0.35 | 4.89 | 4.55 |
| 40905_s_at | PRPF31 | 4.15E-05 | -0.3 | 7.1 | 6.8 |
| 40551_at | SPTBN1 | 4.18E-05 | 0.29 | 3.39 | 3.68 |
| 37644_s_at | FAS | 4.20E-05 | 0.29 | 2.82 | 3.11 |
| 146_at | PI4KB | 4.20E-05 | 0.32 | 6.64 | 6.96 |
| 38004_at | CSPG4 | 4.22E-05 | 0.51 | 5.79 | 6.3 |
| 36725_at | SCN2A | 4.22E-05 | -0.38 | 3.7 | 3.32 |
| 37294_at | BTG1 | 4.23E-05 | 0.72 | 7.21 | 7.93 |
| 41057_at | THEM2 | 4.24E-05 | 0.53 | 4.79 | 5.32 |
| 35843_at | NEK9 | 4.26E-05 | 0.53 | 6.32 | 6.85 |
| 33212_at | RRBP1 | 4.30E-05 | 0.94 | 5.12 | 6.06 |
| 35217_at | MFAP3 | 4.33E-05 | -0.44 | 7.23 | 6.8 |
| 40780_at | CTBP2 | 4.37E-05 | -0.53 | 6.95 | 6.42 |
| 37419_g_at | POU2F2 | 4.38E-05 | -0.61 | 6.89 | 6.27 |
| 190_at | NR4A3 | 4.39E-05 | 0.68 | 4.78 | 5.46 |
| 41655_at | LOC286440 | 4.40E-05 | 0.3 | 5.29 | 5.59 |
| 40050_at | CBFA2T2 | 4.42E-05 | -0.39 | 7.04 | 6.65 |
| 32589_at | CHAF1A | 4.42E-05 | -0.74 | 6.41 | 5.67 |
| 38484_at | SNAP25 | 4.45E-05 | -1.12 | 7.59 | 6.47 |
| 36160_s_at | PTPRN2 | 4.45E-05 | -1.06 | 7.37 | 6.32 |
| 32790_at | NCBP2 | 4.45E-05 | -0.52 | 5.16 | 4.64 |
| 981_at | MCM4 | 4.45E-05 | -0.6 | 5.43 | 4.83 |
| 33823_at | SCARB2 | 4.46E-05 | 0.47 | 5.97 | 6.44 |
| 37005_at | NBL1 | 4.48E-05 | 0.59 | 7.75 | 8.34 |
| 34655_at | MPP2 | 4.51E-05 | -0.38 | 8.03 | 7.65 |
| 36851_g_at | TUSC3 | 4.52E-05 | -0.93 | 6.14 | 5.21 |
| 38799_at | AP1G2 | 4.54E-05 | -0.31 | 9.32 | 9 |
| 35507_at | ARFIP1 | 4.54E-05 | 0.25 | 3.52 | 3.77 |
| 34019_at | CHRNA3 | 4.56E-05 | -1.33 | 6.7 | 5.37 |
| 37152_at | PPARD | 4.57E-05 | 0.26 | 5.86 | 6.12 |
| 39822_s_at | GADD45B | 4.57E-05 | 0.78 | 6.39 | 7.18 |
| 32627_at | RANBP6 | 4.64E-05 | -0.3 | 5.93 | 5.63 |
| 32813_s_at | KATNB1 | 4.64E-05 | -0.34 | 6.97 | 6.63 |
| 41258_at | NSUN5C | 4.65E-05 | -0.7 | 8.75 | 8.05 |
| 38311_at | TGIF2 | 4.66E-05 | 0.66 | 5.68 | 6.34 |
| 35454_at | PLCH2 | 4.70E-05 | -0.45 | 5.32 | 4.88 |
| 36112_r_at | SFRS2 | 4.70E-05 | -0.74 | 7.1 | 6.36 |
| 35869_at | LY86 | 4.70E-05 | 0.46 | 5.79 | 6.25 |
| 33729_at | SLC25A14 | 4.74E-05 | -0.28 | 4.96 | 4.68 |
| 33135_at | SLC19A1 | 4.78E-05 | -0.31 | 6.84 | 6.53 |
| 36514_at | CGRRF1 | 4.78E-05 | 0.57 | 3.79 | 4.36 |
| 41656_at | NMT2 | 4.86E-05 | 0.47 | 5.51 | 5.98 |
| 975_at | PLK4 | 4.88E-05 | -0.53 | 3.95 | 3.42 |
| 40499_r_at | TUSC4 | 4.91E-05 | -0.3 | 6.26 | 5.96 |
| 39846_at | CTSF | 4.94E-05 | 0.36 | 6.92 | 7.28 |
| 35362_at | MYO10 | 4.94E-05 | 0.66 | 3.93 | 4.59 |
| 40575_at | DLG5 | 4.95E-05 | -0.7 | 6.73 | 6.04 |
| 36670_at | EDC4 | 5.00E-05 | -0.24 | 7.68 | 7.44 |
| 37517_at | GARNL4 | 5.00E-05 | -0.54 | 9.13 | 8.59 |
| 399_at | STK25 | 5.01E-05 | -0.44 | 6.95 | 6.51 |
| 38134_at | PLAG1 | 5.05E-05 | 0.37 | 2.78 | 3.16 |
| 35228_at | CPT1B | 5.05E-05 | -0.61 | 6.95 | 6.34 |
| 31795_at | ADRBK2 | 5.09E-05 | -0.44 | 5.73 | 5.29 |
| 37759_at | LAPTM5 | 5.09E-05 | 1.01 | 7.46 | 8.46 |
| 35269_at | HSPBP1 | 5.09E-05 | -0.29 | 7.78 | 7.49 |
| 37282_at | MAD2L1 | 5.10E-05 | -0.93 | 3.45 | 2.52 |
| 35938_at | PLA2G4A | 5.11E-05 | 0.31 | 2.6 | 2.91 |
| 1711_at | TP53BP1 | 5.11E-05 | -0.34 | 7.38 | 7.04 |
| 37937_at | NLE1 | 5.13E-05 | -0.26 | 5.54 | 5.28 |
| 38073_at | RNMT | 5.15E-05 | -0.8 | 6 | 5.2 |
| 41029_at | U1SNRNPBP | 5.21E-05 | -0.28 | 5.67 | 5.4 |
| 37731_at | EPS15 | 5.21E-05 | 0.81 | 6.23 | 7.04 |
| 39340_at | HEXA | 5.23E-05 | 0.3 | 7.48 | 7.78 |
| 37475_at | WDR62 | 5.23E-05 | -0.29 | 8.06 | 7.77 |
| 39732_at | MAP7 | 5.25E-05 | -0.69 | 4.57 | 3.88 |
| 38915_at | LRRC37A2 | 5.26E-05 | -0.58 | 6.99 | 6.41 |
| 35572_f_at | ZNF253 | 5.27E-05 | -0.57 | 4.57 | 4 |
| 32355_at | TANC2 | 5.31E-05 | -0.58 | 5.28 | 4.7 |
| 37993_at | ATP5D | 5.31E-05 | -0.22 | 8.47 | 8.25 |
| 36223_at | SFPQ | 5.31E-05 | -0.55 | 4.43 | 3.87 |
| 39129_at | FLJ41352 | 5.33E-05 | -0.61 | 7.46 | 6.84 |
| 41702_r_at | POLR3F | 5.36E-05 | -0.33 | 3.48 | 3.15 |
| 40937_at | SCAPER | 5.37E-05 | -0.39 | 5.62 | 5.23 |
| 35707_at | LOC89944 | 5.40E-05 | -0.56 | 7.33 | 6.77 |
| 34783_s_at | BUB3 | 5.40E-05 | -0.52 | 6.67 | 6.15 |
| 35399_at | MOCS3 | 5.42E-05 | -0.22 | 4.48 | 4.26 |
| 33767_at | NEFH | 5.43E-05 | 1.44 | 5.62 | 7.06 |
| 33697_at | P2RX7 | 5.47E-05 | 0.24 | 5.82 | 6.06 |
| 1157_s_at | IFNAR1 | 5.49E-05 | 0.35 | 4.57 | 4.92 |
| 39293_at | B3GALNT1 | 5.50E-05 | -0.38 | 3.21 | 2.83 |
| 32223_at | SFRS14 | 5.51E-05 | -0.39 | 9.06 | 8.66 |
| 35961_at | --- | 5.53E-05 | -1.04 | 5.91 | 4.87 |
| 31524_f_at | HIST1H2BI | 5.55E-05 | -0.41 | 5.97 | 5.56 |
| 40180_at | IRS2 | 5.58E-05 | -0.36 | 6.59 | 6.23 |
| 40176_at | TRIM27 | 5.61E-05 | -0.22 | 6.47 | 6.24 |
| 1379_at | EPHA2 | 5.62E-05 | 0.24 | 5.86 | 6.09 |
| 40019_at | EVI2B | 5.63E-05 | 0.82 | 3.68 | 4.5 |
| 34844_at | ZNF516 | 5.64E-05 | 0.31 | 4.06 | 4.37 |
| 41744_at | OPTN | 5.68E-05 | 0.73 | 6.29 | 7.02 |
| 39042_at | TG | 5.68E-05 | -0.65 | 6.99 | 6.33 |
| 34841_at | EIF3C | 5.70E-05 | -0.57 | 9.76 | 9.18 |
| 37542_at | LHFPL2 | 5.71E-05 | 1.05 | 6.87 | 7.92 |
| 33318_at | MBNL2 | 5.72E-05 | 0.38 | 3.11 | 3.49 |
| 37224_at | COX10 | 5.72E-05 | -0.2 | 4.89 | 4.69 |
| 39650_s_at | PCNXL2 | 5.72E-05 | -0.46 | 6.55 | 6.08 |
| 589_at | SRD5A1 | 5.75E-05 | -0.46 | 5.97 | 5.51 |
| 36498_at | AP1S1 | 5.77E-05 | -0.38 | 7.03 | 6.65 |
| 41232_at | TCTN3 | 5.81E-05 | 0.26 | 3.83 | 4.09 |
| 40411_at | NCOA6 | 5.85E-05 | -0.52 | 6.96 | 6.44 |
| 36571_at | TOP2B | 5.86E-05 | -0.46 | 7.84 | 7.38 |
| 38678_at | SNRPE | 5.87E-05 | -0.7 | 5.64 | 4.94 |
| 1725_s_at | --- | 5.94E-05 | -0.63 | 7.15 | 6.53 |
| 36671_at | ASNS | 5.94E-05 | -0.73 | 7.06 | 6.32 |
| 32184_at | LMO2 | 5.97E-05 | 0.95 | 4.58 | 5.53 |
| 31637_s_at | NR1D1 | 5.98E-05 | 0.32 | 8.51 | 8.83 |
| 1217_g_at | PRKCB1 | 6.01E-05 | -0.75 | 4.86 | 4.11 |
| 1084_at | ABL2 | 6.02E-05 | 0.32 | 5.4 | 5.72 |
| 37311_at | TALDO1 | 6.06E-05 | 0.38 | 8.01 | 8.39 |
| 37351_at | UPP1 | 6.08E-05 | 0.79 | 7 | 7.79 |
| 1561_at | DUSP8 | 6.12E-05 | -0.68 | 7.64 | 6.95 |
| 1336_s_at | PRKCB1 | 6.19E-05 | -0.71 | 5.86 | 5.15 |
| 39051_at | NNAT | 6.21E-05 | -1.41 | 9.59 | 8.18 |
| 584_s_at | XRCC5 | 6.22E-05 | -0.67 | 7.09 | 6.42 |
| 410_s_at | CSNK2B | 6.22E-05 | -0.44 | 9.24 | 8.8 |
| 38504_at | CAPN5 | 6.22E-05 | 0.31 | 5.8 | 6.11 |
| 41085_at | POLE2 | 6.25E-05 | -0.31 | 3.7 | 3.4 |
| 36509_at | PIK3C2A | 6.25E-05 | 0.37 | 3.86 | 4.23 |
| 40980_at | CHD8 | 6.25E-05 | -0.36 | 7.63 | 7.26 |
| 41861_at | NOC2L | 6.29E-05 | -0.42 | 6.55 | 6.13 |
| 41800_s_at | DNAJC7 | 6.32E-05 | -0.53 | 7.69 | 7.16 |
| 40810_at | SMARCC1 | 6.37E-05 | -0.52 | 7.29 | 6.77 |
| 1319_at | DDR2 | 6.38E-05 | 0.81 | 5.1 | 5.92 |
| 34973_at | ATP8A1 | 6.38E-05 | -0.41 | 3.21 | 2.8 |
| 36340_at | ZKSCAN1 | 6.39E-05 | -0.39 | 6.37 | 5.98 |
| 32031_at | CAD | 6.39E-05 | -0.25 | 7.6 | 7.35 |
| 34835_at | NCSTN | 6.40E-05 | 0.35 | 8.57 | 8.92 |
| 1599_at | CDKN3 | 6.42E-05 | -0.8 | 5.37 | 4.57 |
| 33635_at | GNA13 | 6.44E-05 | -0.38 | 4.75 | 4.37 |
| 348_at | KIFC1 | 6.47E-05 | -0.51 | 7.46 | 6.95 |
| 32734_at | PPP2R5E | 6.49E-05 | -0.66 | 5.69 | 5.04 |
| 39354_at | PRDX6 | 6.58E-05 | 0.7 | 5.62 | 6.32 |
| 38679_g_at | SNRPE | 6.58E-05 | -0.8 | 8.9 | 8.1 |
| 38878_at | SMAP1 | 6.61E-05 | -0.26 | 5.66 | 5.4 |
| 32397_r_at | ERCC2 | 6.61E-05 | -0.29 | 6.64 | 6.35 |
| 41388_at | MEIS2 | 6.62E-05 | -1.06 | 6.32 | 5.26 |
| 38571_at | FGFR1OP | 6.63E-05 | -0.29 | 6.35 | 6.06 |
| 37658_at | GAS6 | 6.67E-05 | 0.47 | 7.32 | 7.79 |
| 41387_r_at | JMJD3 | 6.76E-05 | -0.29 | 7.9 | 7.61 |
| 36587_at | EEF2 | 6.76E-05 | -0.56 | 10.81 | 10.25 |
| 39431_at | NPEPPS | 6.76E-05 | -0.67 | 6.88 | 6.21 |
| 1584_at | CDC25C | 6.77E-05 | -0.39 | 4.76 | 4.37 |
| 967_g_at | RAD54L | 6.77E-05 | -0.42 | 6.95 | 6.53 |
| 39295_s_at | SORBS2 | 6.77E-05 | -1.25 | 6.54 | 5.29 |
| 34206_at | CENTD2 | 6.78E-05 | 0.29 | 7.17 | 7.46 |
| 39667_at | NOVA2 | 6.87E-05 | -0.29 | 7.13 | 6.84 |
| 31403_at | SLC18A1 | 6.88E-05 | -1.12 | 5.17 | 4.05 |
| 32081_at | CIT | 6.95E-05 | 0.57 | 4.97 | 5.54 |
| 38633_at | MTA1 | 7.00E-05 | -0.81 | 7.59 | 6.78 |
| 40292_at | DBC1 | 7.00E-05 | -0.42 | 4.52 | 4.1 |
| 41403_at | SNRPF | 7.02E-05 | -0.73 | 7.35 | 6.62 |
| 34649_at | SSR1 | 7.07E-05 | 0.38 | 4.51 | 4.89 |
| 37335_at | DGUOK | 7.08E-05 | -0.33 | 6.96 | 6.63 |
| 31538_at | LOC643779 | 7.09E-05 | -0.56 | 11.14 | 10.58 |
| 37098_at | PPOX | 7.12E-05 | -0.34 | 7.32 | 6.98 |
| 35853_at | PICK1 | 7.15E-05 | -0.2 | 7.75 | 7.55 |
| 37806_at | SHANK2 | 7.18E-05 | -0.54 | 4.2 | 3.66 |
| 38455_at | SNRPB | 7.19E-05 | -0.72 | 8.86 | 8.14 |
| 1979_s_at | NOL1 | 7.20E-05 | -0.38 | 5.93 | 5.55 |
| 39522_at | PFKFB3 | 7.21E-05 | 0.36 | 6.56 | 6.92 |
| 1433_g_at | SMAD3 | 7.21E-05 | 0.34 | 4.47 | 4.81 |
| 36051_s_at | ADD2 | 7.22E-05 | -0.27 | 6.81 | 6.54 |
| 33756_at | AOC3 | 7.25E-05 | 0.44 | 4.61 | 5.04 |
| 1867_at | CFLAR | 7.30E-05 | 0.36 | 5.19 | 5.55 |
| 34698_at | GNAO1 | 7.35E-05 | -0.82 | 7 | 6.18 |
| 31799_at | --- | 7.41E-05 | -0.23 | 4.74 | 4.51 |
| 39700_at | ARHGAP1 | 7.46E-05 | 0.41 | 8.46 | 8.88 |
| 34244_r_at | L3MBTL | 7.47E-05 | -0.29 | 2.89 | 2.6 |
| 39746_at | POLR2B | 7.51E-05 | -0.51 | 6.84 | 6.33 |
| 38181_at | MMP11 | 7.52E-05 | -0.31 | 6.41 | 6.09 |
| 41491_s_at | ATP11A | 7.54E-05 | -0.46 | 6.78 | 6.32 |
| 40195_at | H2AFX | 7.57E-05 | -0.7 | 7.15 | 6.45 |
| 38107_at | UNC119 | 7.57E-05 | -0.26 | 7.1 | 6.84 |
| 38798_s_at | AP1G2 | 7.59E-05 | -0.64 | 7.15 | 6.51 |
| 35930_at | LOC728395 | 7.60E-05 | -0.31 | 5.78 | 5.47 |
| 41862_at | NCAPD3 | 7.61E-05 | -0.3 | 5 | 4.7 |
| 40259_at | RBM9 | 7.71E-05 | -0.47 | 4.67 | 4.2 |
| 33911_at | PTPRD | 7.72E-05 | -0.94 | 5.28 | 4.34 |
| 39024_at | NUP98 | 7.73E-05 | 0.33 | 7.09 | 7.42 |
| 653_at | RFC5 | 7.74E-05 | -0.45 | 5.17 | 4.72 |
| 40160_at | LOC100101267 | 7.80E-05 | -0.67 | 8.03 | 7.36 |
| 34940_at | --- | 7.83E-05 | 0.22 | 2.55 | 2.76 |
| 41743_i_at | OPTN | 7.89E-05 | 0.74 | 4.81 | 5.55 |
| 32865_at | --- | 7.90E-05 | 0.32 | 3.24 | 3.56 |
| 35209_at | EPM2AIP1 | 7.91E-05 | -0.45 | 5.6 | 5.15 |
| 37173_at | CENPE | 7.99E-05 | -0.47 | 4.69 | 4.22 |
| 38561_at | --- | 8.00E-05 | -0.38 | 5.2 | 4.82 |
| 37913_at | DHFR | 8.06E-05 | -0.47 | 6.7 | 6.23 |
| 41082_at | RND2 | 8.08E-05 | -0.44 | 5.54 | 5.1 |
| 1843_at | --- | 8.08E-05 | -0.25 | 6.64 | 6.39 |
| 39242_at | SYT5 | 8.18E-05 | -0.55 | 7.91 | 7.36 |
| 36740_at | DOC2A | 8.18E-05 | -0.31 | 7 | 6.7 |
| 33330_at | CLTCL1 | 8.18E-05 | -0.3 | 6.17 | 5.87 |
| 35076_at | --- | 8.19E-05 | -0.49 | 4.93 | 4.44 |
| 37750_at | UBE2V2 | 8.24E-05 | -0.41 | 5.51 | 5.1 |
| 40833_r_at | TOR1AIP1 | 8.25E-05 | 0.2 | 2.37 | 2.57 |
| 37124_i_at | CYP3A43 | 8.26E-05 | -0.79 | 4.89 | 4.1 |
| 35351_at | RBM4 | 8.28E-05 | -0.55 | 8.3 | 7.75 |
| 36476_at | BRD8 | 8.34E-05 | -0.47 | 5.3 | 4.84 |
| 241_g_at | SRM | 8.34E-05 | 0.34 | 8.76 | 9.1 |
| 34888_at | HEXB | 8.37E-05 | 0.29 | 3.15 | 3.43 |
| 34213_at | WWC1 | 8.40E-05 | -0.48 | 5.79 | 5.32 |
| 33305_at | SERPINB1 | 8.43E-05 | 0.71 | 5.08 | 5.79 |
| 35831_at | ATP9A | 8.56E-05 | -0.94 | 8.71 | 7.77 |
| 234_s_at | PTN | 8.60E-05 | 0.96 | 6.38 | 7.35 |
| 34414_at | KIAA0368 | 8.68E-05 | -0.66 | 4.54 | 3.88 |
| 39744_at | DDX3X | 8.70E-05 | 0.53 | 6.53 | 7.06 |
| 36544_at | ARL3 | 8.71E-05 | -0.53 | 6.72 | 6.19 |
| 32980_f_at | HIST1H2BC | 8.72E-05 | -0.33 | 6.21 | 5.88 |
| 34531_at | FLRT1 | 8.77E-05 | 0.69 | 6.8 | 7.49 |
| 40587_s_at | EEF1E1 | 8.94E-05 | -0.42 | 7.49 | 7.07 |
| 32574_at | SMPD1 | 8.98E-05 | 0.56 | 6.47 | 7.03 |
| 34717_s_at | FUSIP1 | 8.98E-05 | -0.52 | 4.08 | 3.56 |
| 38074_at | AP3S1 | 8.99E-05 | 0.58 | 6.95 | 7.53 |
| 38773_at | CBR1 | 9.04E-05 | 0.31 | 5.69 | 6 |
| 37550_at | F8 | 9.20E-05 | 0.28 | 4.6 | 4.88 |
| 1061_at | IL10RA | 9.22E-05 | 0.44 | 6.14 | 6.57 |
| 35870_at | AASS | 9.26E-05 | 0.31 | 3.83 | 4.14 |
| 34769_at | FAAH | 9.40E-05 | -0.26 | 6.87 | 6.61 |
| 41649_at | FOXJ2 | 9.42E-05 | 0.37 | 7.29 | 7.67 |
| 36110_at | RAB5A | 9.42E-05 | 0.55 | 6.74 | 7.3 |
| 1439_s_at | MAPKAPK2 | 9.42E-05 | 0.46 | 5.2 | 5.66 |
| 37296_at | ARL1 | 9.46E-05 | 0.4 | 4.06 | 4.46 |
| 236_at | GNAO1 | 9.47E-05 | -0.57 | 5.83 | 5.26 |
| 38632_at | DNAJB5 | 9.50E-05 | -0.42 | 7.17 | 6.75 |
| 41605_at | KIAA1219 | 9.53E-05 | -0.23 | 4.15 | 3.92 |
| 40758_at | ICT1 | 9.54E-05 | -0.48 | 5.57 | 5.09 |
| 33111_at | CD160 | 9.56E-05 | -0.38 | 6.26 | 5.88 |
| 454_at | SMARCD1 | 9.58E-05 | -0.34 | 7.4 | 7.06 |
| 32039_at | AP3B1 | 9.62E-05 | 0.41 | 4.77 | 5.18 |
| 38258_at | --- | 9.65E-05 | -0.23 | 6.36 | 6.13 |
| 40169_at | M6PRBP1 | 9.74E-05 | 0.39 | 6.73 | 7.12 |
| 37470_at | LAIR1 | 9.78E-05 | 0.31 | 6.18 | 6.49 |
| 553_g_at | ARHGAP1 | 9.79E-05 | 0.69 | 7.92 | 8.61 |
| 32856_at | LOC731210 | 9.83E-05 | 0.45 | 7.12 | 7.56 |
| 39585_at | CCRK | 9.87E-05 | -0.28 | 5.78 | 5.5 |
| 36061_at | SEMA5A | 9.89E-05 | 0.53 | 6.25 | 6.78 |
| 1875_f_at | PMS2L3 | 9.89E-05 | -0.4 | 6.23 | 5.83 |
| 35127_at | HIST1H2AE | 9.94E-05 | -0.7 | 5.42 | 4.72 |
| 41632_at | E2F3 | 9.94E-05 | -0.6 | 4.52 | 3.93 |
| 40359_at | RASSF7 | 9.94E-05 | -0.23 | 8.66 | 8.43 |
