# Supplementary material for: Enteric Neural Crest Differentiation in Ganglioneuromas Implicates Hedgehog Signaling in Peripheral Neuroblastic Tumor Pathogenesis
Source: PLoS One. 2009 Oct 16;4(10):e7491. doi: 10.1371/journal.pone.0007491 (PMC2759000; doi:10.1371/journal.pone.0007491)
Supplement: Table S2 — Genes differentially expressed in SY5Y cells tranduced with Gli1 and GFP or GFP only. Mean values are computed from log2 transformed expression values. The difference in log2 transformed means is therefore equal to log2 (fold change). (0.33 MB DOC) [file pone.0007491.s002.doc]

**Supplemental Table 2** Genes differentially expressed in SY5Y cells tranduced with *Gli1* and *GFP* or *GFP* only. Mean values are computed from log2 transformed expression values. The difference in log2 transformed means is therefore equal to log2 (fold change).

| ProbeSetID | GeneSymbol | Pvalue: GLI - GFP | Diff: GLI-GFP | GFP mean | Gli1 mean |
| --- | --- | --- | --- | --- | --- |
| 202350_s_at | MATN2 | 2.80E-12 | 3.79 | 4.84 | 8.63 |
| 205857_at | --- | 4.90E-12 | 4.11 | 5.46 | 9.57 |
| 205206_at | KAL1 | 2.86E-11 | 3.74 | 5.67 | 9.41 |
| 218718_at | PDGFC | 3.38E-10 | 3.99 | 5.21 | 9.2 |
| 218501_at | ARHGEF3 | 1.46E-09 | 2.24 | 6.43 | 8.67 |
| 201341_at | ENC1 | 1.87E-09 | 2.11 | 9.45 | 11.56 |
| 210302_s_at | MAB21L2 | 2.80E-09 | 1.57 | 6.66 | 8.24 |
| 208188_at | KRT9 | 3.55E-09 | 1.65 | 6.95 | 8.6 |
| 210139_s_at | PMP22 | 4.73E-09 | 1.51 | 8.41 | 9.93 |
| 206089_at | NELL1 | 6.96E-09 | -1.65 | 9.79 | 8.14 |
| 207373_at | HOXD10 | 8.66E-09 | 1.36 | 4.82 | 6.18 |
| 210258_at | RGS13 | 9.19E-09 | 2.21 | 6.04 | 8.25 |
| 209815_at | PTCH | 1.27E-08 | 2.03 | 9.98 | 12.01 |
| 204464_s_at | EDNRA | 2.22E-08 | 1.89 | 7.22 | 9.11 |
| 202409_at | LOC492304 | 2.23E-08 | -2.01 | 9.8 | 7.79 |
| 202087_s_at | CTSL | 2.46E-08 | 1.45 | 8.68 | 10.14 |
| 205651_x_at | RAPGEF4 | 2.91E-08 | 1.41 | 5.99 | 7.4 |
| 209816_at | PTCH | 2.95E-08 | 1.72 | 6.3 | 8.02 |
| 201340_s_at | ENC1 | 3.27E-08 | 2.77 | 5.33 | 8.11 |
| 211959_at | IGFBP5 | 4.06E-08 | 1.63 | 9.86 | 11.5 |
| 210095_s_at | IGFBP3 | 4.26E-08 | 1.69 | 8.36 | 10.05 |
| 216268_s_at | JAG1 | 4.65E-08 | 1.84 | 6.6 | 8.45 |
| 202363_at | SPOCK | 6.29E-08 | -1.27 | 10.41 | 9.14 |
| 218559_s_at | MAFB | 6.64E-08 | 1.36 | 7.24 | 8.59 |
| 209875_s_at | SPP1 | 7.81E-08 | 1.59 | 5.98 | 7.57 |
| 202723_s_at | FOXO1A | 8.14E-08 | 1.73 | 6.15 | 7.87 |
| 204463_s_at | EDNRA | 8.19E-08 | 1.46 | 4.87 | 6.33 |
| 210319_x_at | MSX2 | 8.96E-08 | 1.45 | 6.05 | 7.5 |
| 222258_s_at | SH3BP4 | 1.06E-07 | 1.07 | 7.82 | 8.9 |
| 209099_x_at | JAG1 | 1.27E-07 | 1.45 | 7.18 | 8.63 |
| 221796_at | NTRK2 | 1.27E-07 | 1.99 | 4.84 | 6.83 |
| 207173_x_at | CDH11 | 1.36E-07 | 1.51 | 7.67 | 9.18 |
| 210675_s_at | PTPRR | 1.37E-07 | 1.03 | 6.71 | 7.74 |
| 211421_s_at | RET | 1.47E-07 | 1.19 | 10.71 | 11.9 |
| 219669_at | PRV1 | 1.49E-07 | 1.96 | 6.45 | 8.42 |
| 203706_s_at | FZD7 | 1.51E-07 | 1.57 | 7.37 | 8.94 |
| 201939_at | PLK2 | 1.59E-07 | 1.32 | 7.56 | 8.87 |
| 205604_at | HOXD9 | 1.70E-07 | 1.32 | 8 | 9.32 |
| 211006_s_at | KCNB1 | 2.32E-07 | 1.05 | 7.52 | 8.57 |
| 221584_s_at | KCNMA1 | 2.87E-07 | -1.18 | 8.51 | 7.32 |
| 210078_s_at | KCNAB1 | 3.29E-07 | 1.7 | 5.13 | 6.83 |
| 201983_s_at | EGFR | 3.72E-07 | 1.49 | 6.73 | 8.22 |
| 205357_s_at | AGTR1 | 4.04E-07 | -1.81 | 7.72 | 5.91 |
| 202016_at | MEST | 4.05E-07 | 1.46 | 6.02 | 7.49 |
| 201250_s_at | SLC2A1 | 4.25E-07 | 1.03 | 8.27 | 9.3 |
| 208606_s_at | WNT4 | 4.52E-07 | 2.26 | 5.12 | 7.38 |
| 214961_at | KIAA0774 | 4.55E-07 | 0.94 | 4.62 | 5.56 |
| 202935_s_at | SOX9 | 5.11E-07 | 1.13 | 5.03 | 6.16 |
| 202284_s_at | CDKN1A | 5.31E-07 | 0.96 | 9.19 | 10.15 |
| 219414_at | CLSTN2 | 5.39E-07 | -0.88 | 7.42 | 6.55 |
| 201739_at | SGK | 6.84E-07 | 0.93 | 10.32 | 11.24 |
| 209325_s_at | RGS16 | 6.86E-07 | 0.93 | 8.02 | 8.95 |
| 213413_at | SBLF | 7.12E-07 | 1.11 | 6.02 | 7.13 |
| 212143_s_at | IGFBP3 | 7.20E-07 | 1.22 | 8.03 | 9.25 |
| 213960_at | --- | 7.53E-07 | 0.91 | 5.98 | 6.89 |
| 212192_at | KCTD12 | 8.06E-07 | 1.2 | 9.53 | 10.72 |
| 207172_s_at | CDH11 | 8.58E-07 | 0.85 | 7.04 | 7.89 |
| 215117_at | RAG2 | 9.36E-07 | 1.24 | 3.89 | 5.13 |
| 205721_at | GFRA2 | 1.00E-06 | 1.51 | 7.12 | 8.63 |
| 213802_at | PRSS12 | 1.03E-06 | 1.21 | 6.97 | 8.19 |
| 201302_at | ANXA4 | 1.10E-06 | 0.78 | 7.6 | 8.38 |
| 203180_at | ALDH1A3 | 1.33E-06 | 1.06 | 6.96 | 8.02 |
| 204529_s_at | TOX | 1.36E-06 | -0.83 | 10.07 | 9.24 |
| 202149_at | NEDD9 | 1.52E-06 | 1.5 | 7.66 | 9.16 |
| 219908_at | DKK2 | 1.64E-06 | 1.12 | 4.43 | 5.55 |
| 217764_s_at | RAB31 | 1.64E-06 | 0.81 | 8.66 | 9.47 |
| 213139_at | SNAI2 | 1.88E-06 | 1.14 | 7.04 | 8.18 |
| 206167_s_at | ARHGAP6 | 1.93E-06 | 1.32 | 5.02 | 6.34 |
| 209443_at | SERPINA5 | 1.95E-06 | 0.83 | 7.4 | 8.23 |
| 212706_at | RASA4 | 2.02E-06 | 0.88 | 8.22 | 9.1 |
| 208636_at | ACTN1 | 2.03E-06 | 0.86 | 9.89 | 10.75 |
| 203705_s_at | FZD7 | 2.22E-06 | 1.06 | 7.12 | 8.18 |
| 209324_s_at | RGS16 | 2.48E-06 | 1 | 8.11 | 9.11 |
| 207761_s_at | DKFZP586A0522 | 2.54E-06 | 0.87 | 9.14 | 10.01 |
| 210511_s_at | INHBA | 2.92E-06 | -0.95 | 8.58 | 7.63 |
| 202575_at | CRABP2 | 3.04E-06 | 1.09 | 10.09 | 11.18 |
| 205515_at | PRSS12 | 3.33E-06 | 0.83 | 6.55 | 7.39 |
| 203633_at | --- | 3.38E-06 | 0.76 | 9.08 | 9.84 |
| 205934_at | PLCL1 | 3.38E-06 | -1.13 | 6.84 | 5.71 |
| 218764_at | PRKCH | 3.60E-06 | 0.99 | 7.61 | 8.6 |
| 202936_s_at | SOX9 | 3.66E-06 | 2.03 | 6.27 | 8.3 |
| 203857_s_at | PDIA5 | 3.79E-06 | 0.73 | 7.95 | 8.69 |
| 210986_s_at | TPM1 | 4.06E-06 | 0.8 | 10.14 | 10.94 |
| 214479_at | GFRA3 | 4.24E-06 | -0.94 | 7.72 | 6.78 |
| 205802_at | TRPC1 | 4.25E-06 | 1.06 | 8.18 | 9.23 |
| 200839_s_at | CTSB | 4.28E-06 | 0.86 | 9.52 | 10.38 |
| 204035_at | SCG2 | 4.32E-06 | 0.81 | 9.38 | 10.19 |
| 205697_at | SCGN | 4.53E-06 | 0.81 | 7.38 | 8.19 |
| 208782_at | FSTL1 | 4.64E-06 | 0.74 | 9.81 | 10.54 |
| 201426_s_at | VIM | 4.86E-06 | 0.9 | 11.27 | 12.17 |
| 207069_s_at | SMAD6 | 5.04E-06 | 1.33 | 5.93 | 7.26 |
| 222154_s_at | DNAPTP6 | 5.15E-06 | 0.77 | 9.21 | 9.98 |
| 215465_at | ABCA12 | 5.20E-06 | -0.89 | 8.1 | 7.21 |
| 205522_at | HOXD4 | 5.41E-06 | 1.28 | 8.02 | 9.3 |
| 202724_s_at | FOXO1A | 5.45E-06 | 1.15 | 5.65 | 6.79 |
| 219926_at | POPDC3 | 5.45E-06 | 0.85 | 8.73 | 9.58 |
| 213880_at | LGR5 | 5.53E-06 | 0.78 | 9.36 | 10.14 |
| 204686_at | IRS1 | 5.60E-06 | 0.83 | 6.49 | 7.33 |
| 212915_at | PDZRN3 | 5.62E-06 | 1.07 | 7.59 | 8.66 |
| 203131_at | PDGFRA | 5.67E-06 | -0.73 | 10.76 | 10.03 |
| 209560_s_at | DLK1 | 5.73E-06 | -0.81 | 13.39 | 12.58 |
| 205350_at | CRABP1 | 5.91E-06 | 0.85 | 8.68 | 9.53 |
| 219682_s_at | TBX3 | 6.13E-06 | 0.71 | 10.27 | 10.98 |
| 205803_s_at | TRPC1 | 6.61E-06 | 0.97 | 7.91 | 8.88 |
| 221577_x_at | GDF15 | 7.44E-06 | 1.11 | 6.6 | 7.71 |
| 203329_at | PTPRM | 7.44E-06 | -0.66 | 7.63 | 6.97 |
| 218087_s_at | SORBS1 | 7.61E-06 | 1.33 | 6.29 | 7.63 |
| 214604_at | HOXD11 | 7.82E-06 | 1.02 | 5.63 | 6.65 |
| 212423_at | C10orf56 | 8.04E-06 | 0.63 | 7.7 | 8.33 |
| 203297_s_at | JARID2 | 8.37E-06 | 0.67 | 9.58 | 10.24 |
| 207826_s_at | ID3 | 8.50E-06 | 0.84 | 8.91 | 9.76 |
| 212148_at | PBX1 | 8.55E-06 | 0.72 | 9.19 | 9.92 |
| 213260_at | FOXC1 | 8.64E-06 | 0.69 | 8.31 | 9 |
| 210547_x_at | ICA1 | 8.77E-06 | -0.66 | 11.17 | 10.52 |
| 202202_s_at | LAMA4 | 8.89E-06 | 0.96 | 5.14 | 6.1 |
| 202007_at | NID | 9.42E-06 | 0.83 | 8.25 | 9.07 |
| 204036_at | EDG2 | 9.69E-06 | 0.7 | 7.19 | 7.89 |
| 203476_at | TPBG | 1.03E-05 | 0.61 | 8.35 | 8.95 |
| 206577_at | VIP | 1.09E-05 | 0.66 | 5.11 | 5.77 |
| 212188_at | KCTD12 | 1.14E-05 | 0.88 | 8.33 | 9.21 |
| 204517_at | PPIC | 1.14E-05 | 0.64 | 7.86 | 8.5 |
| 205517_at | GATA4 | 1.17E-05 | 0.74 | 7.61 | 8.35 |
| 221558_s_at | LEF1 | 1.18E-05 | 0.67 | 8.88 | 9.54 |
| 212403_at | UBE3B | 1.21E-05 | 0.91 | 9.19 | 10.1 |
| 206462_s_at | NTRK3 | 1.25E-05 | 0.79 | 5.45 | 6.24 |
| 208070_s_at | REV3L | 1.30E-05 | 0.94 | 9.76 | 10.7 |
| 208016_s_at | AGTR1 | 1.35E-05 | -0.75 | 6.03 | 5.29 |
| 213689_x_at | LOC388650 | 1.43E-05 | 0.64 | 7.7 | 8.35 |
| 206084_at | PTPRR | 1.44E-05 | 1.19 | 6.2 | 7.39 |
| 218880_at | FOSL2 | 1.45E-05 | 0.76 | 5.56 | 6.32 |
| 203066_at | GALNAC4S-6ST | 1.49E-05 | -0.63 | 8.17 | 7.54 |
| 201010_s_at | TXNIP | 1.59E-05 | 0.61 | 11.05 | 11.66 |
| 203424_s_at | IGFBP5 | 1.63E-05 | 1.08 | 6.35 | 7.43 |
| 201430_s_at | DPYSL3 | 1.65E-05 | -0.98 | 8.87 | 7.88 |
| 209160_at | AKR1C3 | 1.65E-05 | 0.82 | 5.3 | 6.12 |
| 210609_s_at | TP53I3 | 1.69E-05 | 0.72 | 9.09 | 9.81 |
| 213479_at | NPTX2 | 1.71E-05 | 0.73 | 9.61 | 10.34 |
| 201860_s_at | PLAT | 1.76E-05 | 0.57 | 6.32 | 6.9 |
| 207949_s_at | ICA1 | 1.79E-05 | -0.65 | 11.23 | 10.57 |
| 220794_at | GREM2 | 1.83E-05 | 0.92 | 4.88 | 5.81 |
| 204811_s_at | CACNA2D2 | 1.86E-05 | -0.73 | 10.69 | 9.97 |
| 209056_s_at | CDC5L | 1.96E-05 | 0.6 | 9.84 | 10.44 |
| 204850_s_at | DCX | 2.03E-05 | -0.9 | 11.81 | 10.91 |
| 212554_at | CAP2 | 2.06E-05 | 0.67 | 6.88 | 7.55 |
| 200632_s_at | NDRG1 | 2.23E-05 | 0.91 | 7.25 | 8.17 |
| 203298_s_at | JARID2 | 2.26E-05 | 0.69 | 9.56 | 10.25 |
| 202973_x_at | FAM13A1 | 2.27E-05 | 0.62 | 7.08 | 7.7 |
| 210074_at | CTSL2 | 2.30E-05 | 0.58 | 9.74 | 10.32 |
| 202150_s_at | NEDD9 | 2.31E-05 | 0.74 | 7.44 | 8.17 |
| 219737_s_at | PCDH9 | 2.38E-05 | 0.65 | 8.79 | 9.44 |
| 213931_at | ID2 | 2.41E-05 | 0.76 | 8.73 | 9.49 |
| 203789_s_at | SEMA3C | 2.60E-05 | 0.97 | 8.7 | 9.67 |
| 205198_s_at | ATP7A | 2.63E-05 | 0.71 | 7.42 | 8.13 |
| 203824_at | TSPAN8 | 2.64E-05 | 0.87 | 5.09 | 5.95 |
| 212099_at | RHOB | 2.68E-05 | 0.73 | 9.81 | 10.54 |
| 219671_at | HPCAL4 | 2.74E-05 | 0.68 | 8.17 | 8.85 |
| 219032_x_at | OPN3 | 2.91E-05 | 0.74 | 9.17 | 9.91 |
| 212236_x_at | KRT17 | 2.96E-05 | 1.05 | 6.98 | 8.02 |
| 212225_at | SUI1 | 2.97E-05 | -0.66 | 6.55 | 5.89 |
| 205538_at | CORO2A | 3.03E-05 | 1.03 | 6.81 | 7.83 |
| 213274_s_at | CTSB | 3.16E-05 | 0.55 | 8.49 | 9.04 |
| 201431_s_at | DPYSL3 | 3.19E-05 | -0.57 | 11.16 | 10.59 |
| 213721_at | SOX2 | 3.19E-05 | 0.73 | 5.1 | 5.83 |
| 221204_s_at | CRTAC1 | 3.19E-05 | 0.61 | 7.52 | 8.13 |
| 205150_s_at | KIAA0644 | 3.40E-05 | 0.71 | 7.5 | 8.21 |
| 213381_at | C10orf72 | 3.40E-05 | -0.57 | 6.34 | 5.77 |
| 203787_at | SSBP2 | 3.52E-05 | 0.86 | 7.82 | 8.69 |
| 203962_s_at | NEBL | 3.58E-05 | -0.81 | 6.88 | 6.07 |
| 204678_s_at | KCNK1 | 3.62E-05 | -0.64 | 9.22 | 8.59 |
| 203637_s_at | MID1 | 3.67E-05 | 0.8 | 7.66 | 8.46 |
| 207793_s_at | EPB41 | 3.77E-05 | -0.71 | 5.66 | 4.96 |
| 210829_s_at | SSBP2 | 3.82E-05 | 0.61 | 7.42 | 8.03 |
| 218326_s_at | LGR4 | 3.83E-05 | 1.08 | 4.69 | 5.77 |
| 201688_s_at | TPD52 | 3.83E-05 | -0.73 | 8.49 | 7.75 |
| 209082_s_at | COL18A1 | 3.84E-05 | 0.52 | 6.98 | 7.5 |
| 221530_s_at | BHLHB3 | 3.90E-05 | 0.53 | 4.65 | 5.17 |
| 215771_x_at | RET | 3.98E-05 | 0.86 | 8.53 | 9.39 |
| 208944_at | --- | 3.98E-05 | 0.54 | 6.45 | 6.99 |
| 209191_at | TUBB6 | 4.13E-05 | 0.66 | 8.75 | 9.41 |
| 206116_s_at | TPM1 | 4.22E-05 | 0.66 | 9.16 | 9.82 |
| 219480_at | SNAI1 | 4.42E-05 | 0.58 | 6.27 | 6.86 |
| 203685_at | BCL2 | 4.45E-05 | 0.6 | 9.73 | 10.33 |
| 212642_s_at | HIVEP2 | 4.45E-05 | 0.59 | 7.83 | 8.41 |
| 202289_s_at | TACC2 | 4.47E-05 | -0.61 | 8.79 | 8.18 |
| 220795_s_at | KIAA1446 | 4.51E-05 | -0.76 | 9.52 | 8.77 |
| 217731_s_at | ITM2B | 4.51E-05 | 0.8 | 10.5 | 11.29 |
| 204288_s_at | ARGBP2 | 4.56E-05 | -0.53 | 7.49 | 6.96 |
| 202388_at | RGS2 | 4.82E-05 | 0.67 | 10.36 | 11.04 |
| 212419_at | C10orf56 | 4.88E-05 | 0.64 | 7.44 | 8.08 |
| 218651_s_at | FLJ11196 | 4.89E-05 | 0.61 | 7.45 | 8.06 |
| 201009_s_at | TXNIP | 5.00E-05 | 0.51 | 9.81 | 10.32 |
| 205413_at | C11orf8 | 5.10E-05 | 0.68 | 8.26 | 8.94 |
| 204457_s_at | GAS1 | 5.26E-05 | 1.01 | 7.25 | 8.26 |
| 201073_s_at | SMARCC1 | 5.34E-05 | -0.72 | 10.53 | 9.8 |
| 202601_s_at | HTATSF1 | 5.51E-05 | -0.79 | 8.39 | 7.6 |
| 205701_at | IPO8 | 5.72E-05 | -0.58 | 7.61 | 7.04 |
| 211708_s_at | SCD | 5.75E-05 | -0.78 | 8.9 | 8.13 |
| 211671_s_at | NR3C1 | 5.93E-05 | 0.55 | 6.85 | 7.4 |
| 201566_x_at | ID2 | 6.06E-05 | 0.9 | 9.79 | 10.68 |
| 215076_s_at | COL3A1 | 6.17E-05 | 0.48 | 5.23 | 5.7 |
| 215767_at | C2orf10 | 6.19E-05 | -0.57 | 9.7 | 9.13 |
| 201689_s_at | TPD52 | 6.39E-05 | -0.72 | 8.63 | 7.92 |
| 221760_at | MAN1A1 | 6.44E-05 | 0.7 | 7.12 | 7.82 |
| 212741_at | MAOA | 6.46E-05 | 0.55 | 8.1 | 8.65 |
| 210987_x_at | TPM1 | 6.65E-05 | 0.58 | 10.14 | 10.72 |
| 205151_s_at | KIAA0644 | 6.66E-05 | 0.55 | 8.09 | 8.65 |
| 201906_s_at | CTDSPL | 6.68E-05 | 0.59 | 8.54 | 9.13 |
| 206104_at | ISL1 | 6.71E-05 | -0.47 | 12.52 | 12.04 |
| 211458_s_at | GABARAPL1 | 6.74E-05 | 0.59 | 6.65 | 7.24 |
| 207717_s_at | PKP2 | 6.94E-05 | 1.07 | 4.58 | 5.65 |
| 218980_at | FHOD3 | 7.05E-05 | -0.56 | 9.26 | 8.71 |
| 201578_at | PODXL | 7.14E-05 | -0.47 | 9.27 | 8.8 |
| 201494_at | PRCP | 7.21E-05 | 0.53 | 9.62 | 10.15 |
| 204697_s_at | CHGA | 7.23E-05 | -0.67 | 12.73 | 12.06 |
| 218656_s_at | LHFP | 7.27E-05 | 0.76 | 6.29 | 7.04 |
| 202037_s_at | SFRP1 | 7.45E-05 | 0.6 | 8.2 | 8.8 |
| 221563_at | DUSP10 | 7.73E-05 | 0.77 | 7.26 | 8.03 |
| 201809_s_at | ENG | 7.88E-05 | 0.72 | 8.26 | 8.98 |
| 207074_s_at | SLC18A1 | 8.15E-05 | 0.48 | 7.86 | 8.35 |
| 214416_at | MGC27345 | 8.46E-05 | -0.88 | 5.5 | 4.62 |
| 206662_at | GLRX | 8.66E-05 | 0.51 | 8.06 | 8.57 |
| 219301_s_at | CNTNAP2 | 8.69E-05 | -0.7 | 7.28 | 6.58 |
| 214053_at | ERBB4 | 8.70E-05 | -0.81 | 7.56 | 6.75 |
| 206580_s_at | EFEMP2 | 9.35E-05 | 0.59 | 7.79 | 8.38 |
| 211203_s_at | CNTN1 | 9.71E-05 | -0.79 | 8.52 | 7.73 |
| 217762_s_at | RAB31 | 9.75E-05 | 0.66 | 8.39 | 9.05 |
| 212558_at | SPRY1 | 9.78E-05 | 0.52 | 7.68 | 8.2 |
| 208791_at | CLU | 9.81E-05 | 0.7 | 7.25 | 7.94 |
