# Supplementary material for: Enteric Neural Crest Differentiation in Ganglioneuromas Implicates Hedgehog Signaling in Peripheral Neuroblastic Tumor Pathogenesis
Source: PLoS One. 2009 Oct 16;4(10):e7491. doi: 10.1371/journal.pone.0007491 (PMC2759000; doi:10.1371/journal.pone.0007491)
Supplement: Table S3 — Primers and antibodies used (0.03 MB DOC) [file pone.0007491.s003.doc]

**Supplemental Table 3.**

**Primers:**

**Gene forward primer reverse primer**

MATN2 CCACATGAAATACATGGGAA CCCAACAGCATACATAGTGA

KAL1 GCTGGAGGTTAAGTGGTCCT GAAACTGGTACCATCGGCTG

PMP22 CTCCACGATCGTCAGCCAAT AGGGTGAAGAGTTGGCAGAA

IGFBP5 ATGGAGGCTTCCCTGCAGGA TTGTCCACGCACCAGCAGAT

NTRK2 TCATGGGCTGGCCTGGAATT ACGCAATCACCACCACAGCA

FDZ7 TTCTACGAGCAGGCCTTCCG AGTCTGTGGTAGAAGCGGCG

EGFR AACCAGTGTGCTGCAGGCTG GCCGTGATCTGTCACCACAT

WNT4a AAGGTGGTGACGCAAGGGAC GCGATGTTGTCAGAGCATCC

SOX2 GCAGCACCCGGGCCTCAATG TGGAGCCAAGAGCCATGCCA

EDNRA GTTTTCTGCTTGGTTGTAAT CTATGGGGTTTATACATGAA

RET AGTTCAAGCGGAAGGAGGAC CGGTTGAGAACCAGCCTATA

VMAT2 CCCGTACATCCTCATTGCTG CACAAAGCCACCTCCCCATT

CHGB CTCGTCTTCAGGACTTGGCG TACATGCGCAGAATGGTGGC

DBH AGATCGTGAACCAGGACAAT TTGACACACATCTCCTCCAG

TH TGGAGTACTTCGTGCGCCTCG CGAGAAGCCCGGGTGGTCCA

NP-Y CGACTGGGGCTGTCCGGACT GTGTCTCTGGGCTGGATCGT

TFAP2B AGAAGTTCAGAGACGGCTGTC GGAAACTCCGTTTCGCAAAT

CGRP TGCAGCACCATTCAGGTCTG GCTTAGATCTGGGGCTGTCC

VIP AGACTTCGGCATGGCCTCTT GGCAGAAAGTTGACCCAAGA

GFAP CGCCACTTGCAGGAGTACCA CTCTCCATCCCGCATCTCCA

EDNRB CACCGACAACTCCATCAAGC ACGGTGGGTACATGGCAAAC

GLI1 CCCCACCAGAGAATGG GATGCTGGGGGCTG

GLI3 AAGACTGACCACCAGGGCTTG GGCTTTGAAGGGTTTCTGCT

DHH AAGGATGAGGAGAACAGTGG TAGTGATGTCCAAAGCACGG

IGFBP6 TGCGGGTGTCCAAGACACTG ATCCACACACCAGCAGGGAC

**Primary antibodies:**

**Antibody dilution catalog# Supplier**

anti-CGRP 1:400 C9487 Sigma

anti-GFAP 1:1,000 PU020-UP BioGenex

anti-TH 1:400 sc-25269 Santa Cruz Biotechnologies (SCBT)

anti-DBH 1:400 sc-15318 SCBT

anti-CHGA 1:4,000 A430 Dako

anti-CHGB 1:4,000 sc-1489 SCBT

anti-PH3 1:5,000 9706 Cell Signaling

anti-GFP 1:1,000 A6455 Invitrogen

anti-caspase3 1:500 9661 Cell Signaling

anti-RET 1:1000 3223 Cell Signaling

anti-NMYC 1:1000 9405 Cell Signaling
